# Correction: Targeting A-kinase anchoring protein 12 phosphorylation in hepatic stellate cells regulates liver injury and fibrosis in mouse models

**DOI:** 10.7554/eLife.88586

**Published:** 2023-04-18

**Authors:** Komal Ramani, Nirmala Mavila, Aushinie Abeynayake, Maria Lauda Tomasi, Jiaohong Wang, Michitaka Matsuda, Eki Seki

**Keywords:** Human, Mouse

Ramani K, Mavila N, Abeynayake A, Tomasi ML, Wang J, Matsuda M, Seki E. 2022. Targeting A-kinase anchoring protein 12 phosphorylation in hepatic stellate cells regulates liver injury and fibrosis in mouse models. *eLife* **11**:e78430. doi: 10.7554/eLife.78430.

Published 4 October 2022

We recently detected an error in our published figure 2D that was also confirmed by a reader in PubPeer. We accidentally copied a fluorescence image belonging to a test sample (Day5 +HDR) into the control Day 0 sample instead of using the original Day 0 image. This error likely occurred while we were adding scale bars to the individual fluorescent images after initially reading them. We believe that this error originated during transfer of the individual scaled images to create a picture. We could confirm this error by comparing to the corresponding brightfield images that were different between the two samples despite the fluorescent images looking same as was pointed out by the PubPeer reader.

We have now corrected the error by reporting the original Day 0 fluorescence image that corresponds to its brightfield image. We are also including a source data file containing the individual fluorescence and brightfield images represented in the corrected picture.

This change does not affect the overall results or conclusion of the paper.

Since we have added source data for this figure, the cited figure legend now contains a reference for this source data:

Figure 2 (D). Day 0 attached HSCs were culture activated till Day 3 and then transfected with CRISPR vectors till Day 5. The autofluorescence of vitamin A as a marker of HSC quiescence was visualized by fluorescence microscopy and compared to brightfield images of cells as in Materials and methods. Three independent experiments are shown. Scale bar = 80 µm. **Source data is presented in Figure 2—source data 5.**

Original text within the figure legend:

Figure 2 (D). Day 0 attached HSCs were culture activated till Day 3 and then transfected with CRISPR vectors till Day 5. The autofluorescence of vitamin A as a marker of HSC quiescence was visualized by fluorescence microscopy and compared to brightfield images of cells as in Materials and methods. Three independent experiments are shown. Scale bar = 80 µm.

The corrected figure (panel 2D) is shown below:

**Figure 1:**
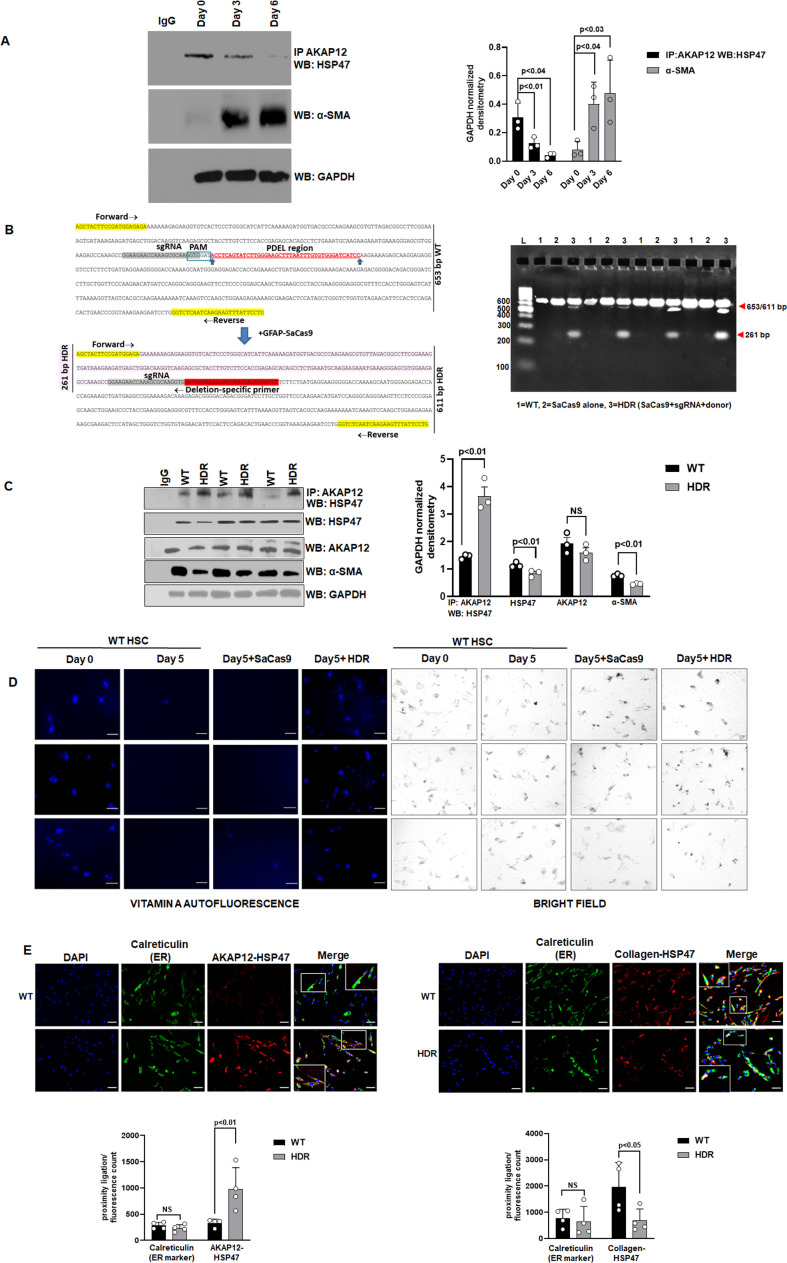


The original figure (panel 2D) is shown below:

**Figure 2:**
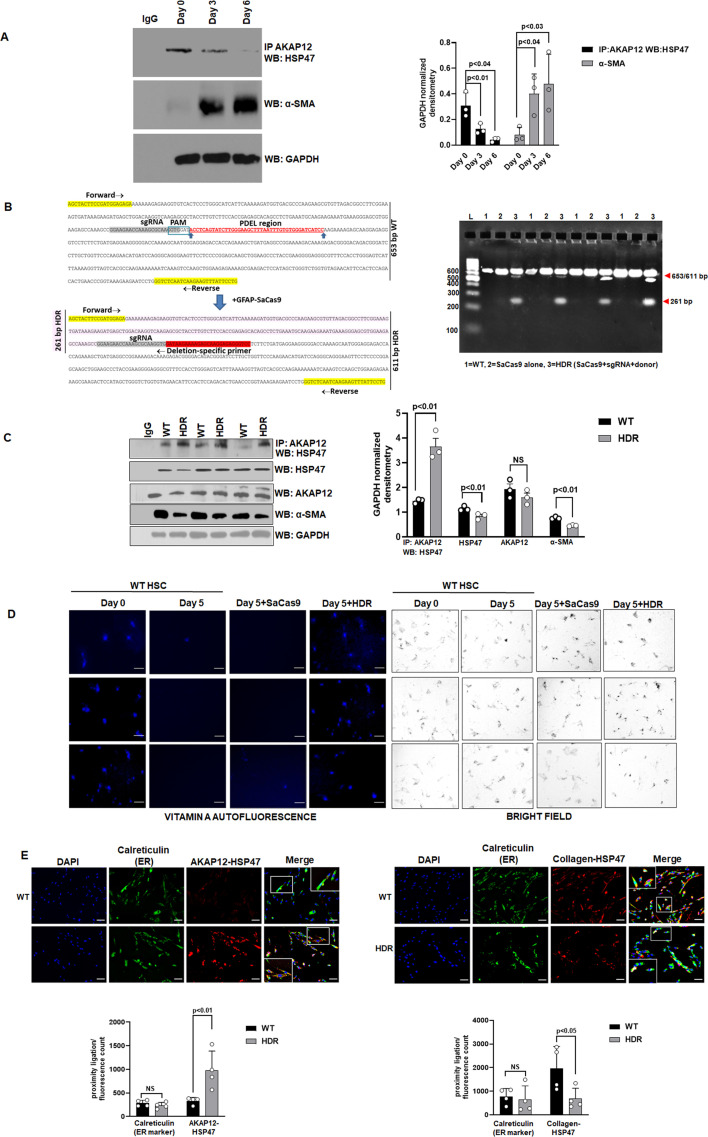


Corrected image panel is compared to the original below:

**Figure 3:**
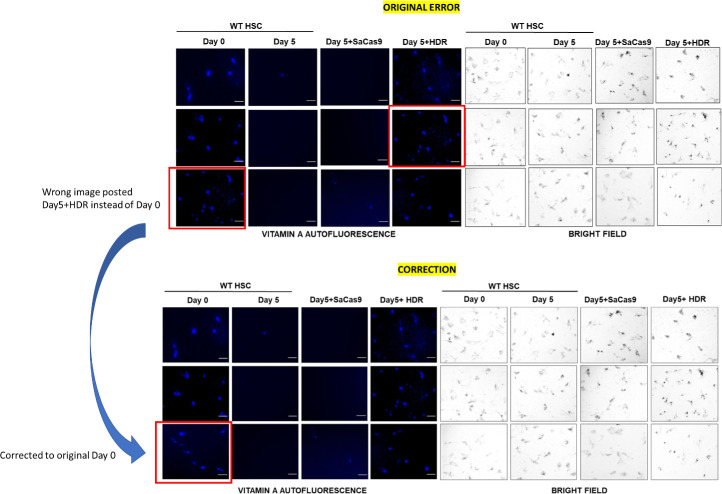


The article has been corrected accordingly.

