# Supplementary figures and images for: Targeting A-kinase anchoring protein 12 phosphorylation in hepatic stellate cells regulates liver injury and fibrosis in mouse models
Source: eLife. 2022 Oct 4;11:e78430. doi: 10.7554/eLife.78430 (PMC9531947; doi:10.7554/eLife.78430)

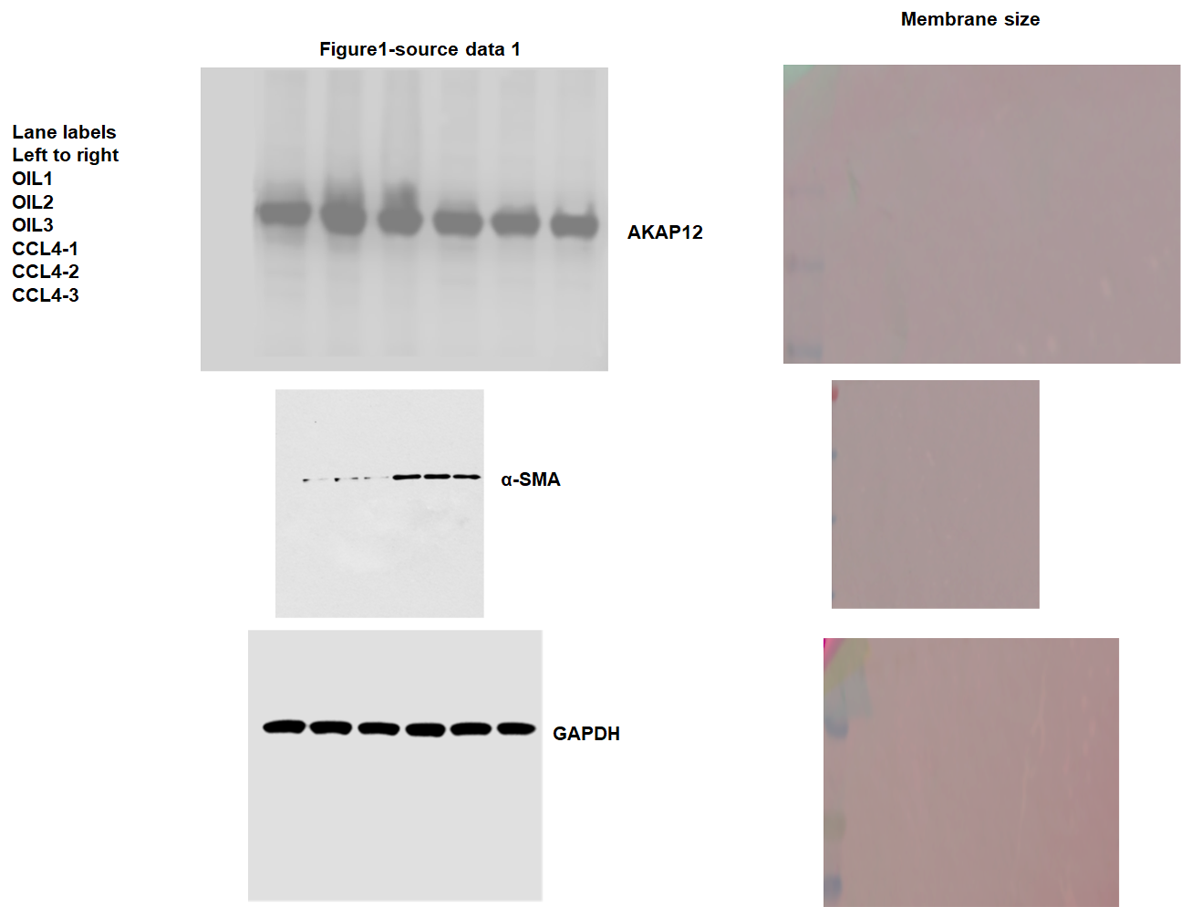

Supplement: Figure 1—source data 1. [file elife-78430-fig1-data1.zip › Figure 1-source data 1/figure 1-source data 1.tif]

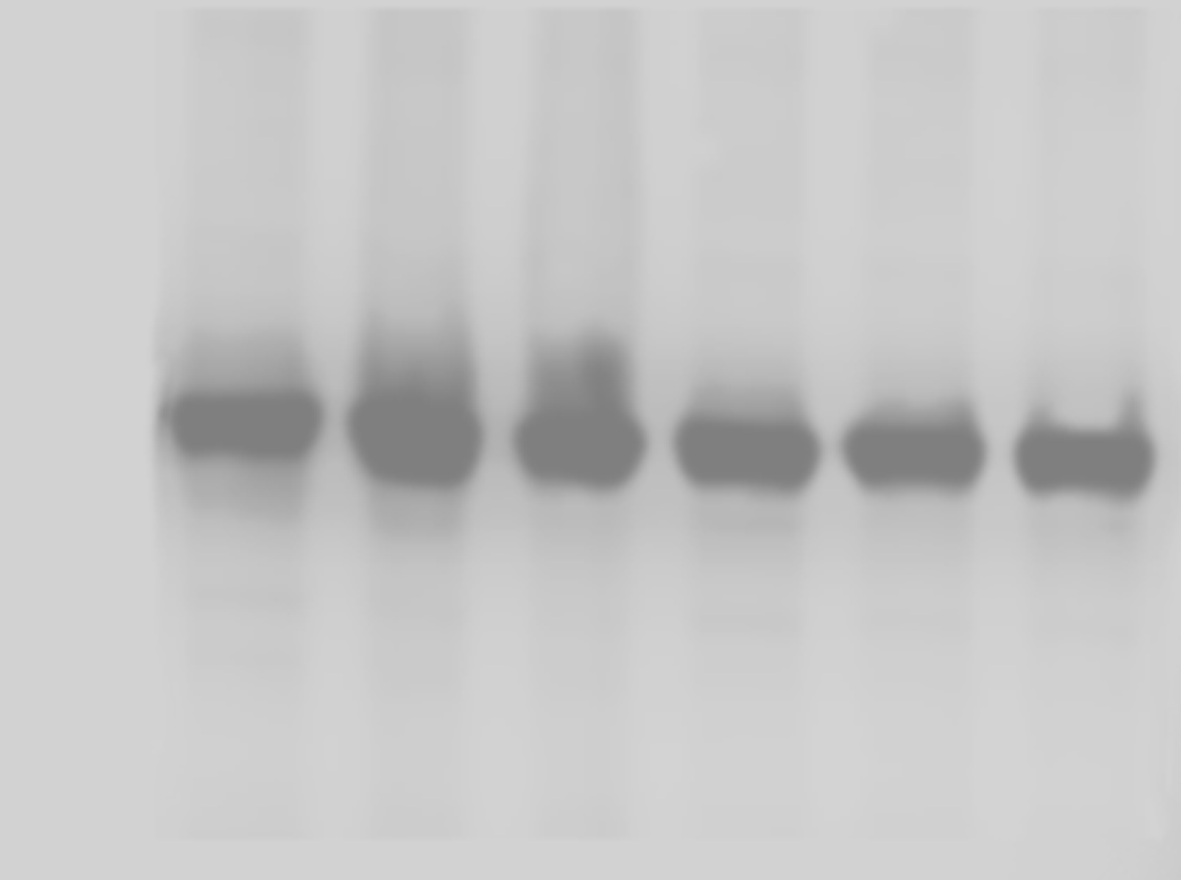

Supplement: Figure 1—source data 1. [file elife-78430-fig1-data1.zip › Figure 1-source data 1/unlabeled fig1-source data-1-AKAP12.tif]

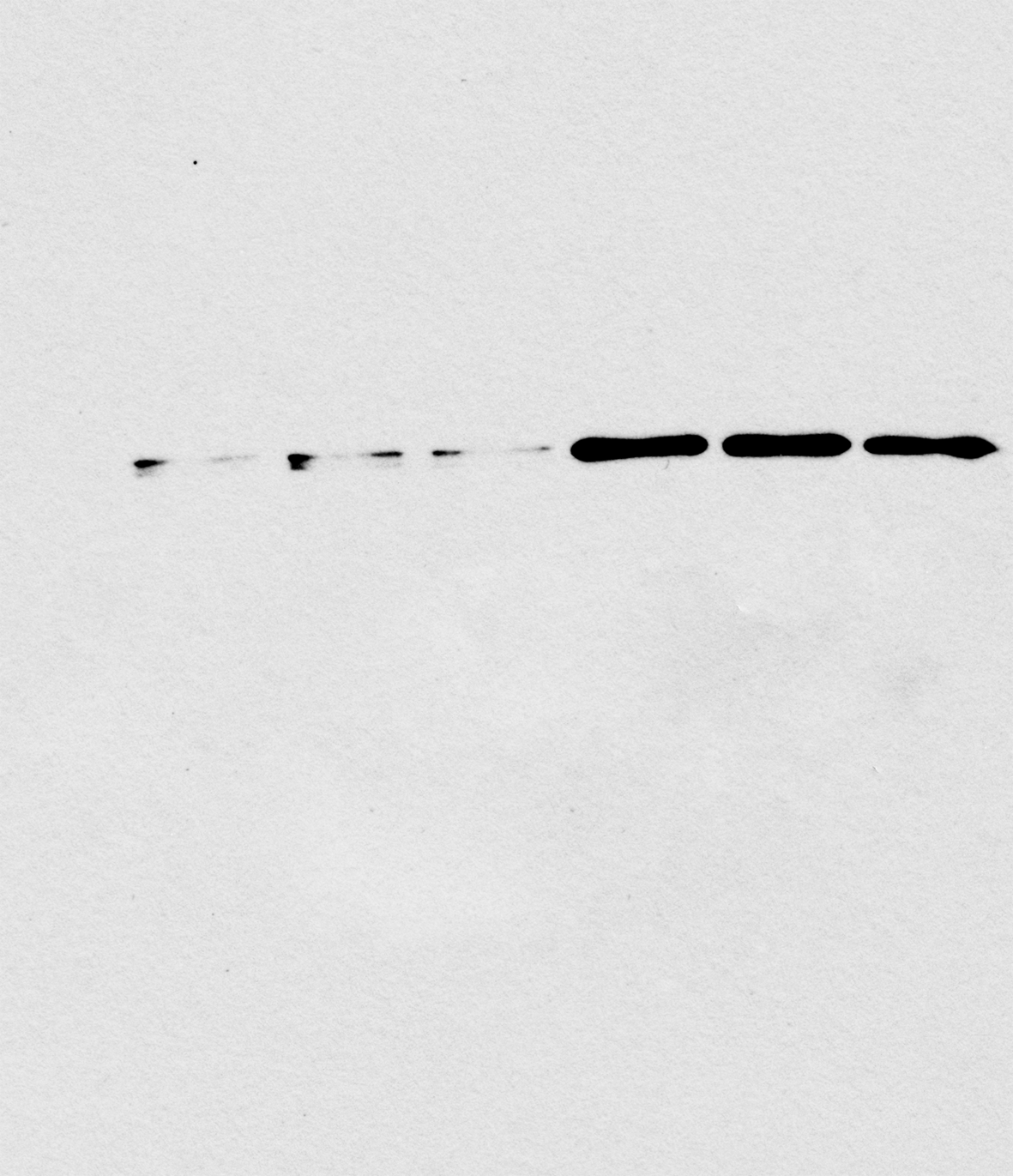

Supplement: Figure 1—source data 1. [file elife-78430-fig1-data1.zip › Figure 1-source data 1/unlabeled fig1-source data-1-alpha-SMA.tif]

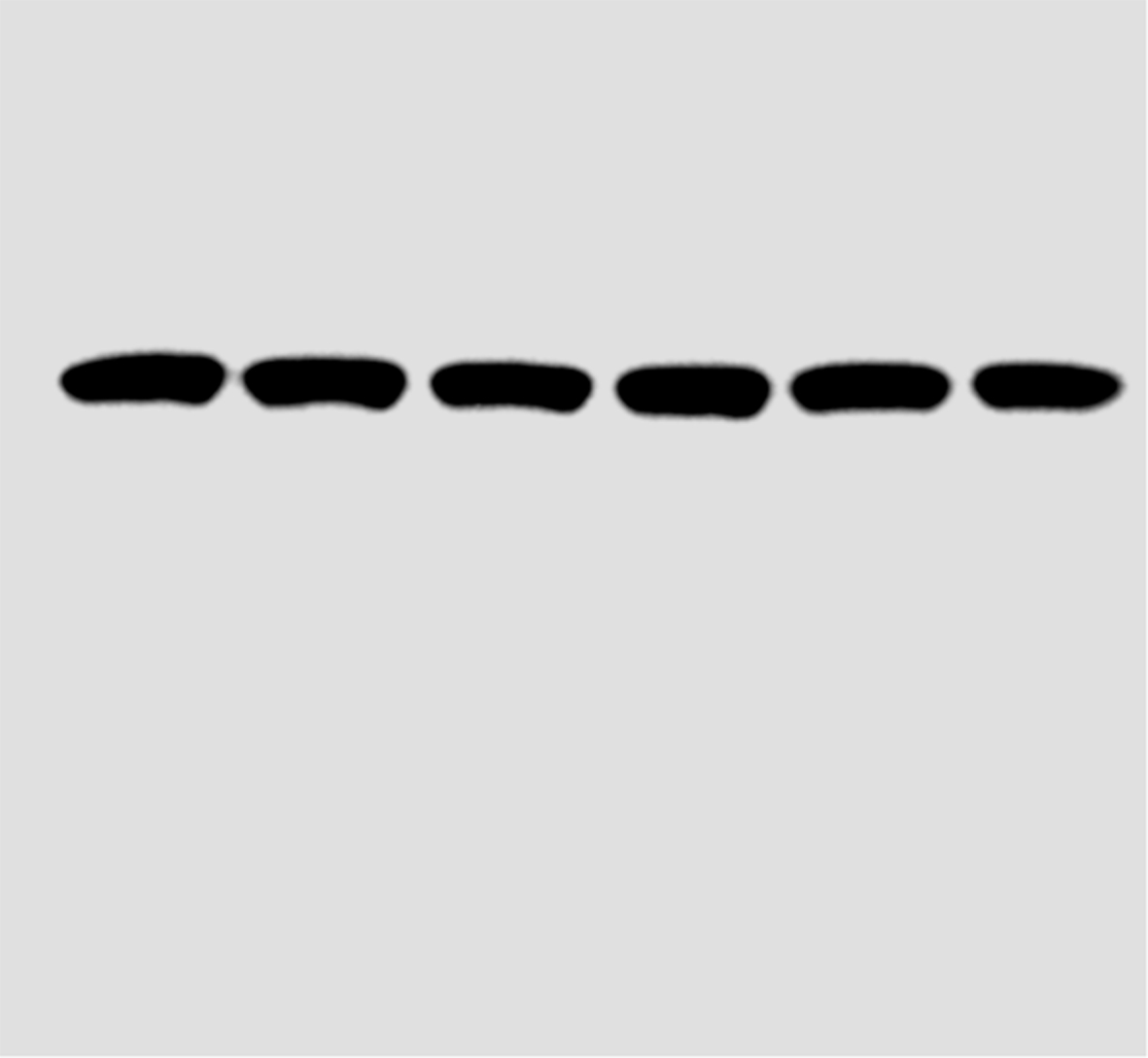

Supplement: Figure 1—source data 1. [file elife-78430-fig1-data1.zip › Figure 1-source data 1/unlabeled fig1-source data-1-GAPDH.tif]

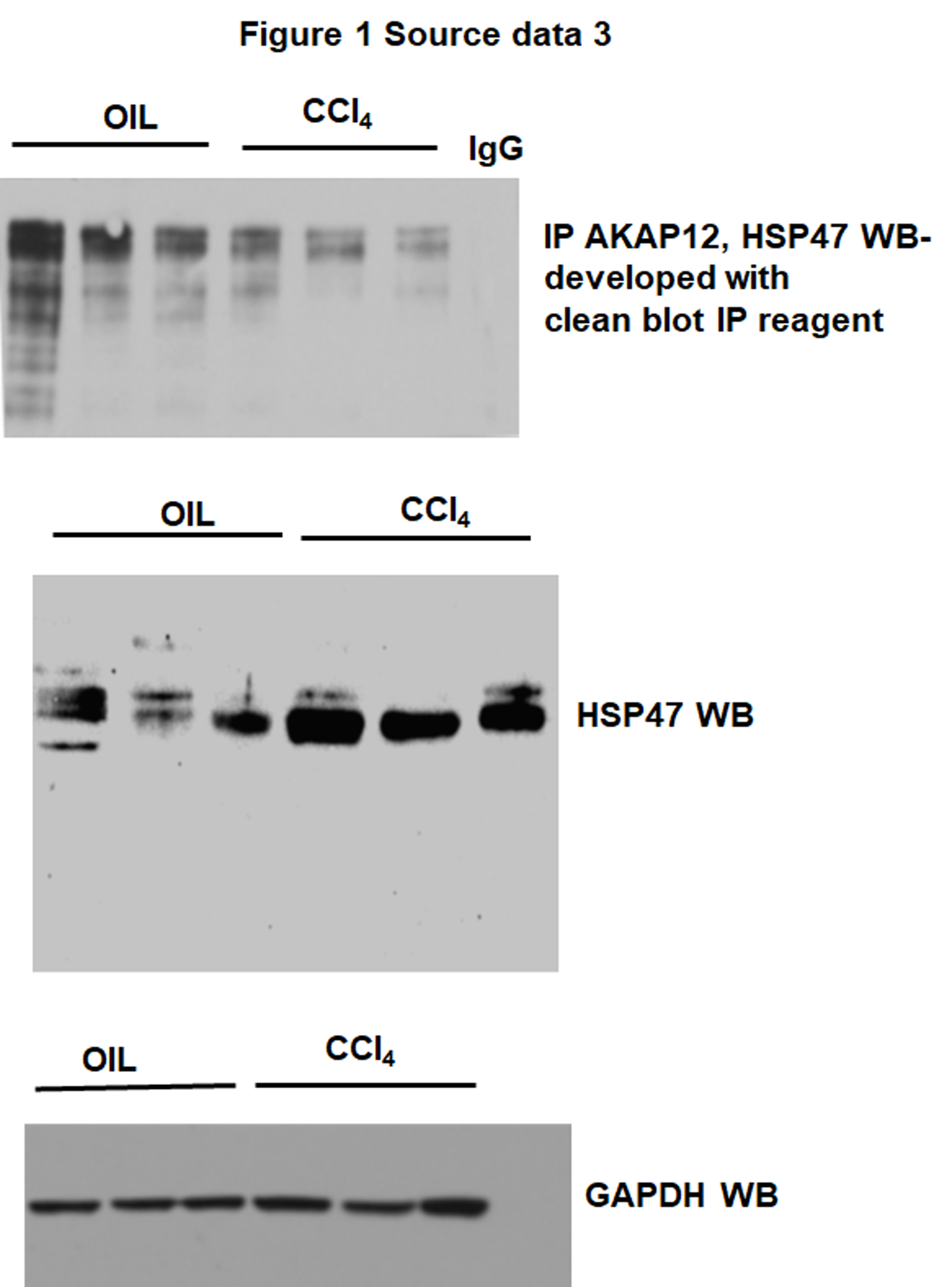

Supplement: Figure 1—source data 3. [file elife-78430-fig1-data3.zip › Figure 1-source data 3/figure 1-source data 3.tif]

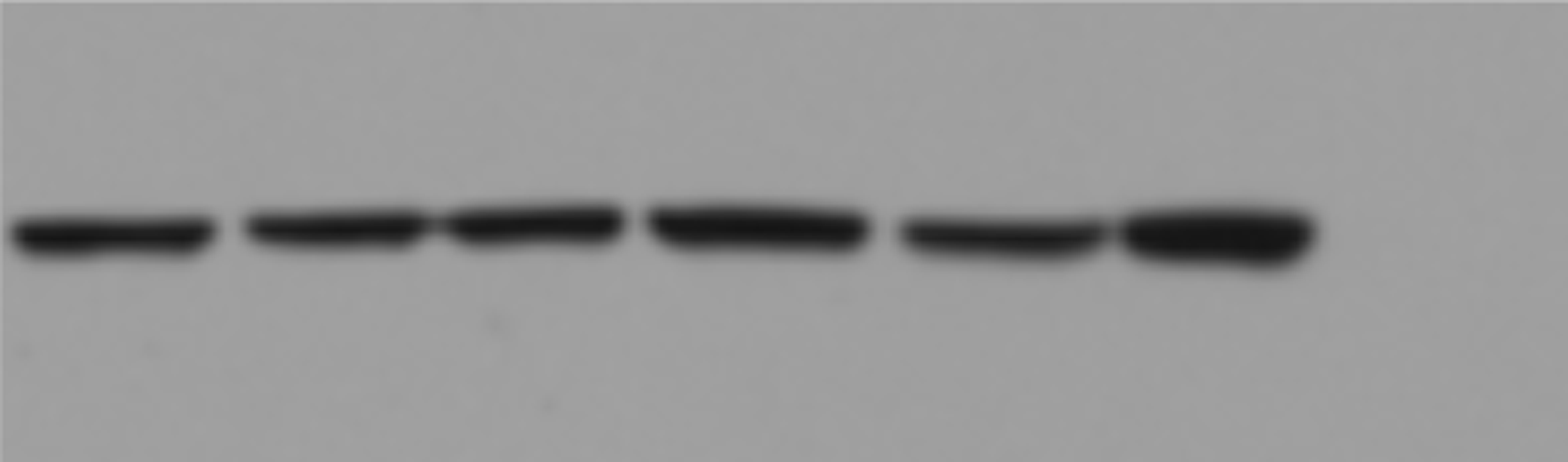

Supplement: Figure 1—source data 3. [file elife-78430-fig1-data3.zip › Figure 1-source data 3/unlabeled Figure 1-source data 3-GAPDH.tif]

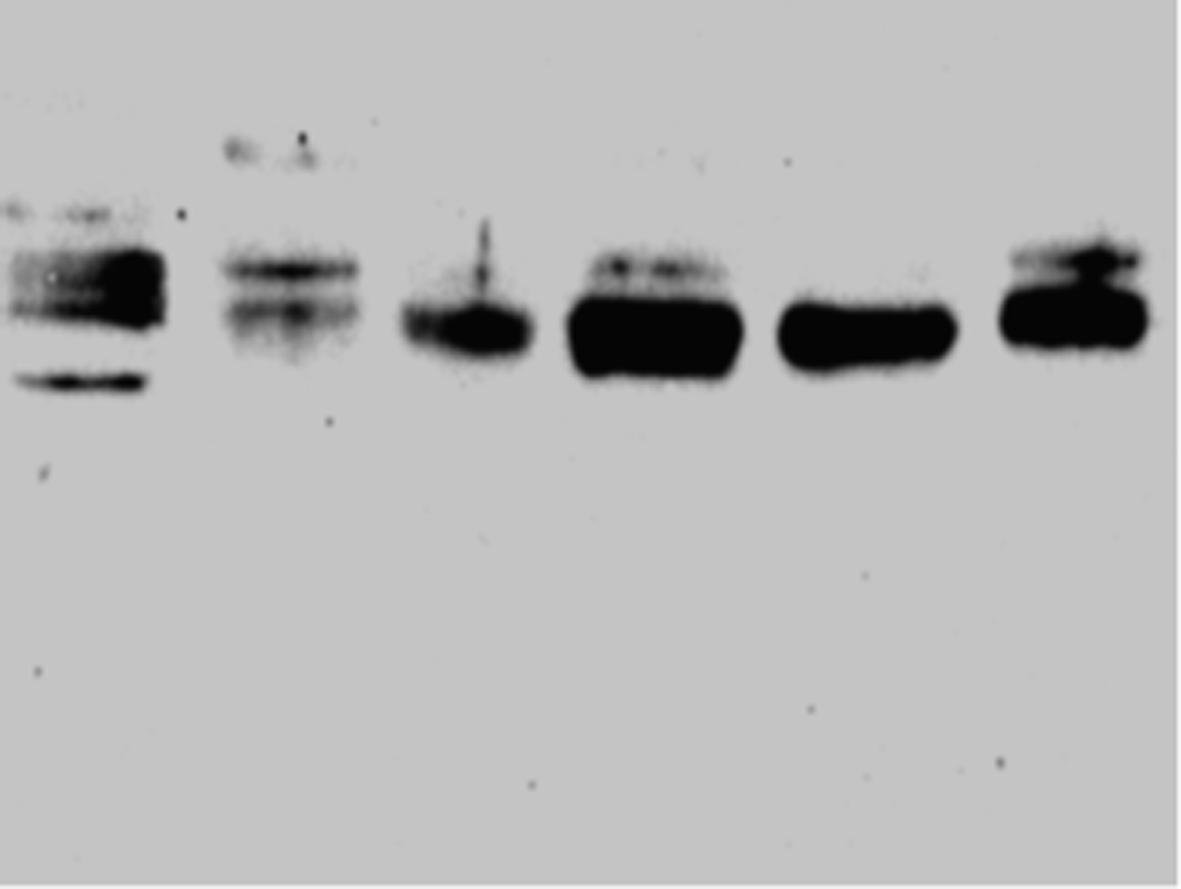

Supplement: Figure 1—source data 3. [file elife-78430-fig1-data3.zip › Figure 1-source data 3/unlabeled Figure 1-source data 3-HSP47.tif]

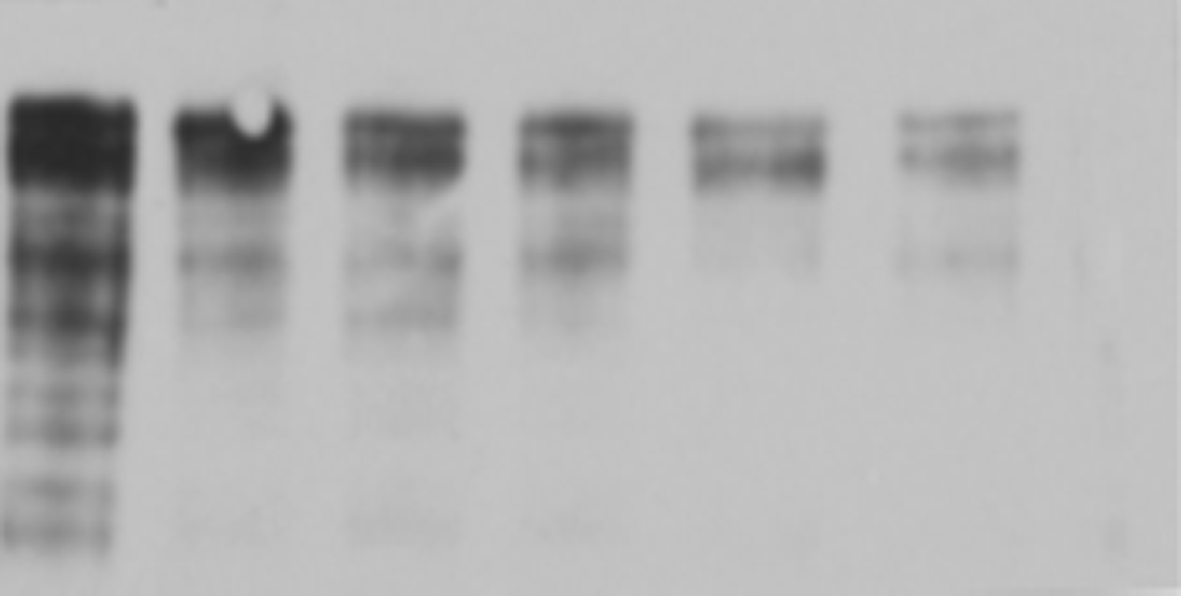

Supplement: Figure 1—source data 3. [file elife-78430-fig1-data3.zip › Figure 1-source data 3/unlabeled Figure 1-source data 3-IP AKAP12 WB HSP47.tif]

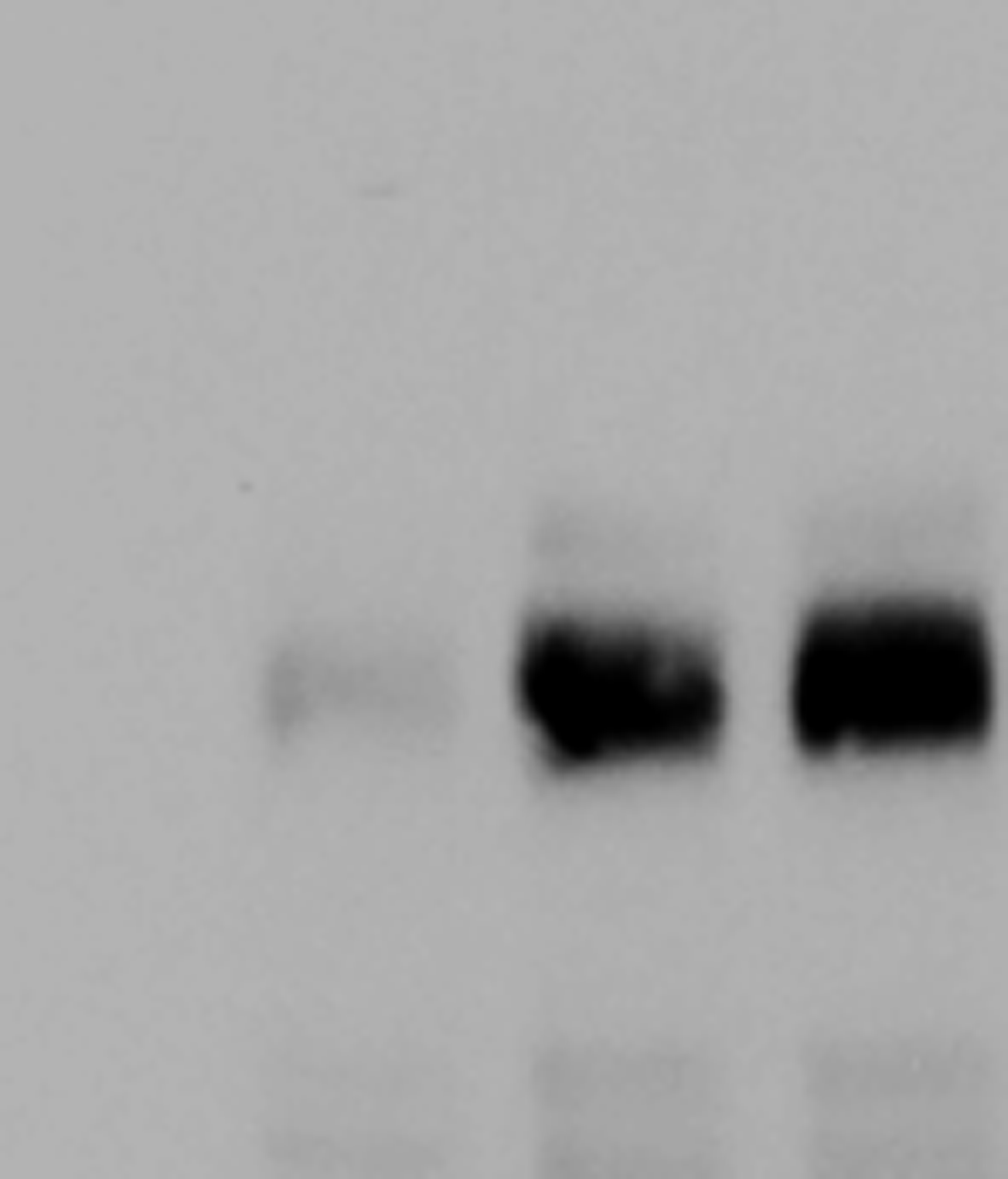

Supplement: Figure 2—source data 1. [file elife-78430-fig2-data1.zip › figure 2-source data 1/fig 2-source data 1-alpha-SMA-exp1.tif]

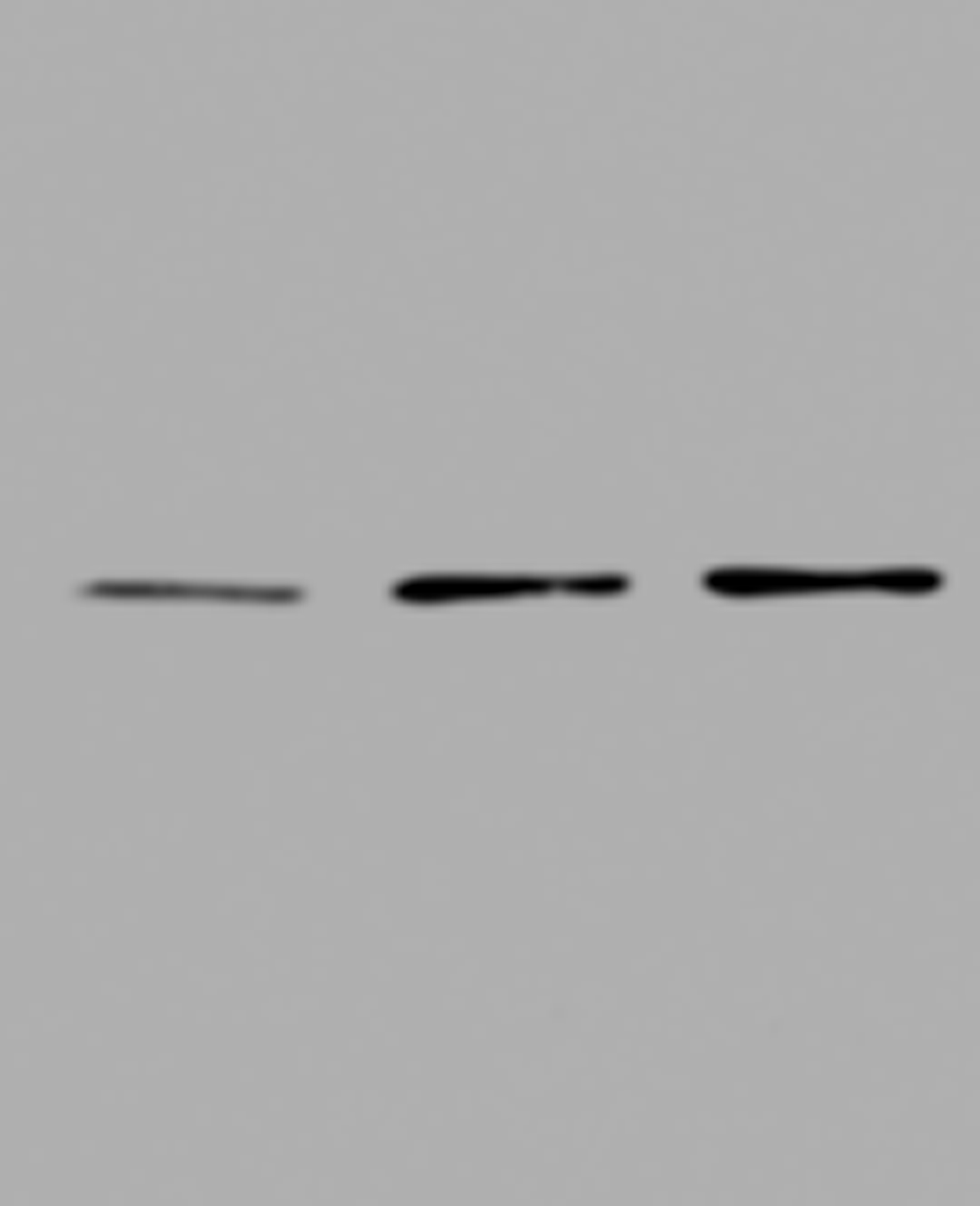

Supplement: Figure 2—source data 1. [file elife-78430-fig2-data1.zip › figure 2-source data 1/fig 2-source data 1-alpha-SMA-exp2.tif]

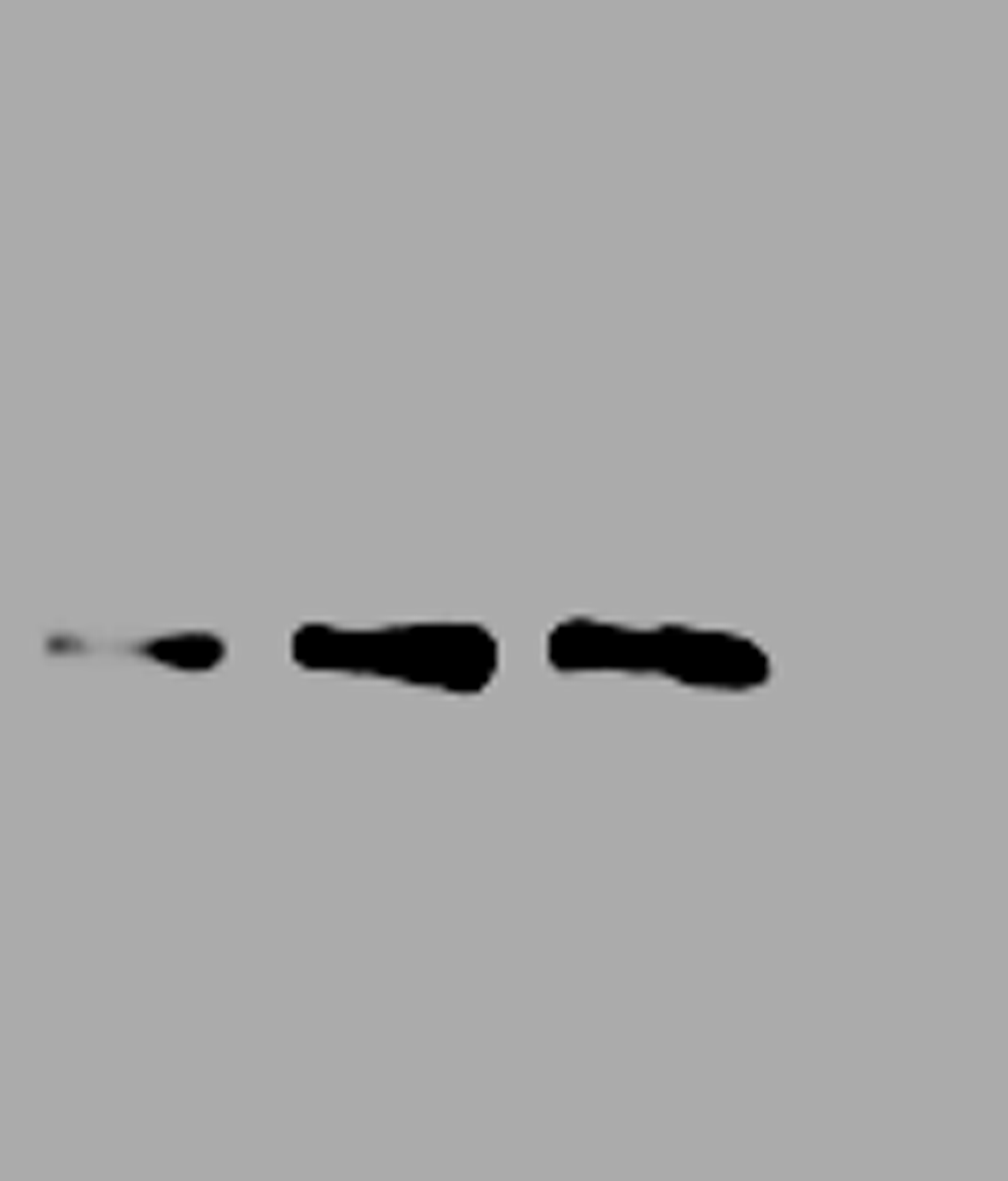

Supplement: Figure 2—source data 1. [file elife-78430-fig2-data1.zip › figure 2-source data 1/fig 2-source data 1-alpha-SMA-exp3.tif]

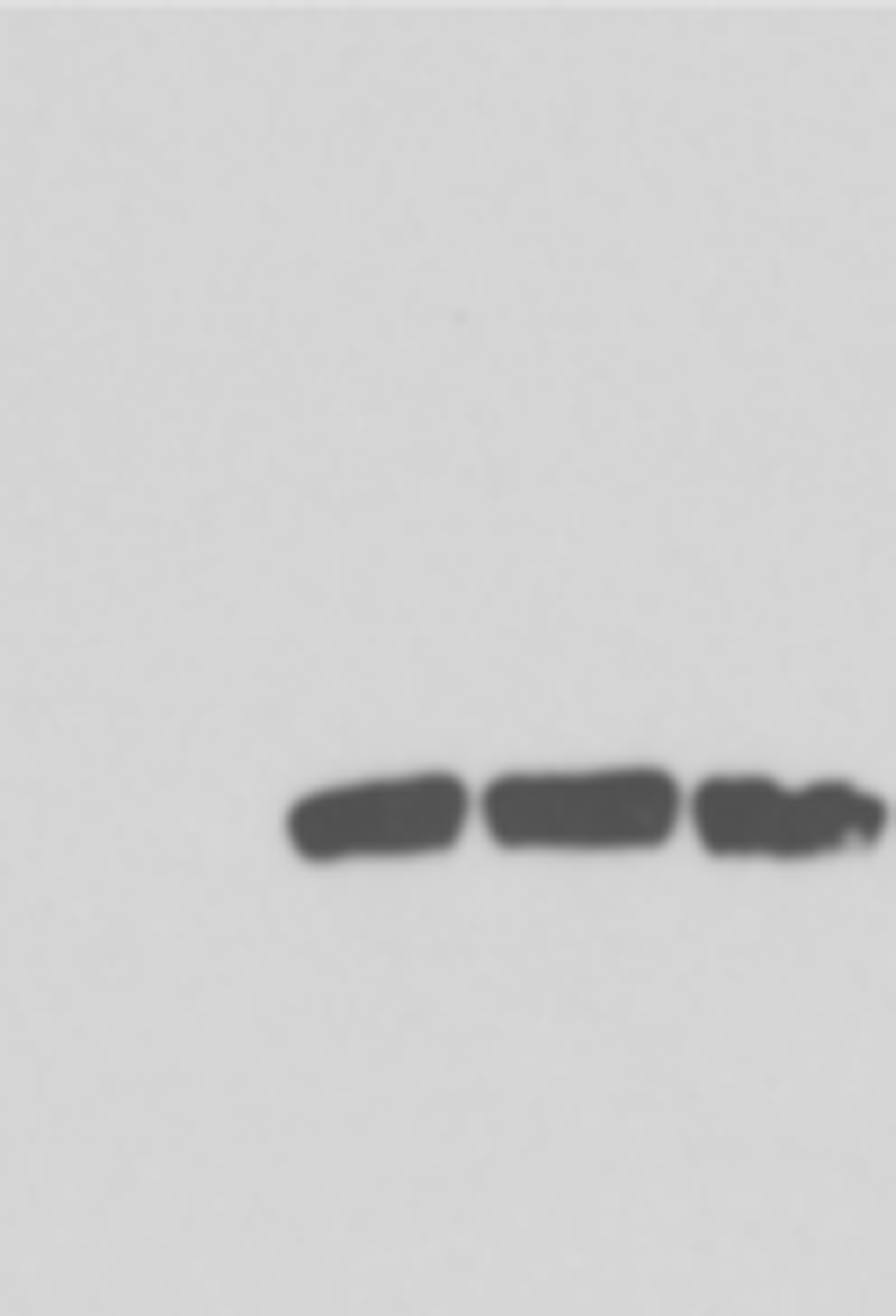

Supplement: Figure 2—source data 1. [file elife-78430-fig2-data1.zip › figure 2-source data 1/fig 2-source data 1-GAPDH-exp1.tif]

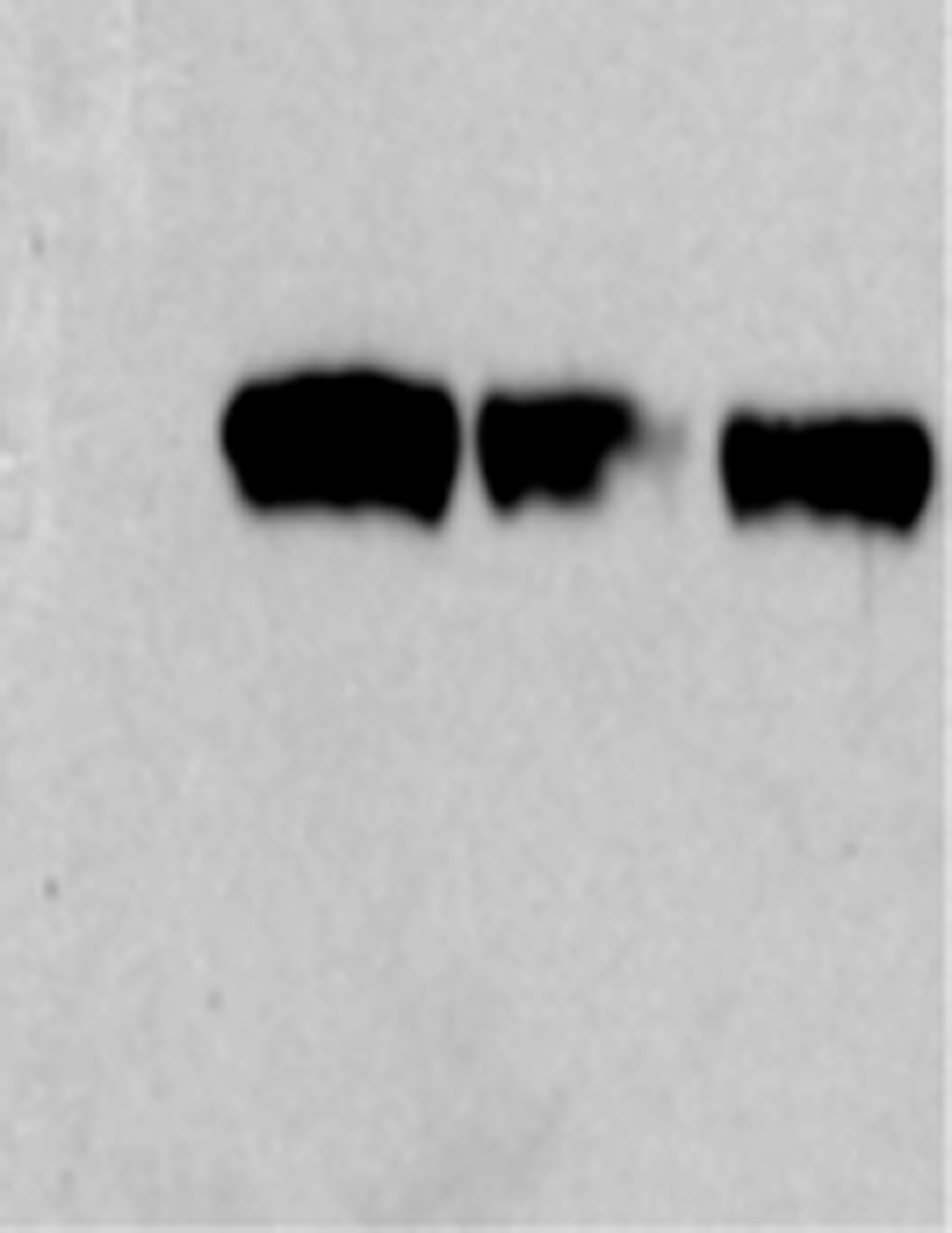

Supplement: Figure 2—source data 1. [file elife-78430-fig2-data1.zip › figure 2-source data 1/fig 2-source data 1-GAPDH-exp2.tif]

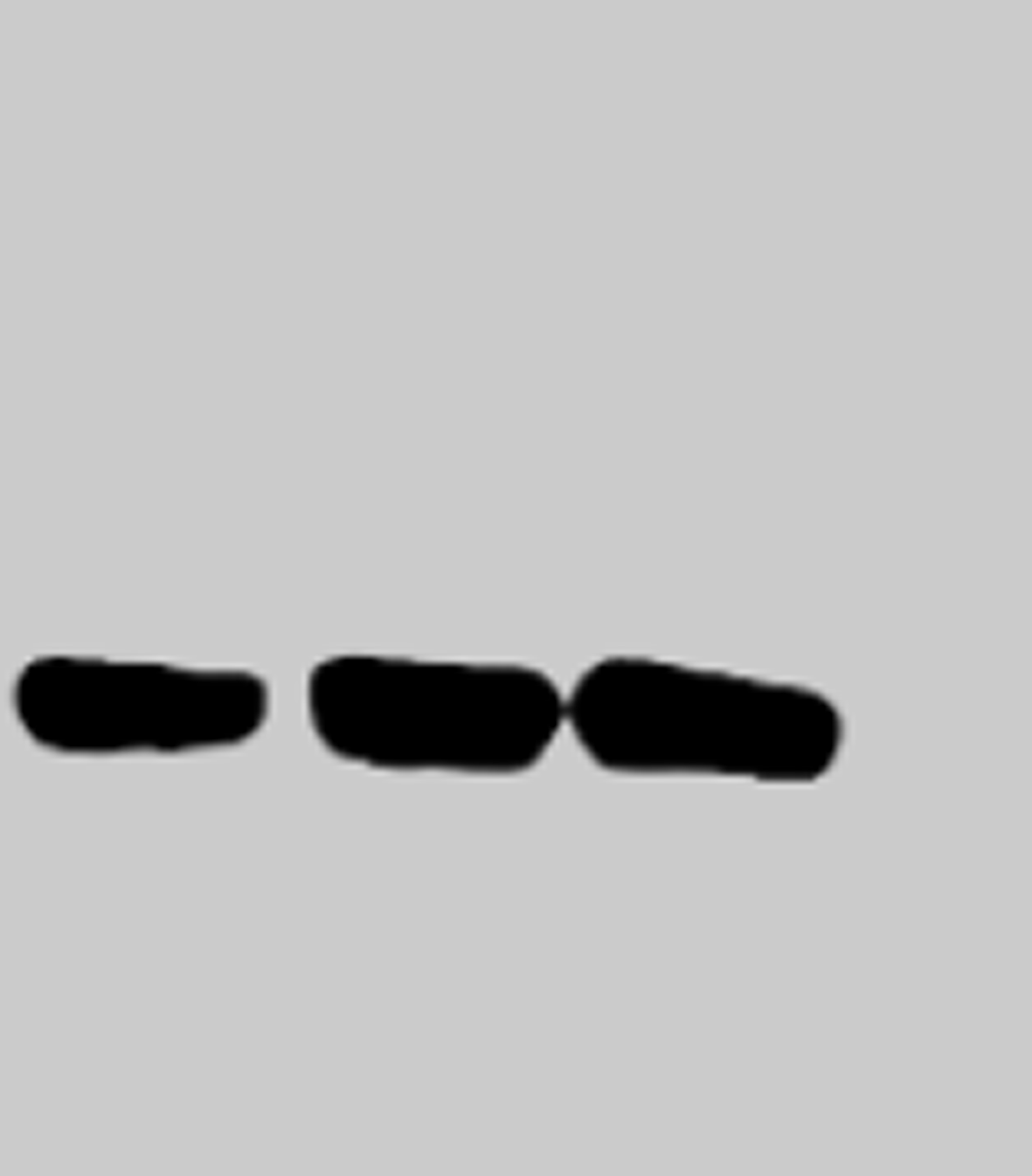

Supplement: Figure 2—source data 1. [file elife-78430-fig2-data1.zip › figure 2-source data 1/fig 2-source data 1-GAPDH-exp3.tif]

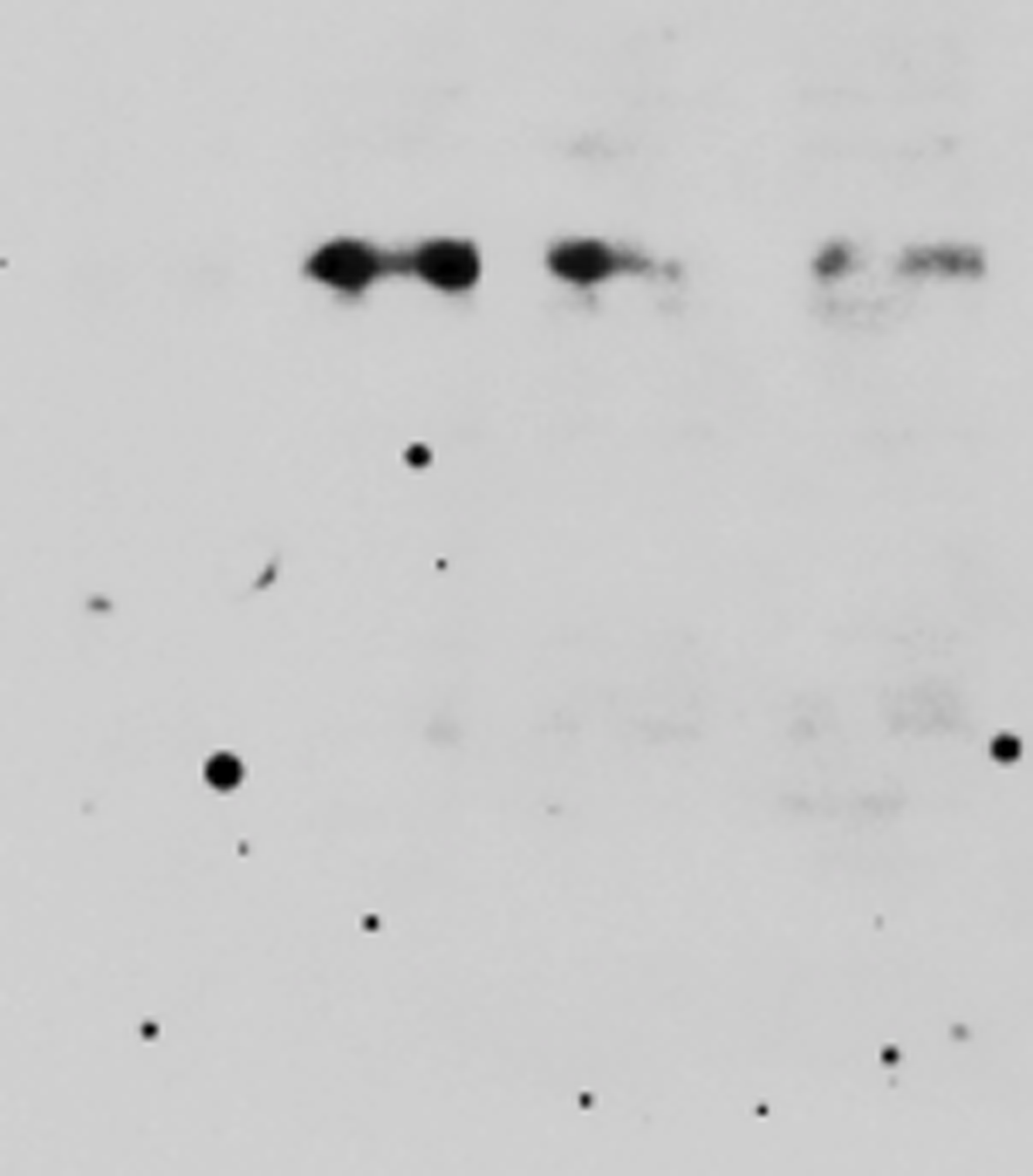

Supplement: Figure 2—source data 1. [file elife-78430-fig2-data1.zip › figure 2-source data 1/fig 2-source data 1-IP AKAP12 WB HSP47-exp 2.tif]

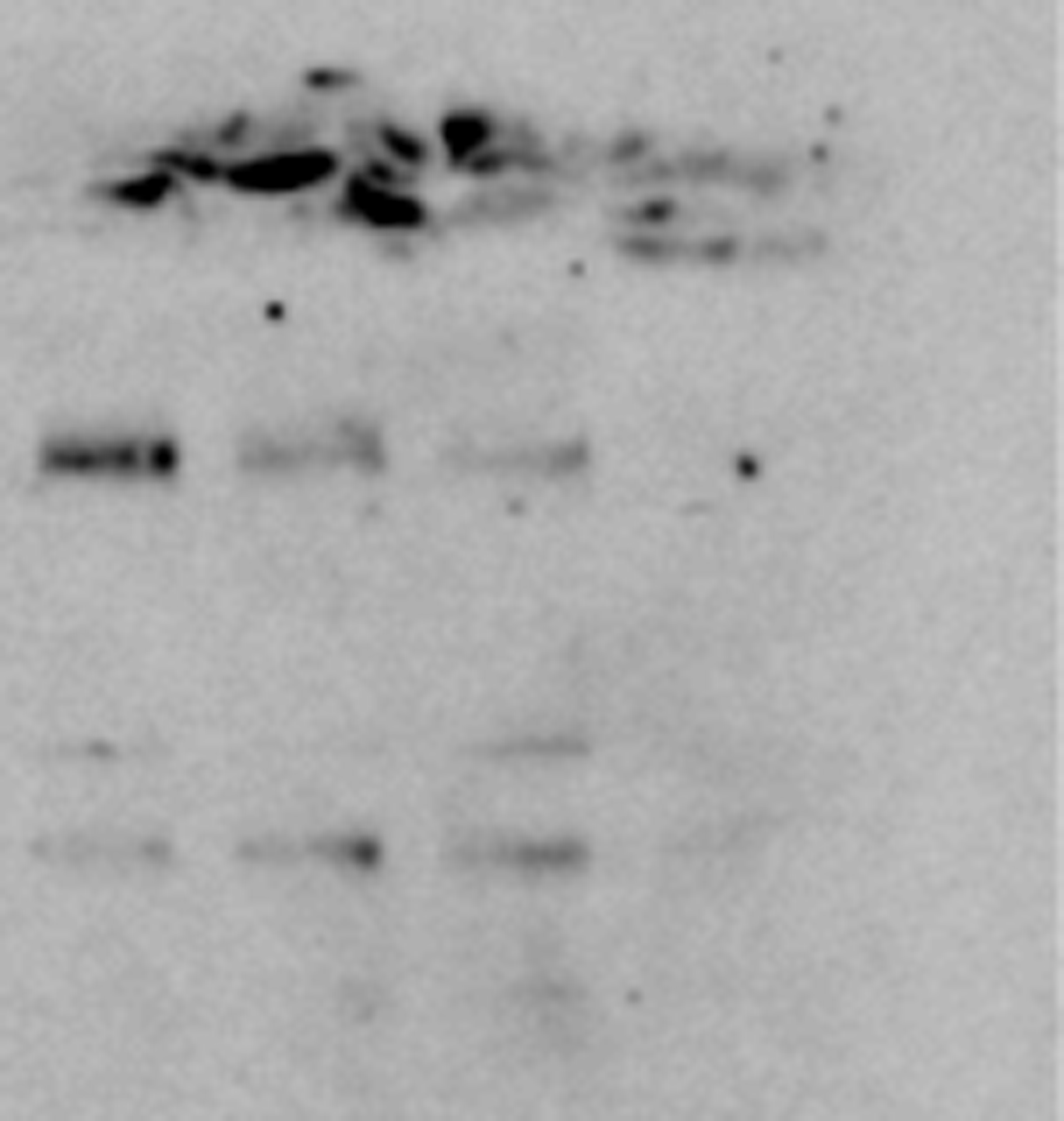

Supplement: Figure 2—source data 1. [file elife-78430-fig2-data1.zip › figure 2-source data 1/fig 2-source data 1-IP AKAP12 WB HSP47-exp 3.tif]

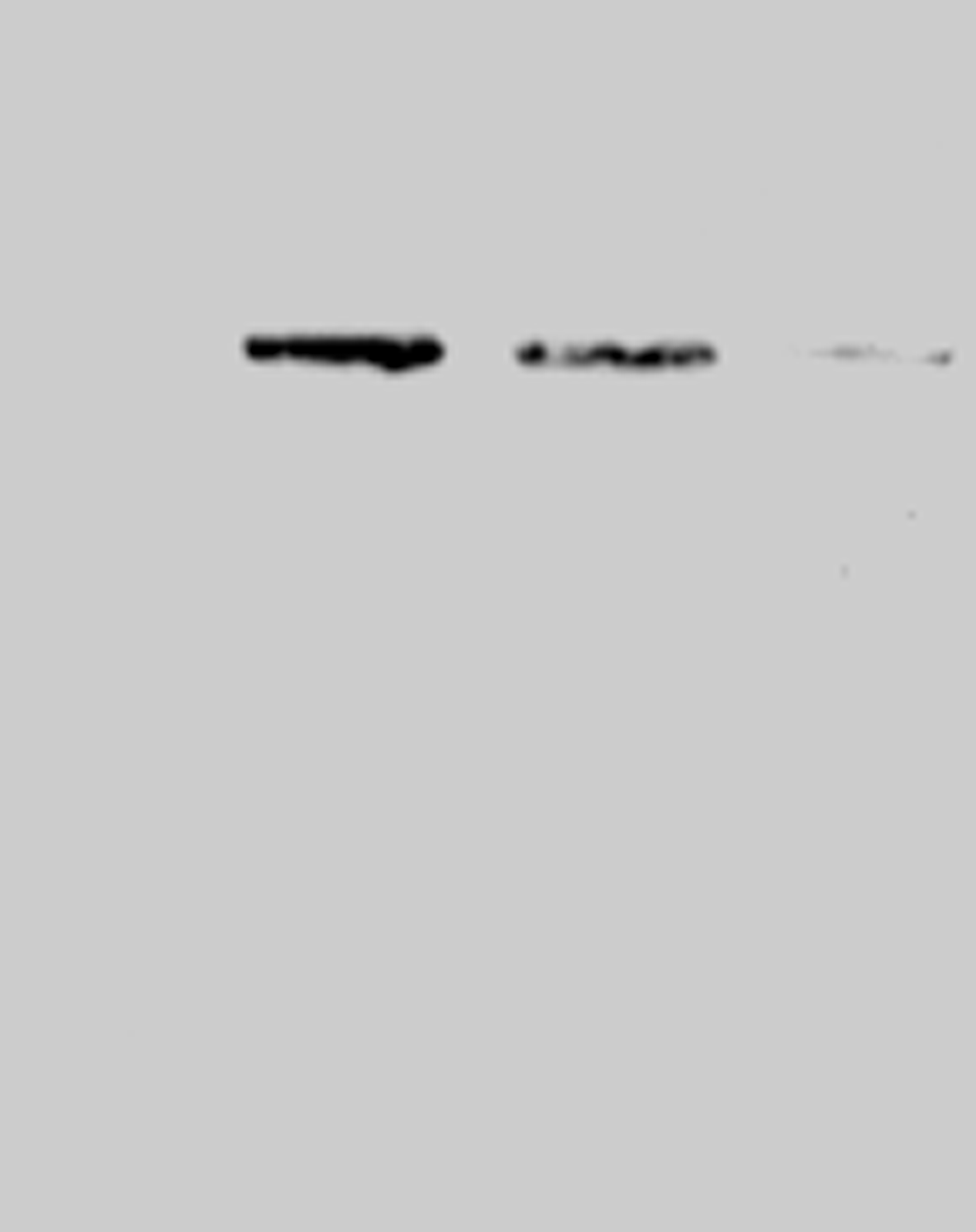

Supplement: Figure 2—source data 1. [file elife-78430-fig2-data1.zip › figure 2-source data 1/fig 2-source data 1-IP AKAP12 WB HSP47-exp1.tif]

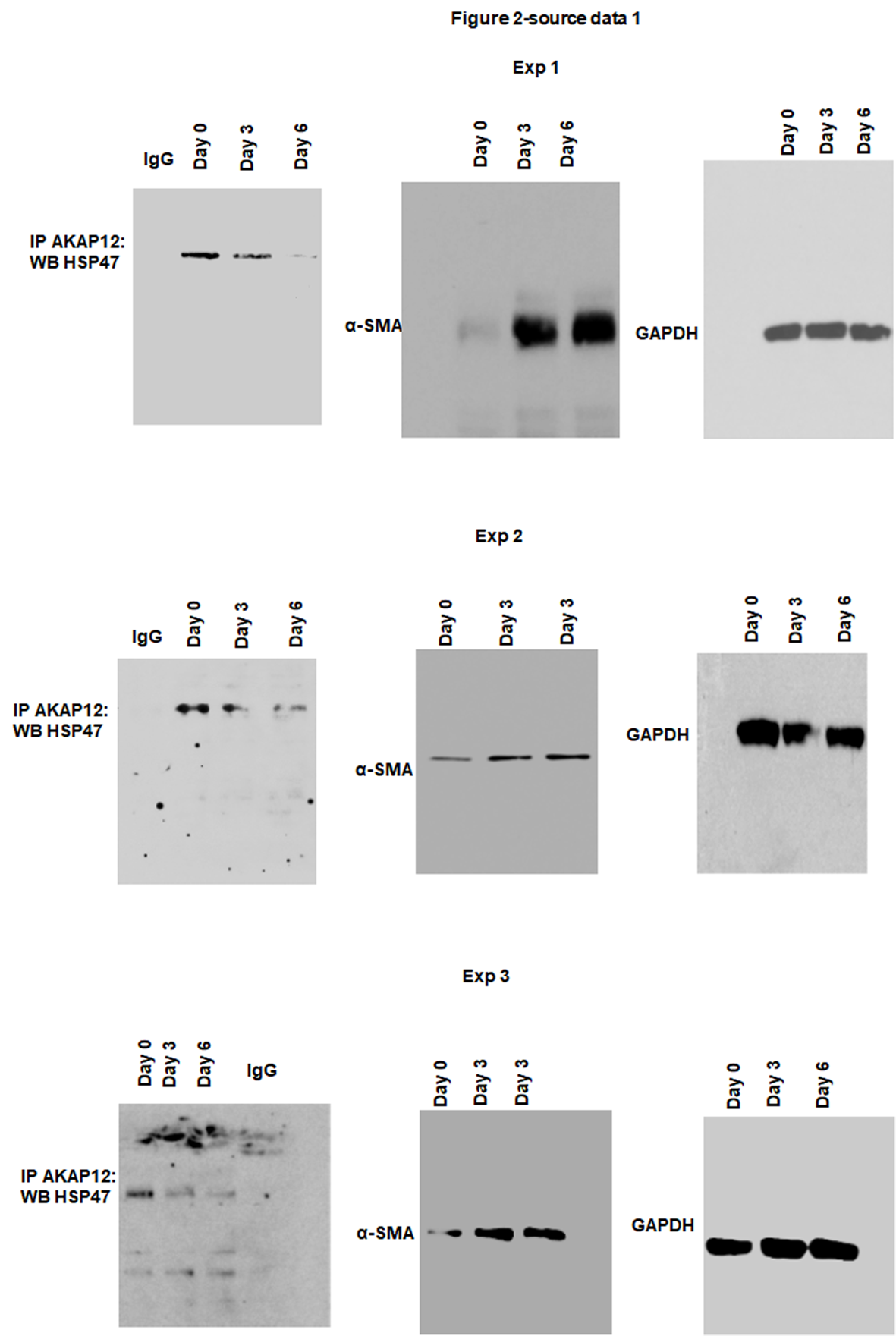

Supplement: Figure 2—source data 1. [file elife-78430-fig2-data1.zip › figure 2-source data 1/figure 2-source data 1.tif]

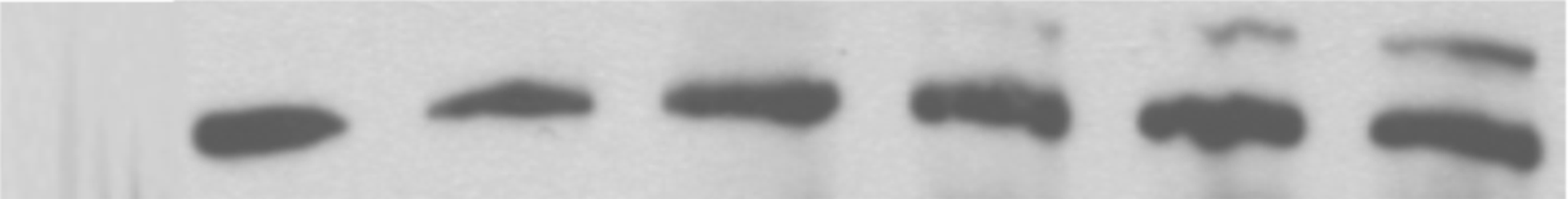

Supplement: Figure 2—source data 2. [file elife-78430-fig2-data2.zip › figure 2-source data 2/fig2-source data 2-AKAP12.tif]

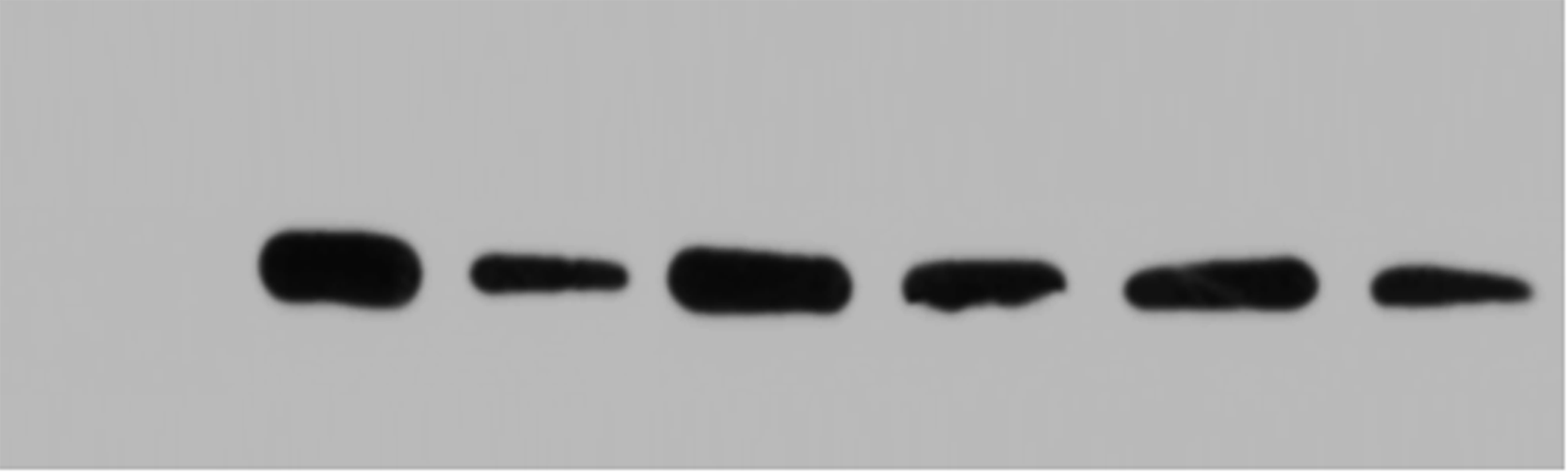

Supplement: Figure 2—source data 2. [file elife-78430-fig2-data2.zip › figure 2-source data 2/fig2-source data 2-alpha-SMA.tif]

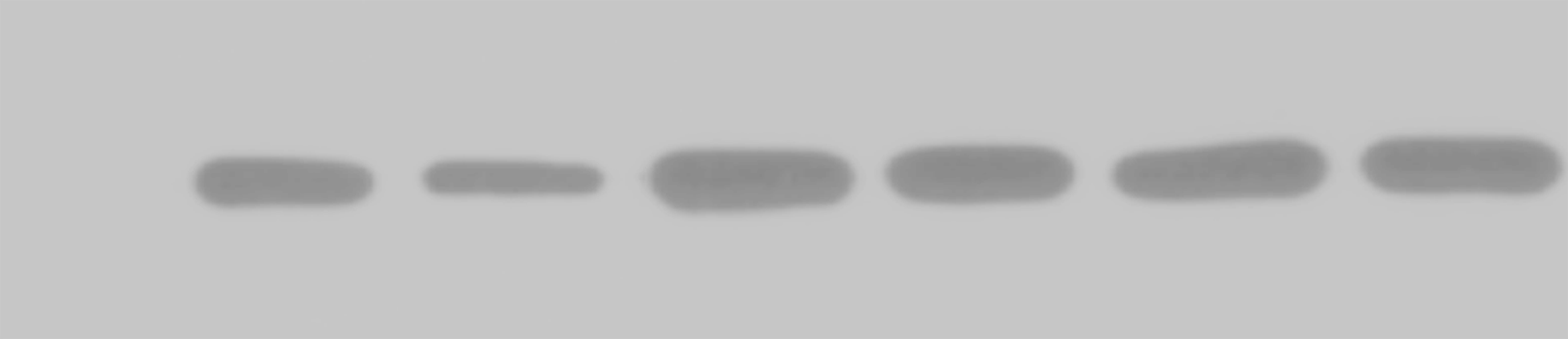

Supplement: Figure 2—source data 2. [file elife-78430-fig2-data2.zip › figure 2-source data 2/fig2-source data 2-HSP47.tif]

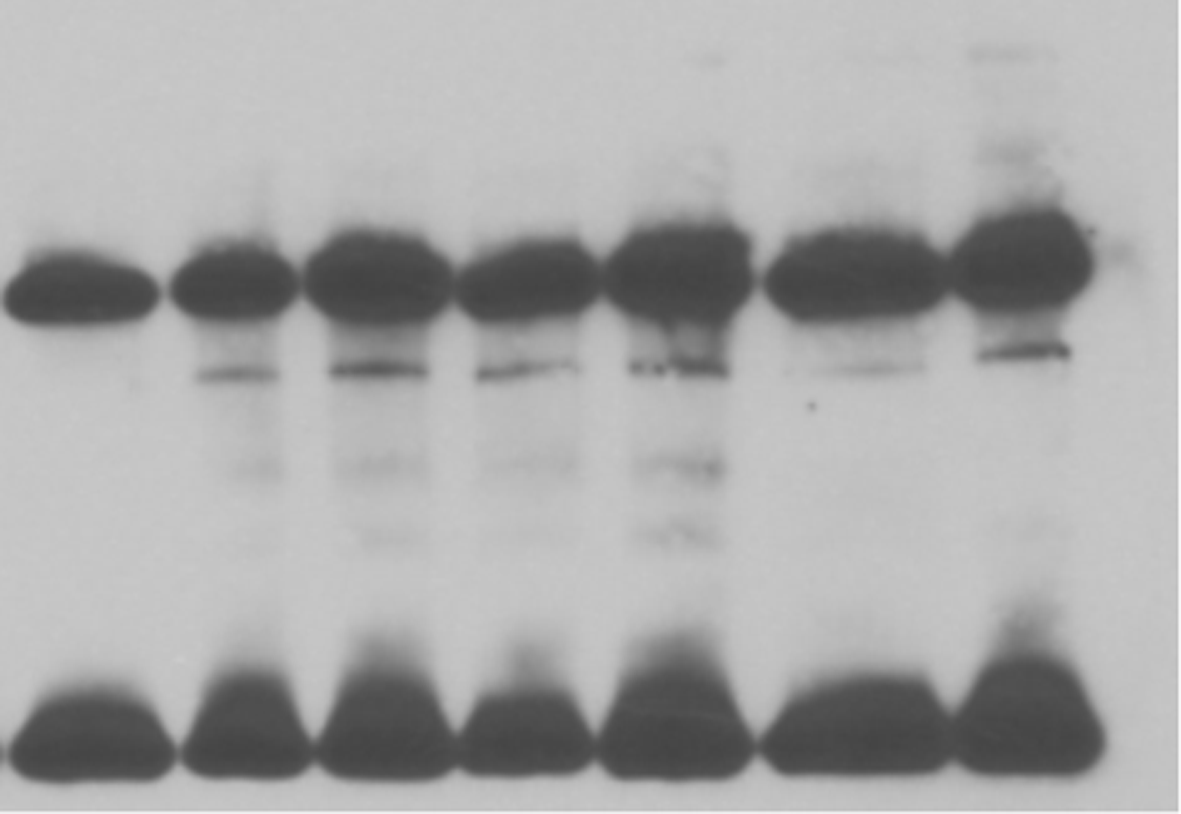

Supplement: Figure 2—source data 2. [file elife-78430-fig2-data2.zip › figure 2-source data 2/fig2-source data 2-IP AKAP12 WB HSP47.tif]

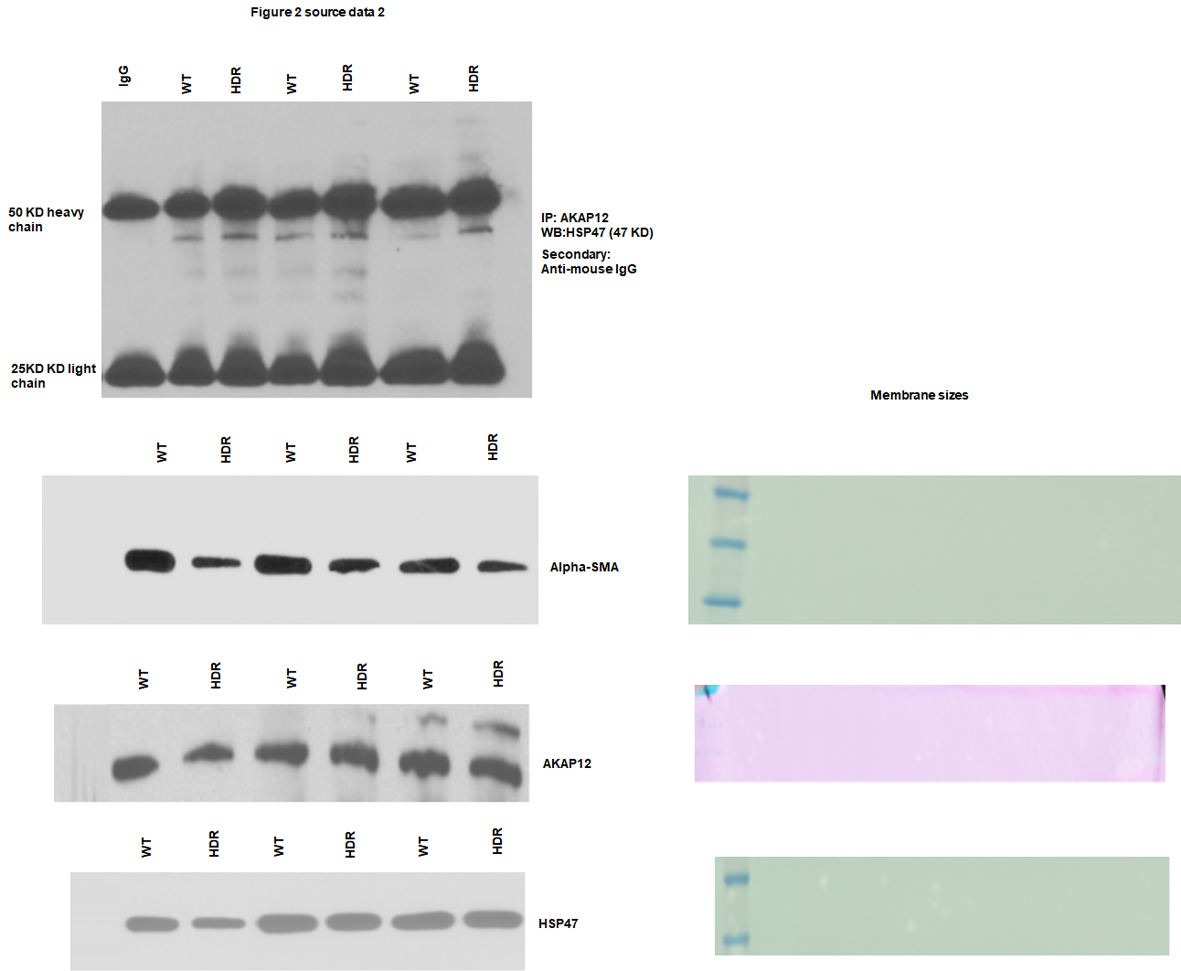

Supplement: Figure 2—source data 2. [file elife-78430-fig2-data2.zip › figure 2-source data 2/figure 2-source data 2.tif]

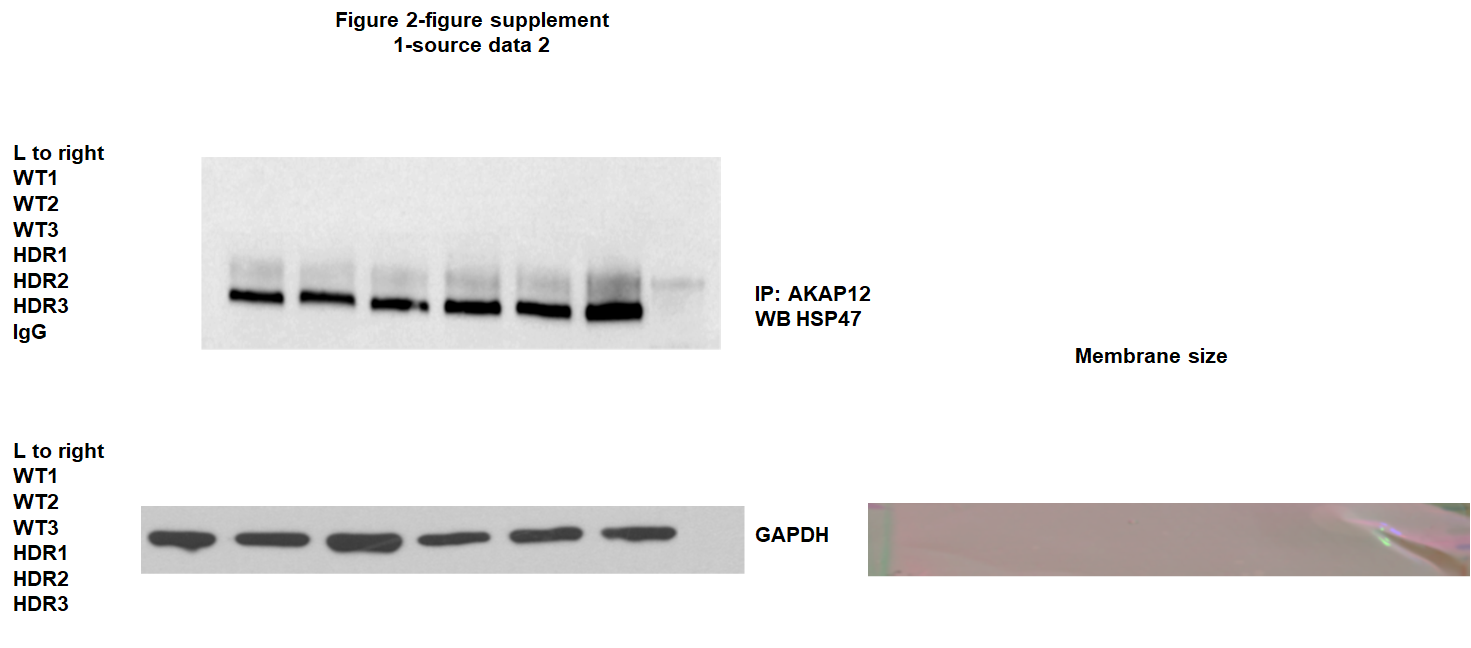

Supplement: Figure 2—figure supplement 1—source data 1. [file elife-78430-fig2-figsupp1-data1.zip › fig 2-figure supplement 1-source data 2/figure 2-figure supplement 1-source data 2.tif]

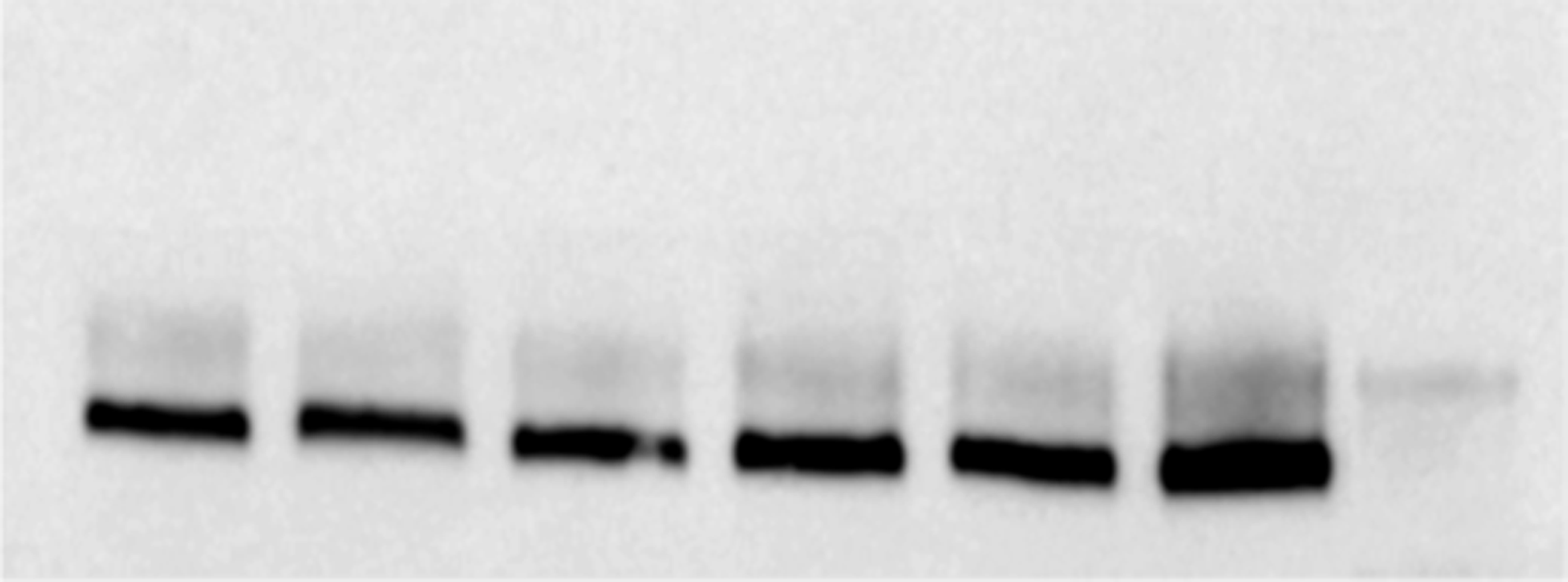

Supplement: Figure 2—figure supplement 1—source data 1. [file elife-78430-fig2-figsupp1-data1.zip › fig 2-figure supplement 1-source data 2/unlabeled fig 2 fig suppl 1-source data 2-IP AKAP12-HSP47.tif]

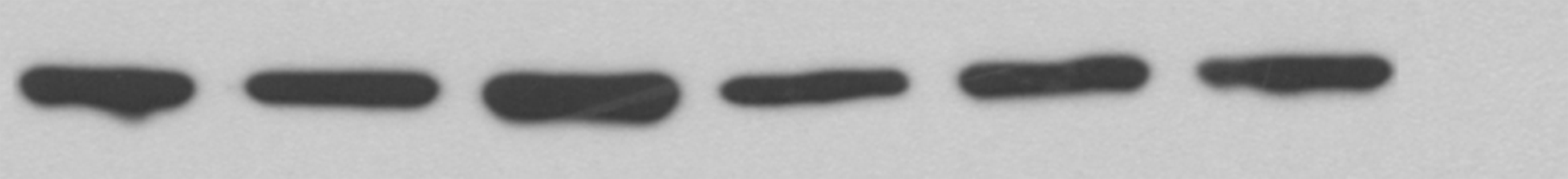

Supplement: Figure 2—figure supplement 1—source data 1. [file elife-78430-fig2-figsupp1-data1.zip › fig 2-figure supplement 1-source data 2/unlabeled fig 2 figure suppl-source data 2-GAPDH.tif]

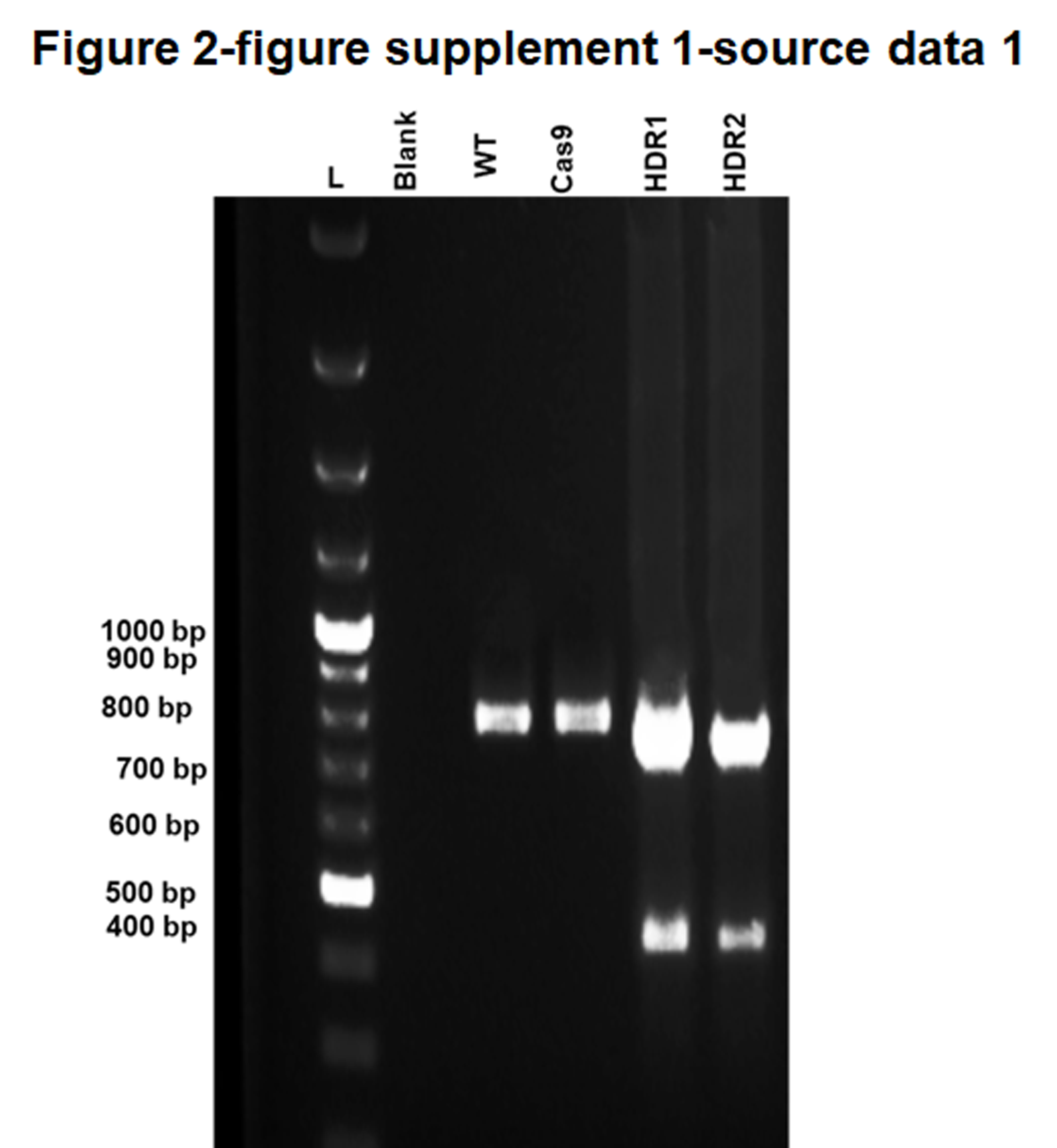

Supplement: Figure 2—figure supplement 1—source data 2. [file elife-78430-fig2-figsupp1-data2.zip › fig 2-figure supplement 1-source data 1/figure 2-figure supplement 1-source data 1.tif]

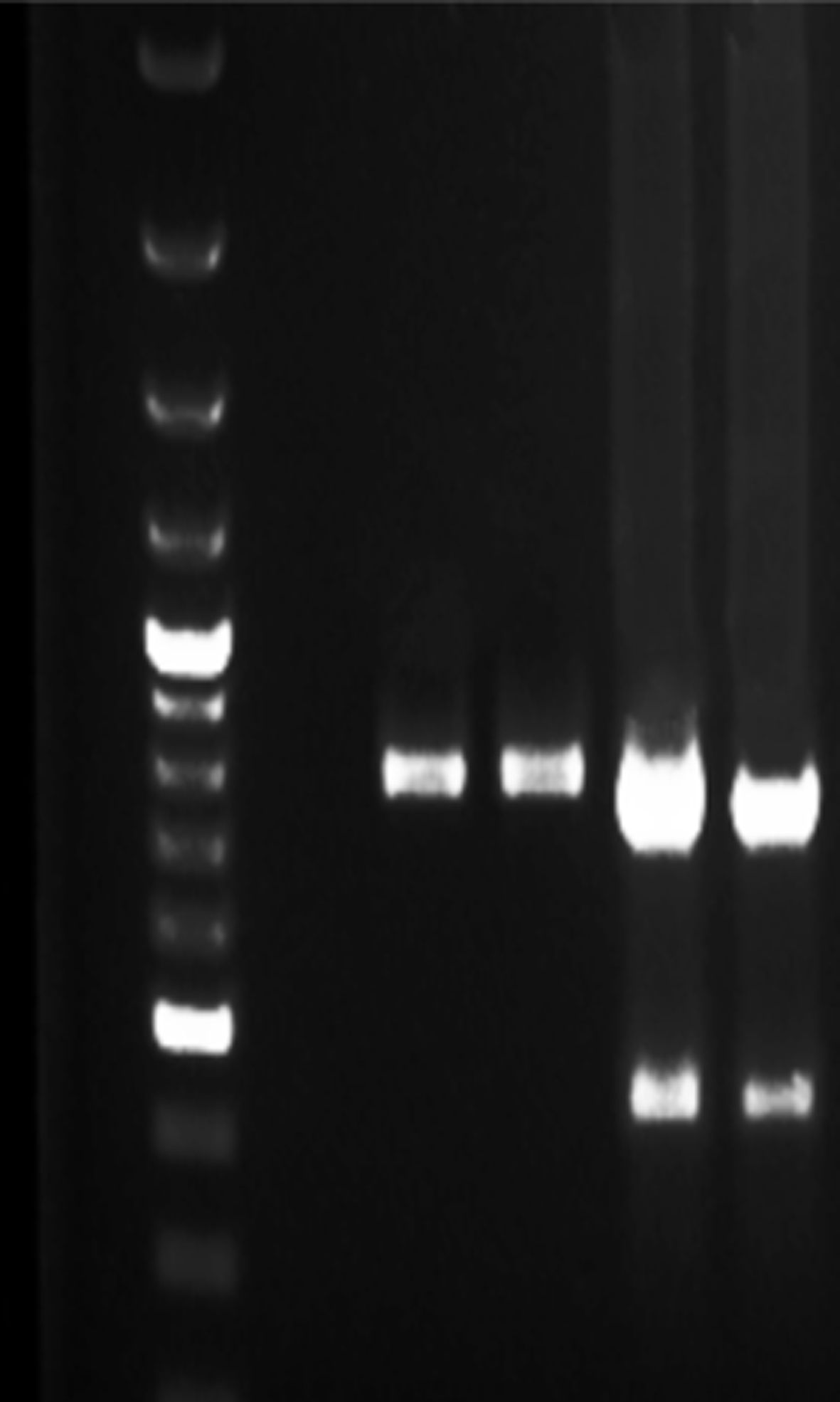

Supplement: Figure 2—figure supplement 1—source data 2. [file elife-78430-fig2-figsupp1-data2.zip › fig 2-figure supplement 1-source data 1/unlabeled fig 2 figure supplement 1-source data 1.tif]

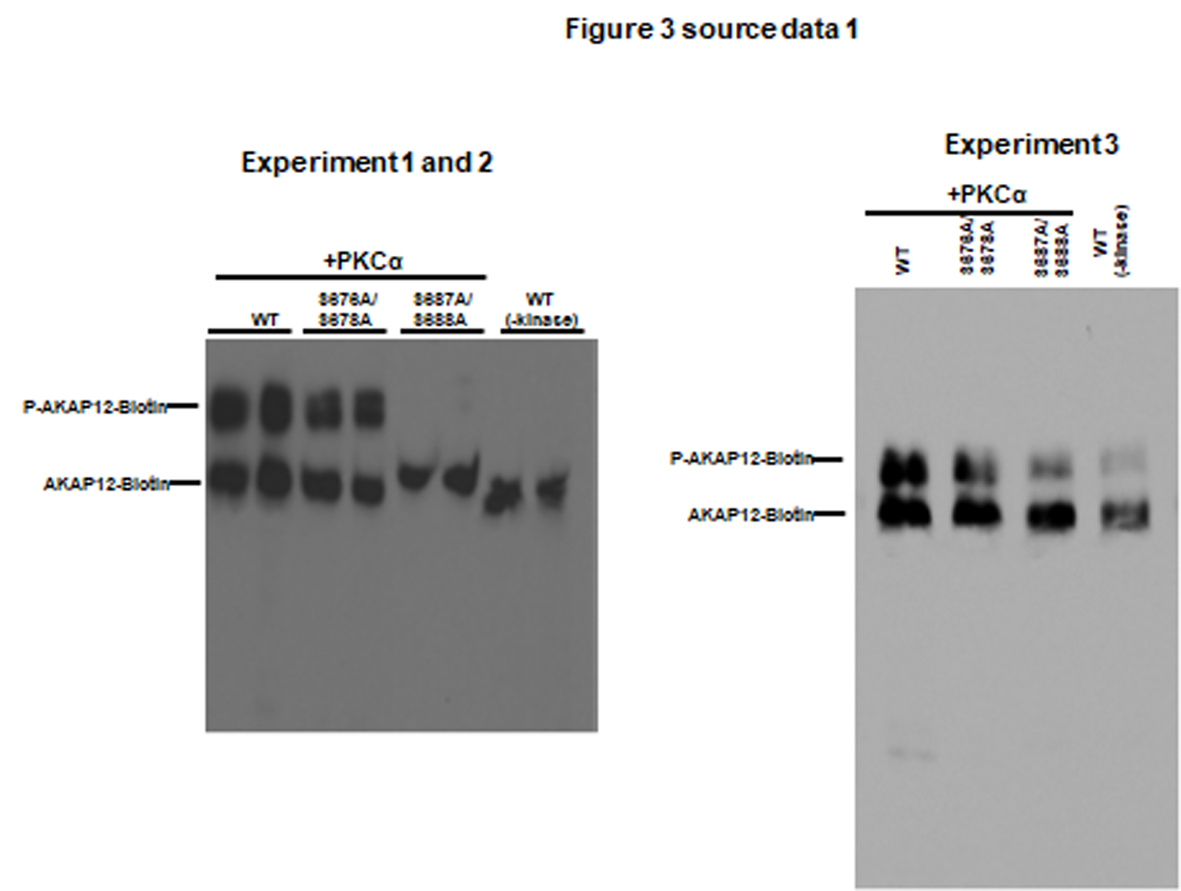

Supplement: Figure 3—source data 1. [file elife-78430-fig3-data1.zip › figure 3-source data 1/figure 3-source data 1.tif]

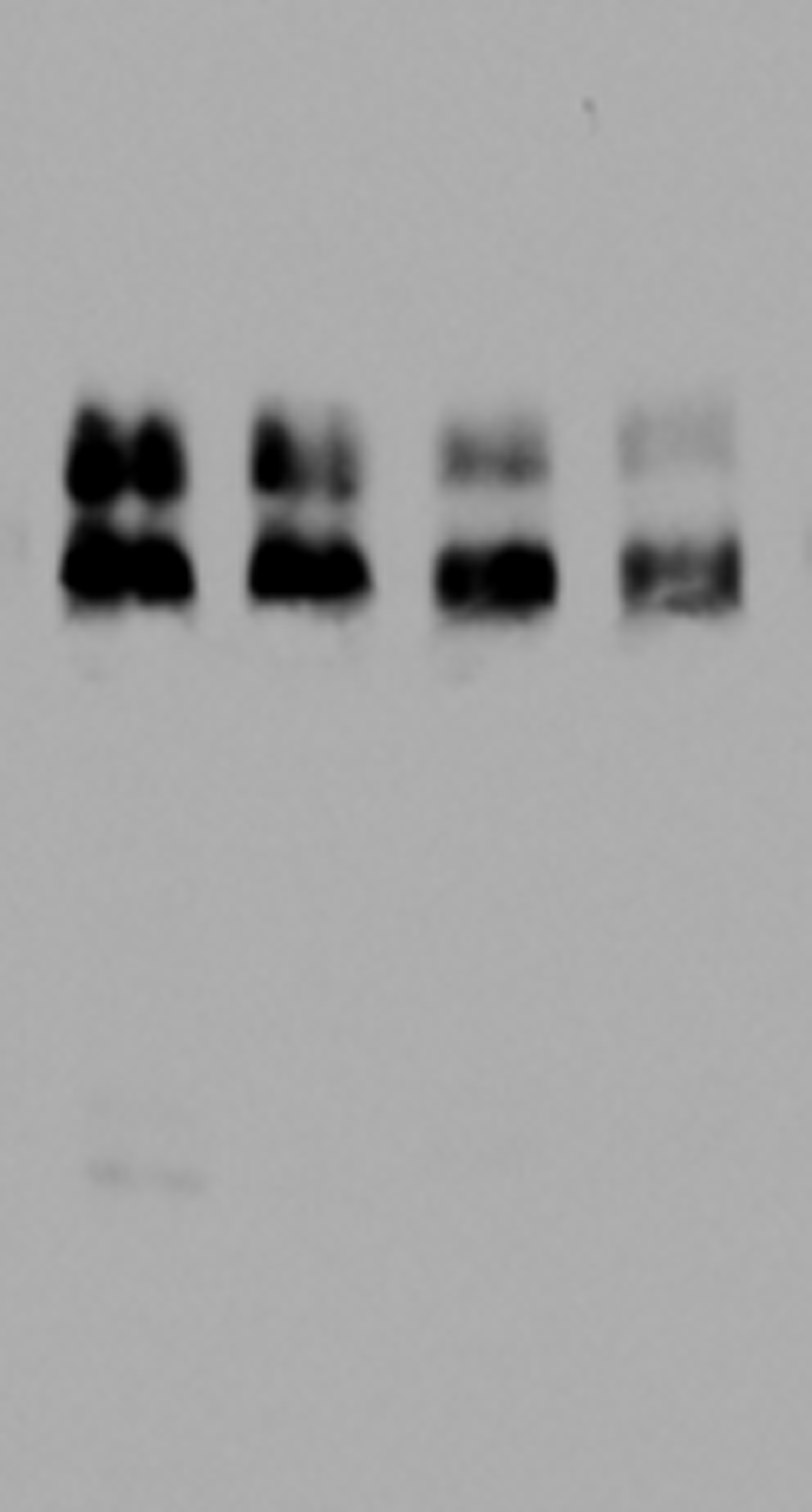

Supplement: Figure 3—source data 1. [file elife-78430-fig3-data1.zip › figure 3-source data 1/unlabeled figure 3-source data 1-exp 3.tif]

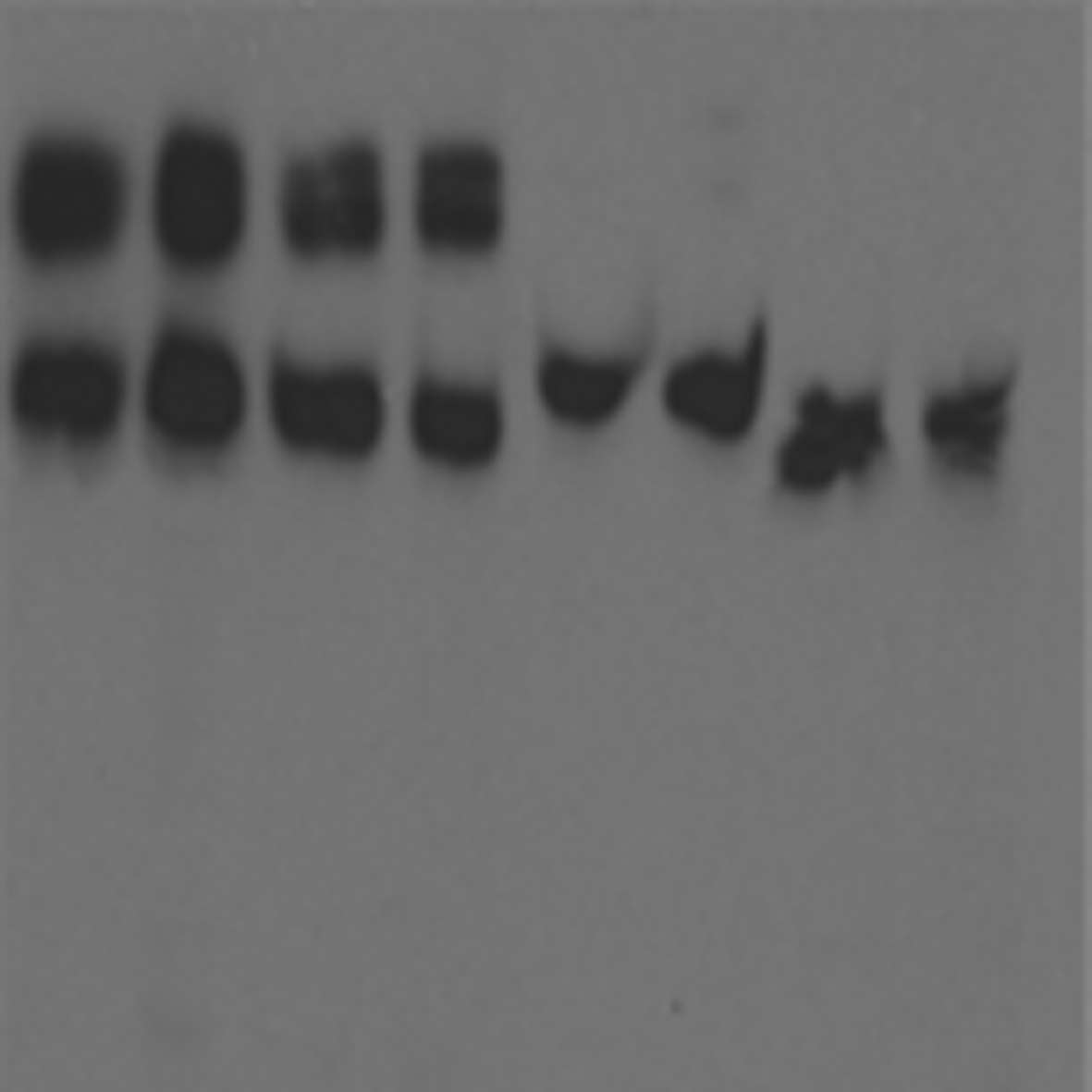

Supplement: Figure 3—source data 1. [file elife-78430-fig3-data1.zip › figure 3-source data 1/unlabeled figure 3-source data 1-exp1 and 2.tif]

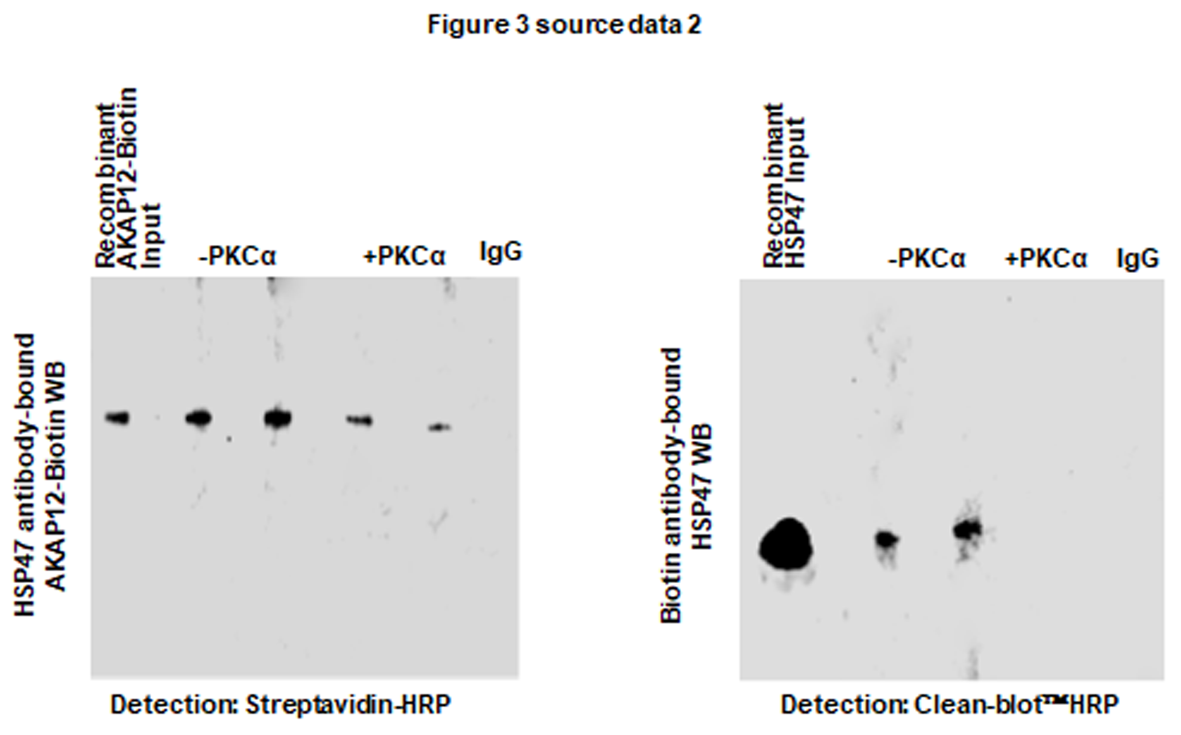

Supplement: Figure 3—source data 2. [file elife-78430-fig3-data2.zip › figure 3-source data 2/Figure 3-source data 2.tif]

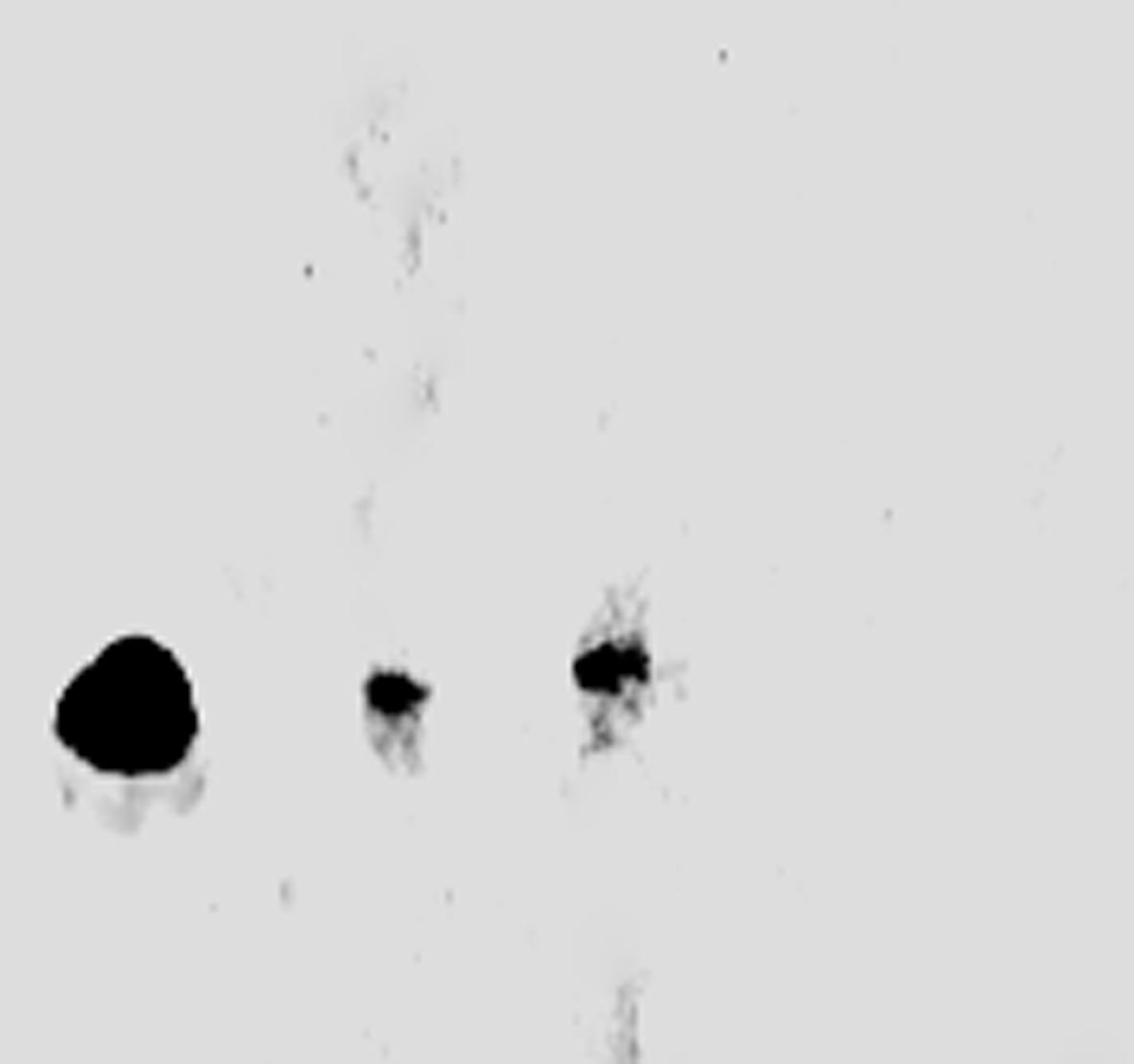

Supplement: Figure 3—source data 2. [file elife-78430-fig3-data2.zip › figure 3-source data 2/unlabeled figure 3-source data 2-AKAP12-Biotin-HSP47.tif]

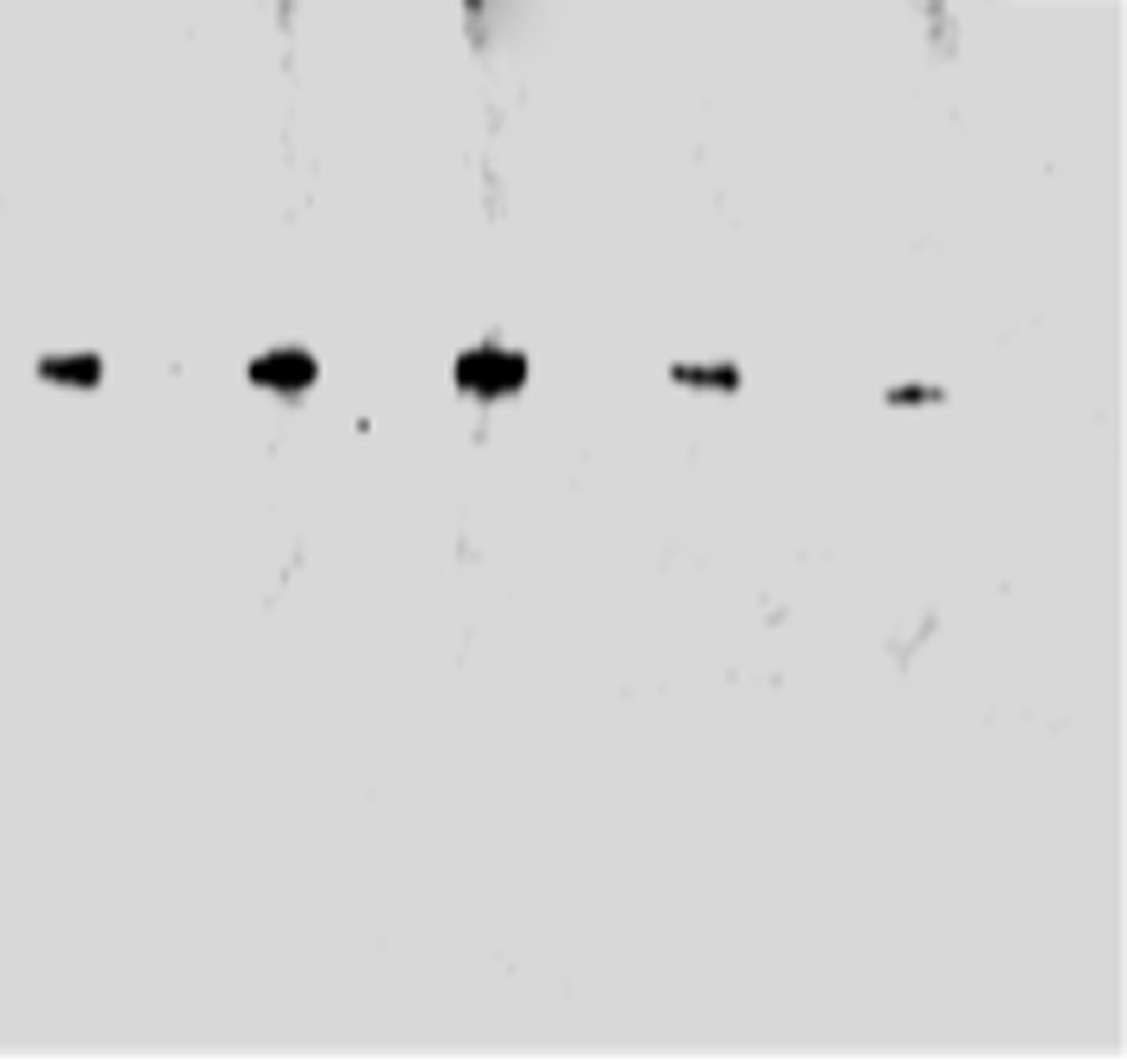

Supplement: Figure 3—source data 2. [file elife-78430-fig3-data2.zip › figure 3-source data 2/unlabeled figure 3-source data 2-HSP47-AKAP12-Biotin.tif]

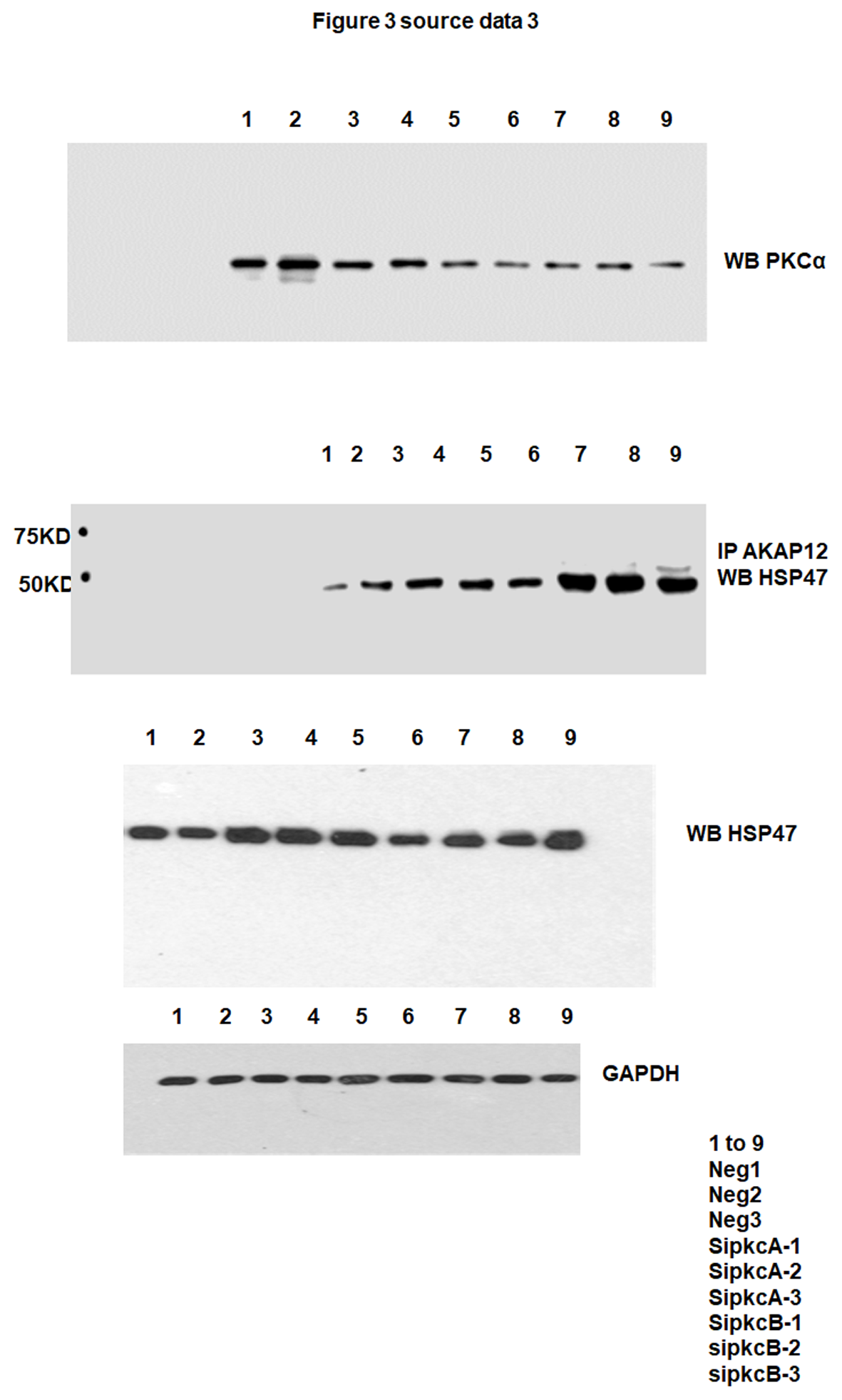

Supplement: Figure 3—source data 3. [file elife-78430-fig3-data3.zip › figure 3-source data 3/Figure 3-source data 3.tif]

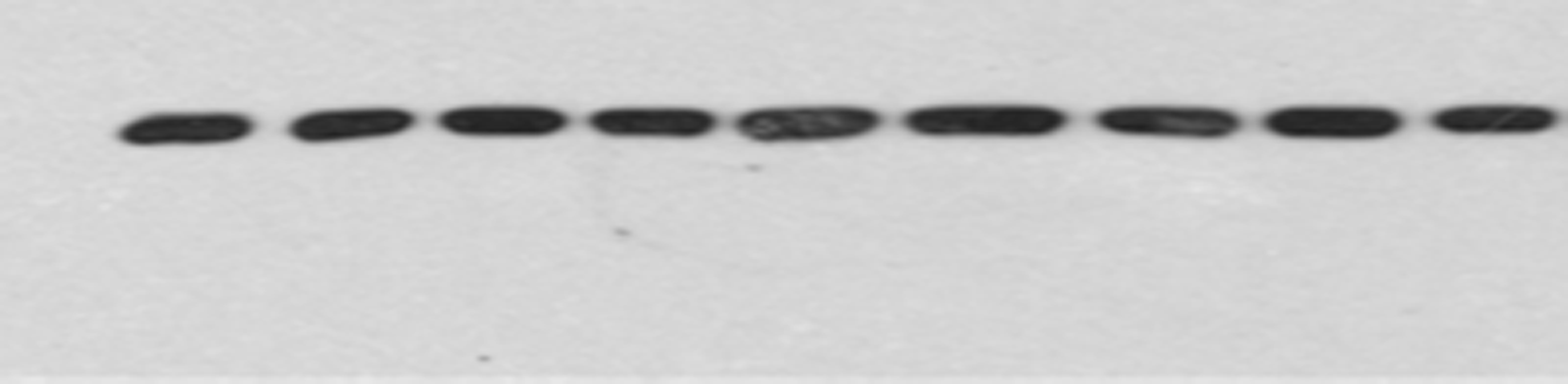

Supplement: Figure 3—source data 3. [file elife-78430-fig3-data3.zip › figure 3-source data 3/unlabeled figure 3-source data 3-GAPDH.tif]

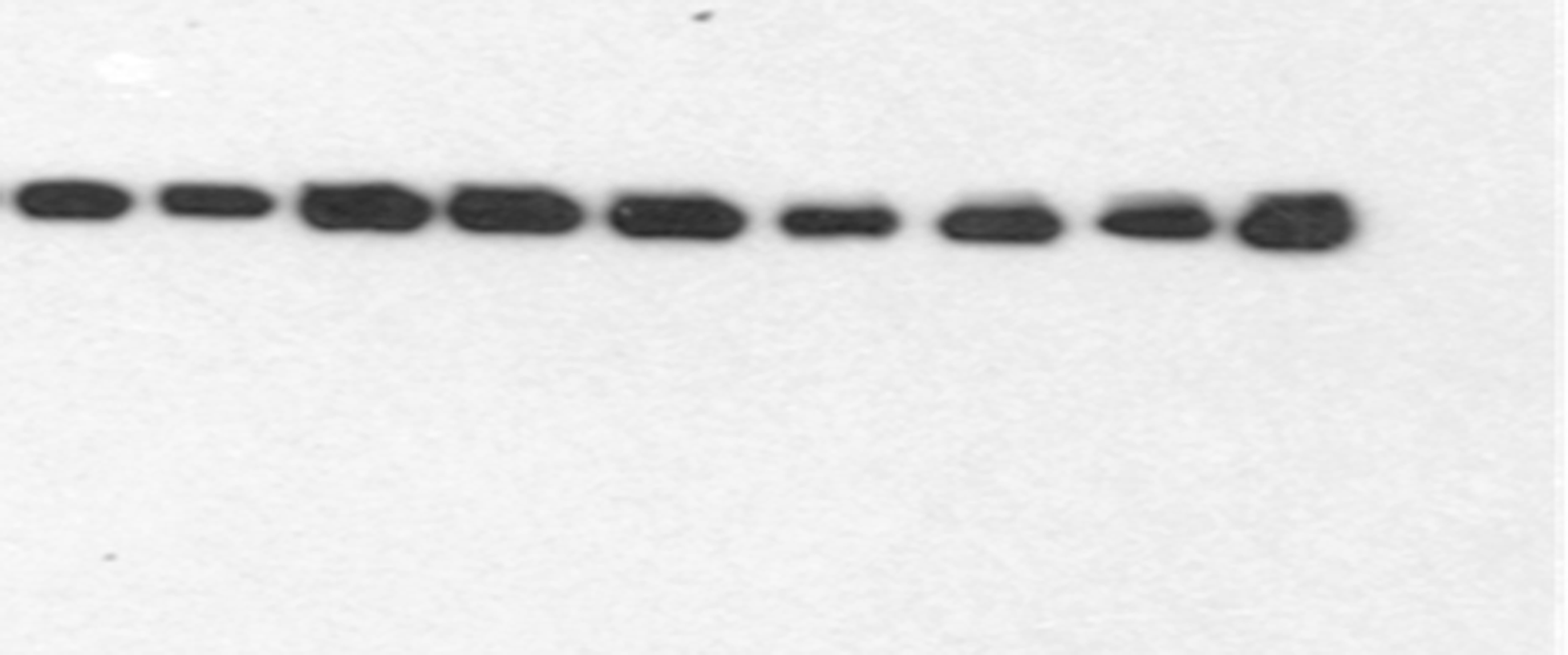

Supplement: Figure 3—source data 3. [file elife-78430-fig3-data3.zip › figure 3-source data 3/unlabeled figure 3-source data 3-HSP47.tif]

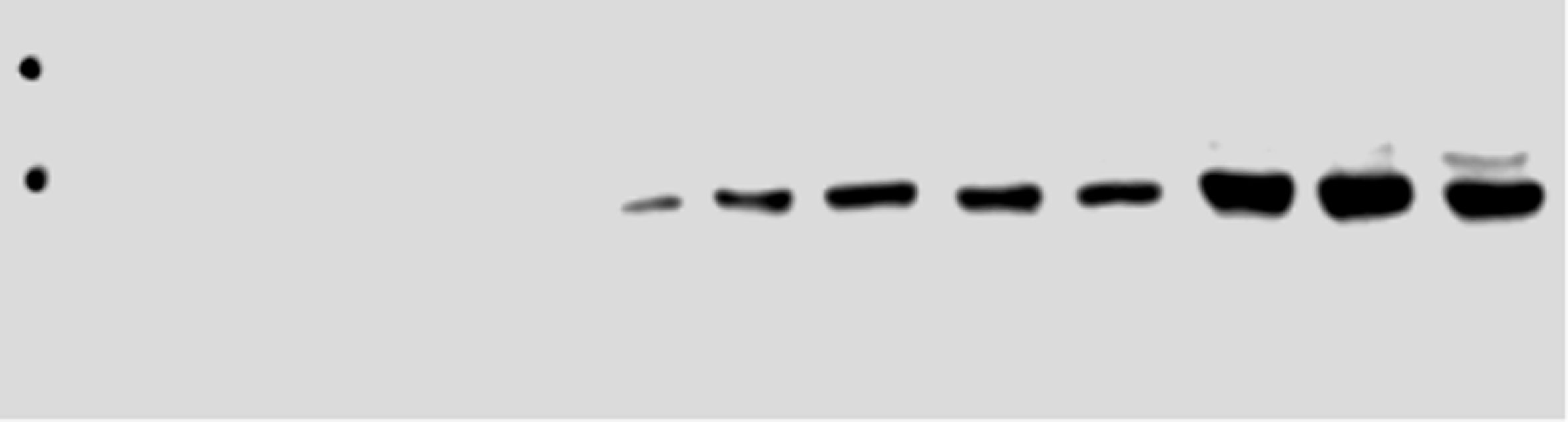

Supplement: Figure 3—source data 3. [file elife-78430-fig3-data3.zip › figure 3-source data 3/unlabeled figure 3-source data 3-IP AKAP12 wb HSP47.tif]

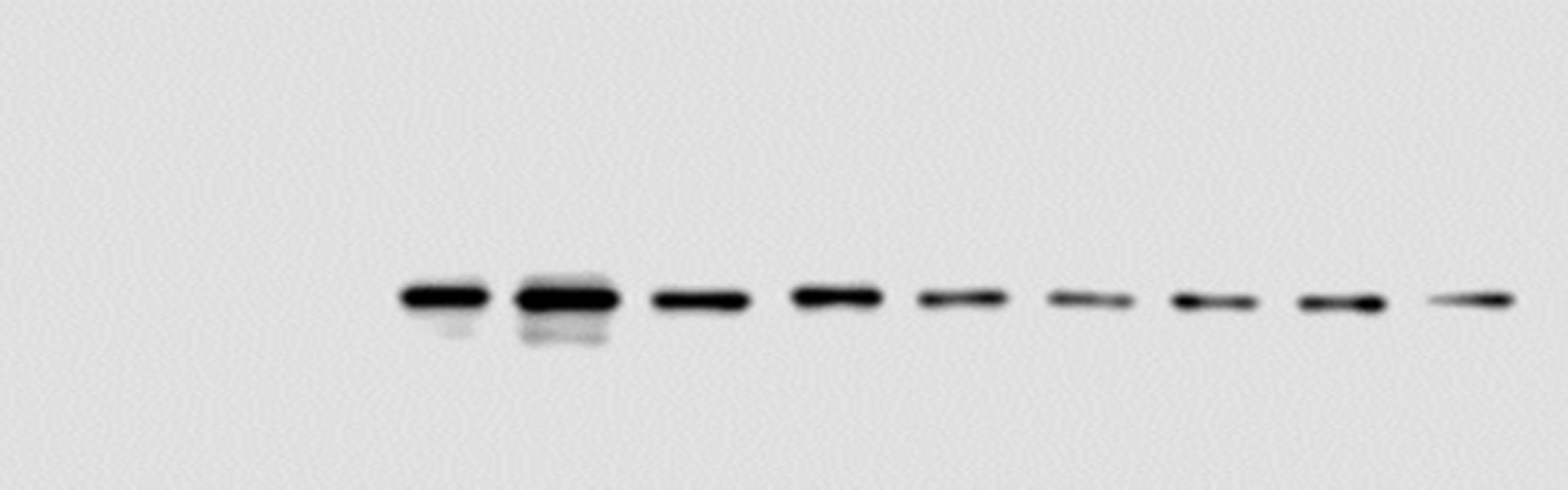

Supplement: Figure 3—source data 3. [file elife-78430-fig3-data3.zip › figure 3-source data 3/unlabeled figure 3-source data 3-PKC alpha.tif]

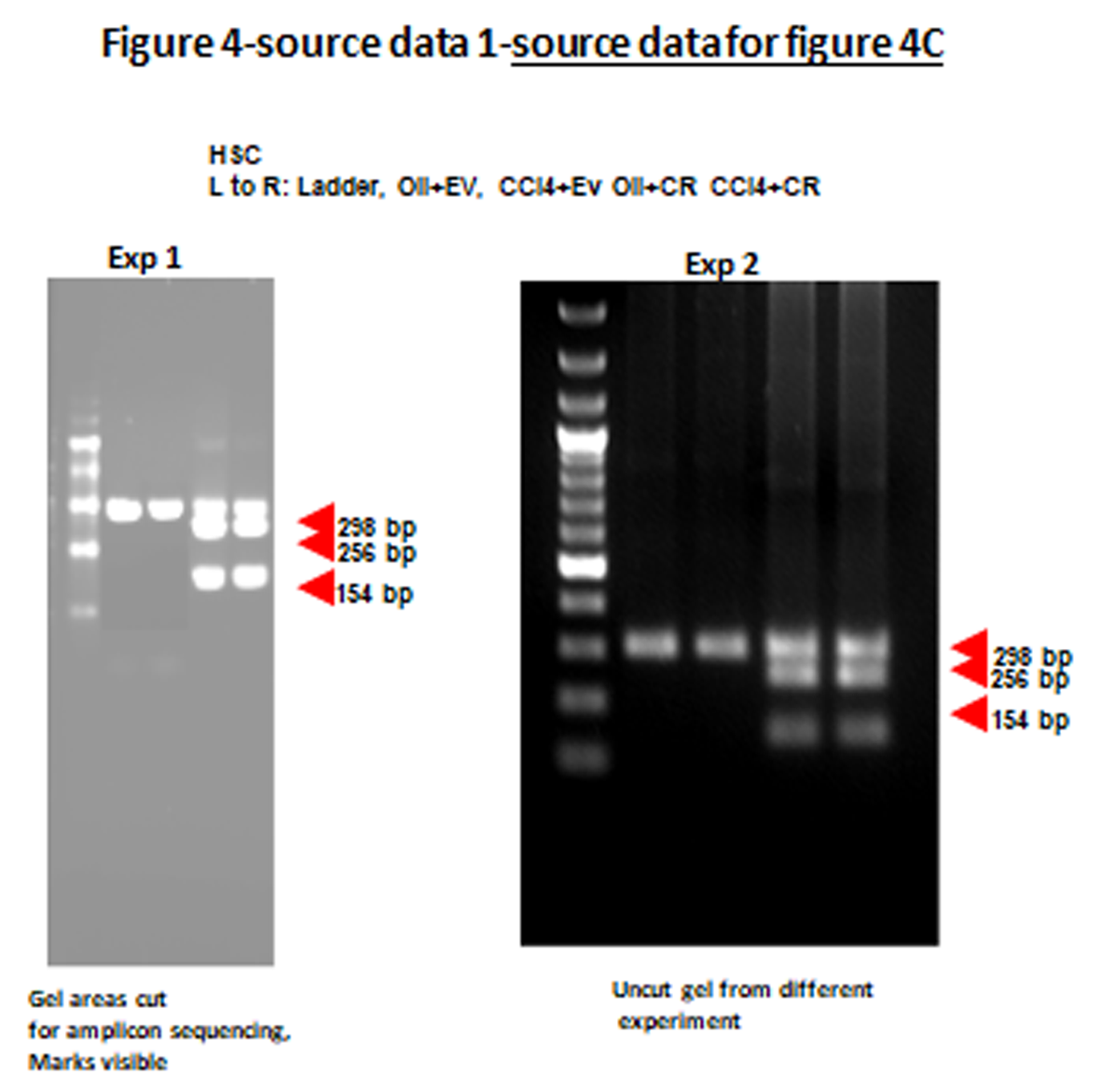

Supplement: Figure 4—source data 1. [file elife-78430-fig4-data1.zip › figure 4-source data 1/figure 4-source data 1.tif]

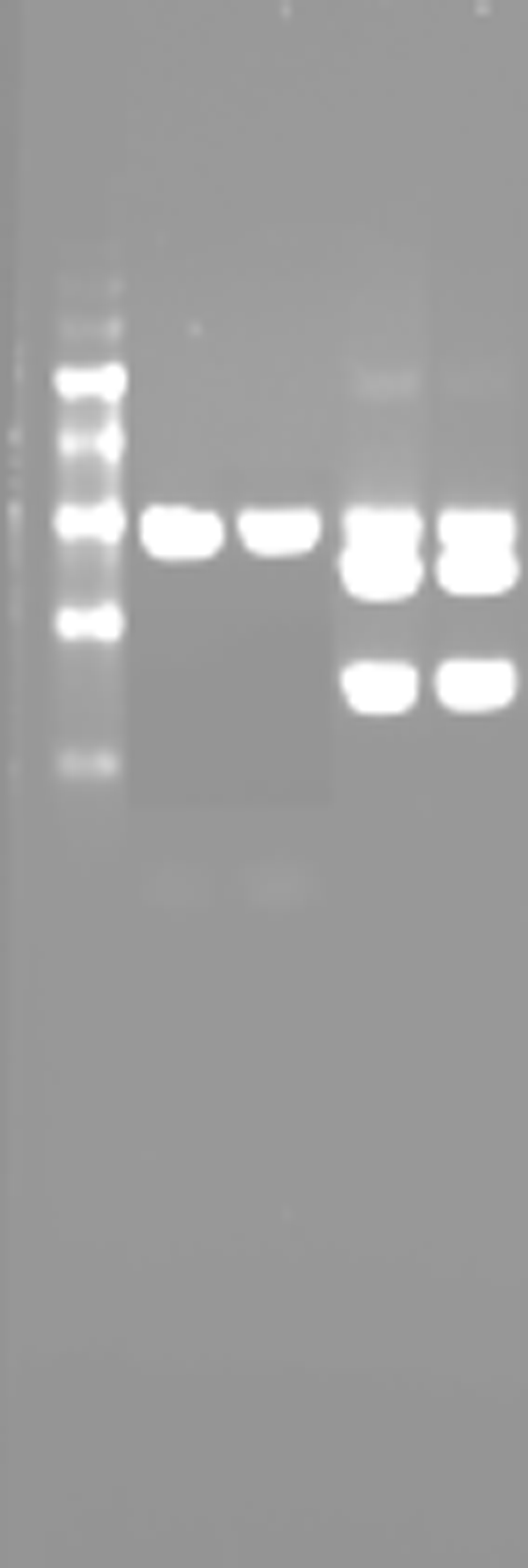

Supplement: Figure 4—source data 1. [file elife-78430-fig4-data1.zip › figure 4-source data 1/unlabeled figure 4-source data 1-exp 1.tif]

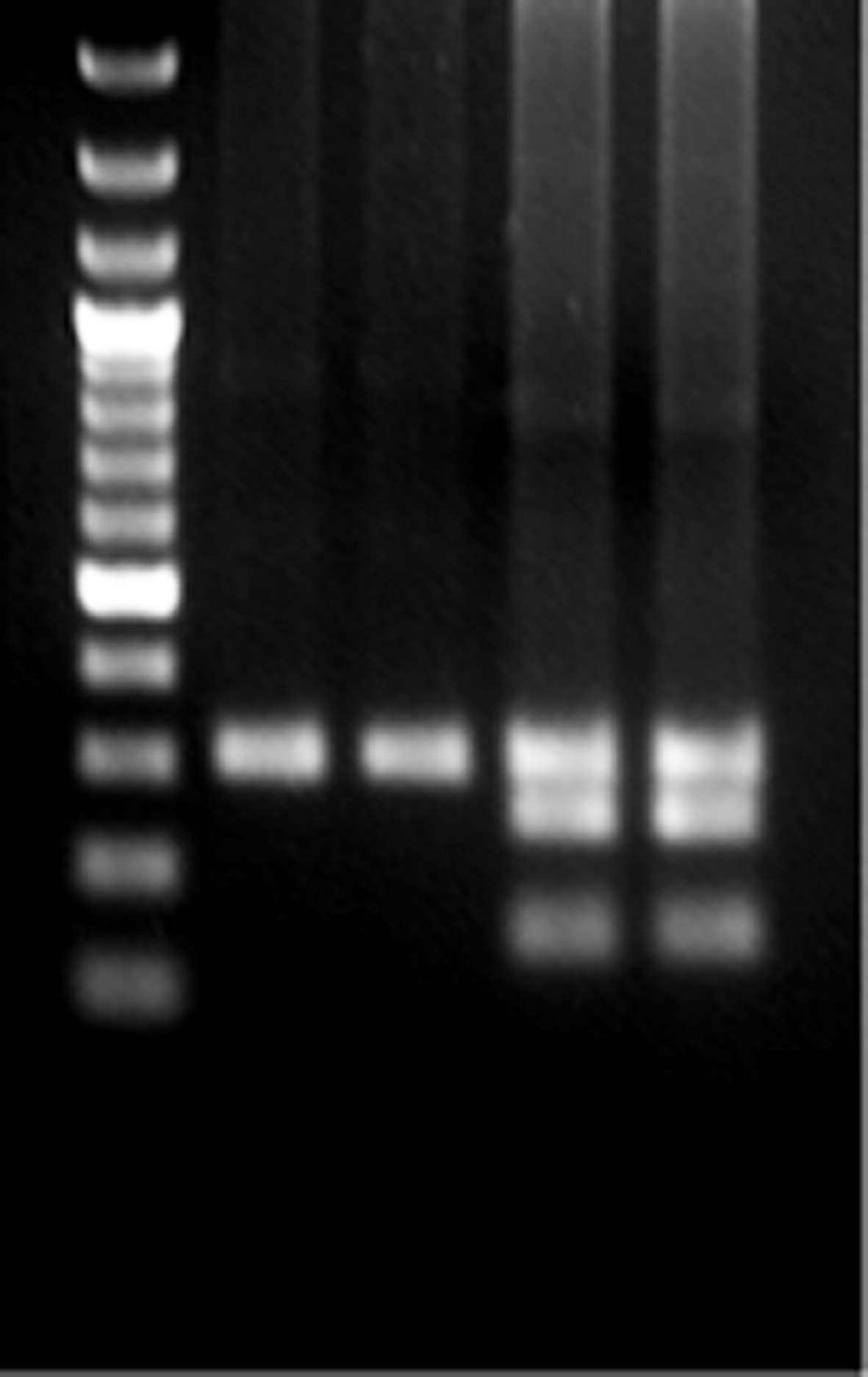

Supplement: Figure 4—source data 1. [file elife-78430-fig4-data1.zip › figure 4-source data 1/unlabeled figure 4-source data 1-exp 2.tif]

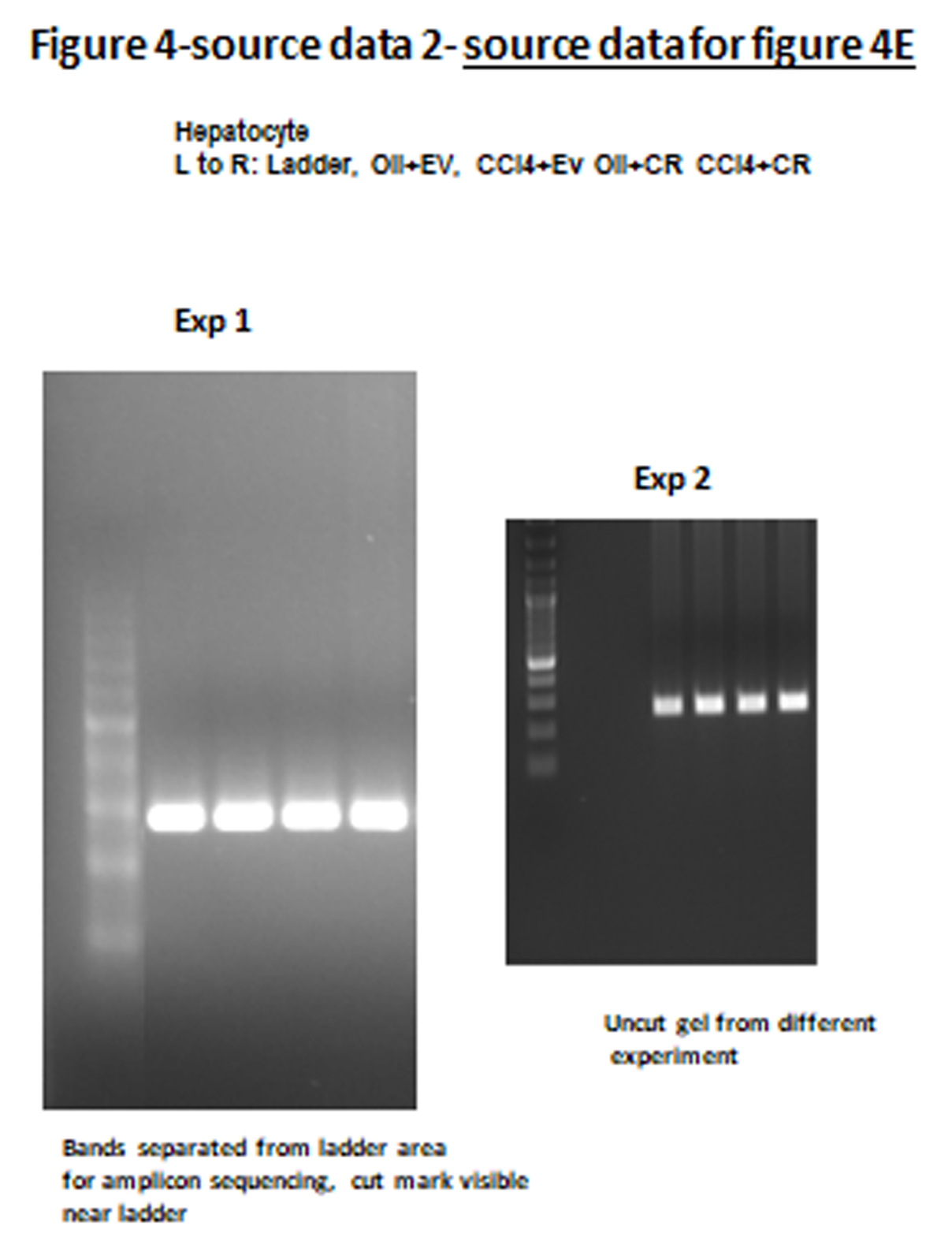

Supplement: Figure 4—source data 2. [file elife-78430-fig4-data2.zip › figure 4-source data 2/figure 4-source data 2.tif]

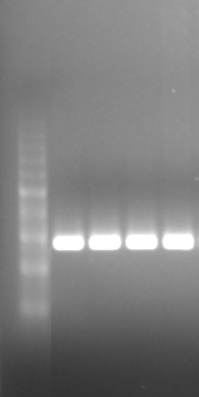

Supplement: Figure 4—source data 2. [file elife-78430-fig4-data2.zip › figure 4-source data 2/unlabeled figure 4-source data 2-exp 1.tif]

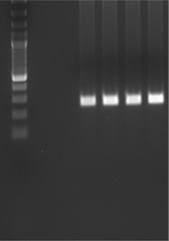

Supplement: Figure 4—source data 2. [file elife-78430-fig4-data2.zip › figure 4-source data 2/unlabeled figure 4-source data 2-exp 2.tif]

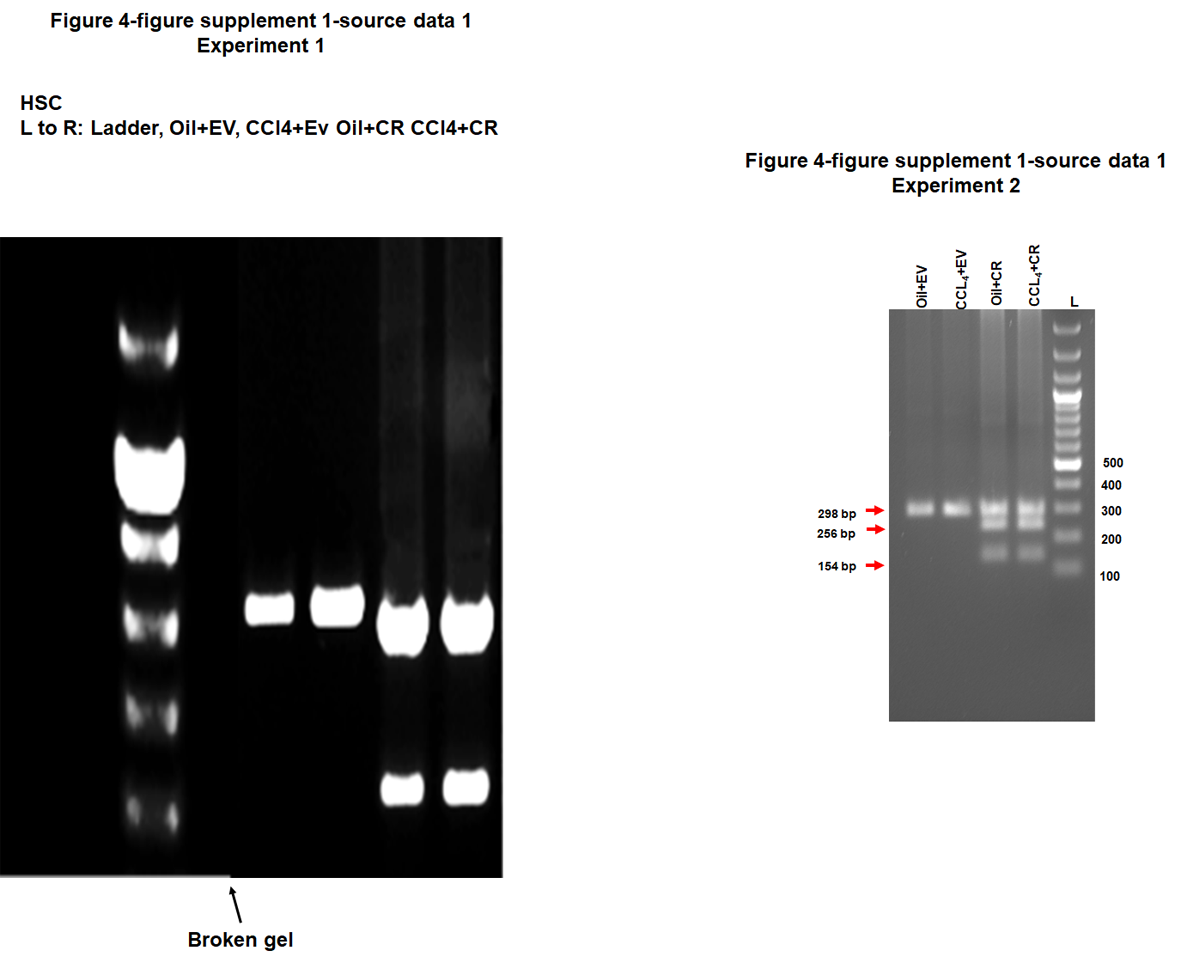

Supplement: Figure 4—figure supplement 1—source data 1. [file elife-78430-fig4-figsupp1-data1.zip › fig 4-figure supplement 1-source data 1/figure 4-figure supplement 1-source data 1.tif]

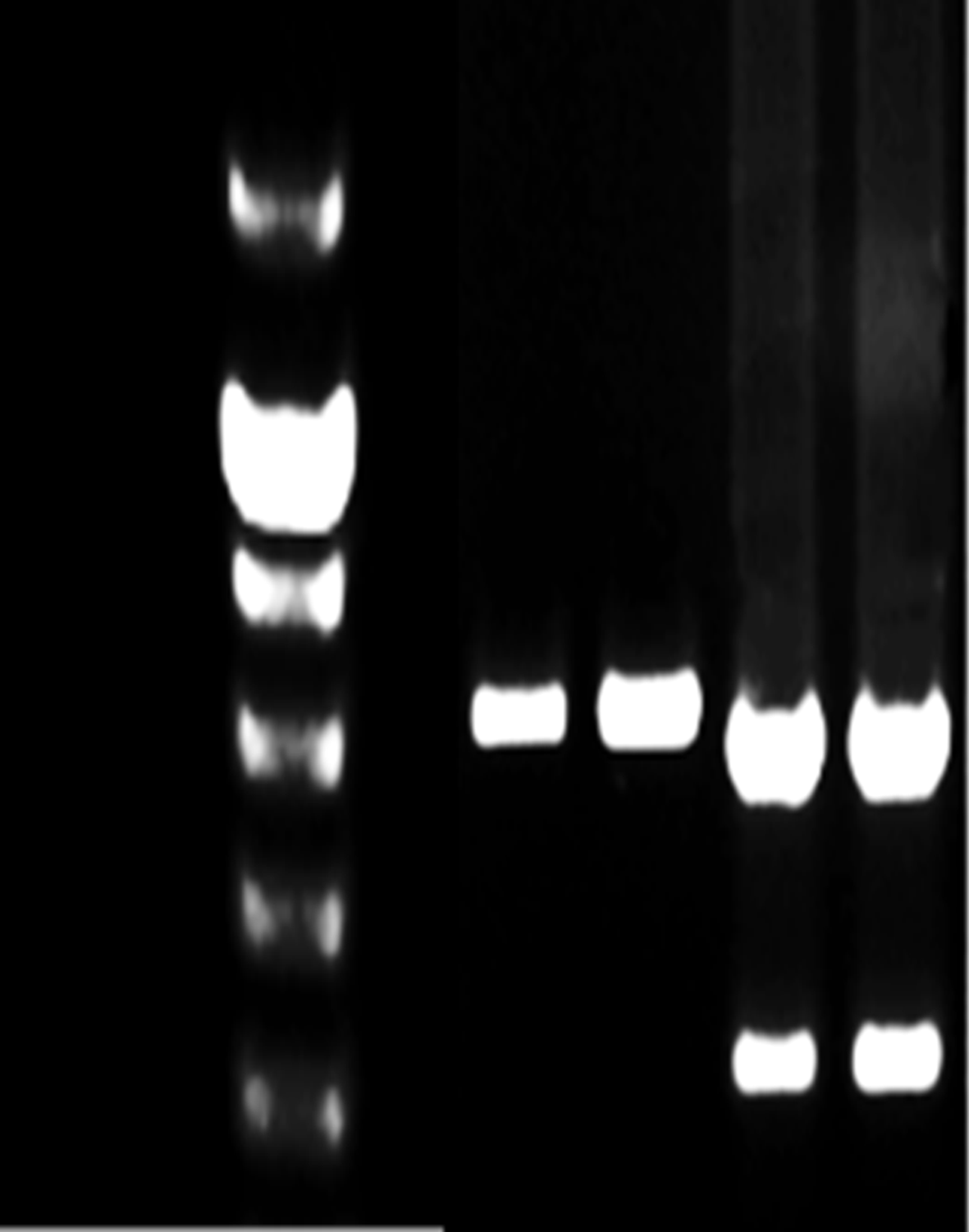

Supplement: Figure 4—figure supplement 1—source data 1. [file elife-78430-fig4-figsupp1-data1.zip › fig 4-figure supplement 1-source data 1/unlabeled fig-fig suppl 1-source data 1-exp1.tif]

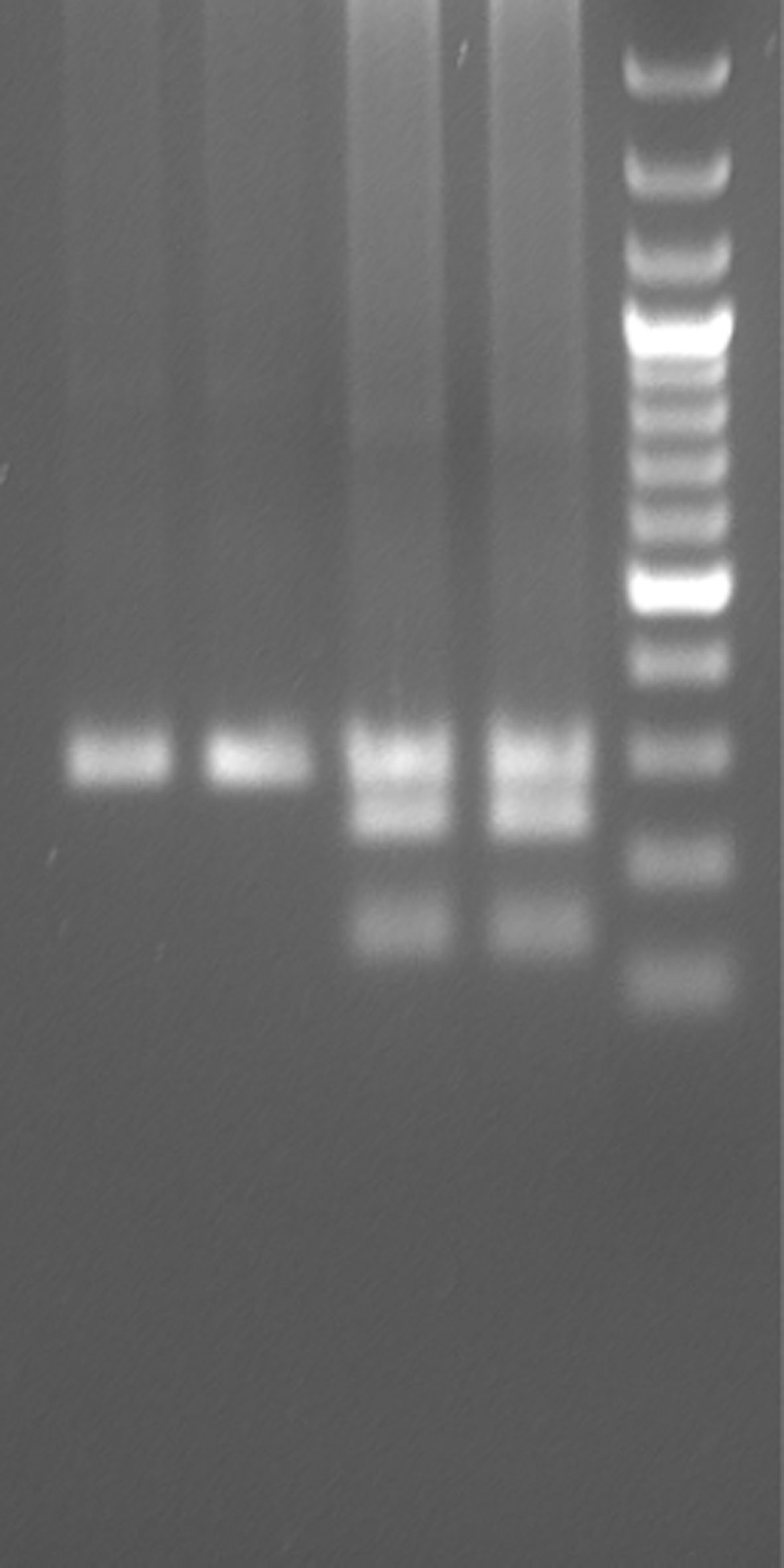

Supplement: Figure 4—figure supplement 1—source data 1. [file elife-78430-fig4-figsupp1-data1.zip › fig 4-figure supplement 1-source data 1/unlabeled fig-fig suppl 1-source data 1-exp2.tif]

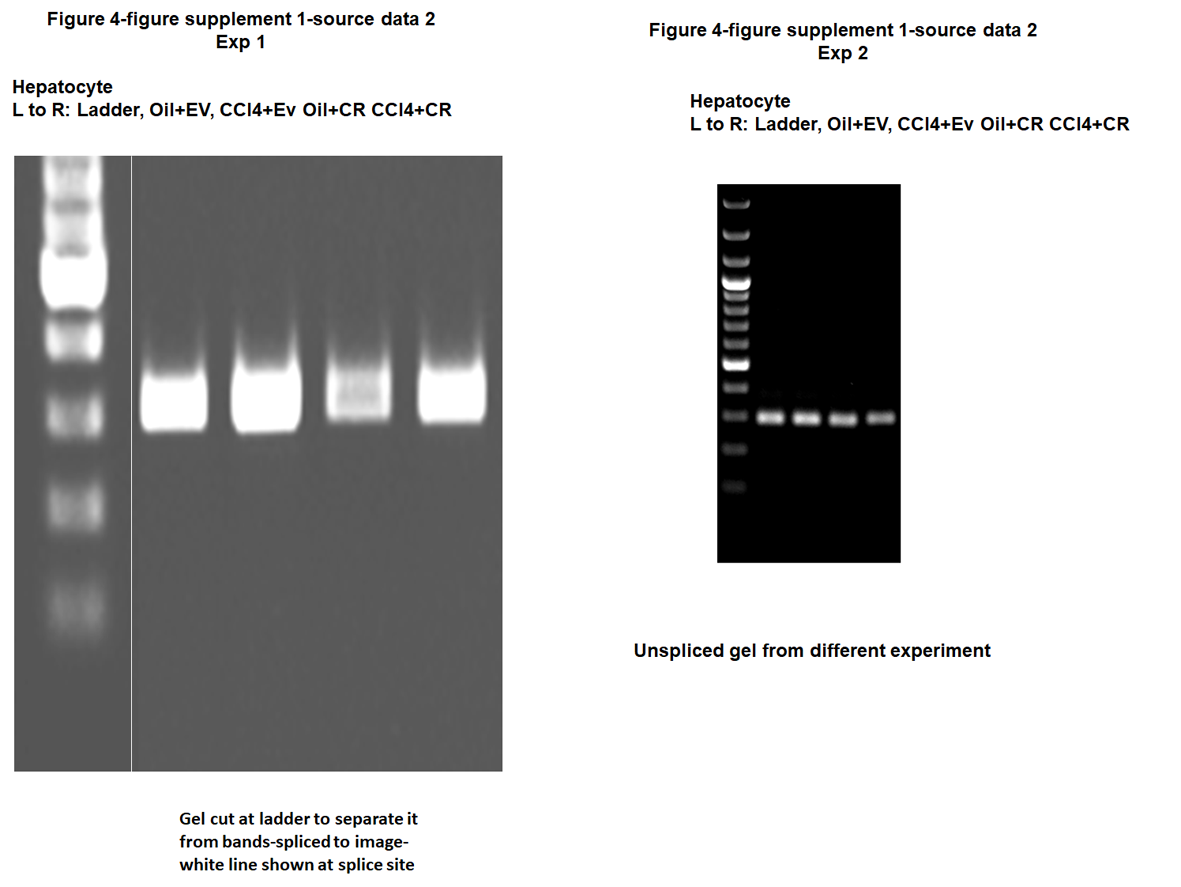

Supplement: Figure 4—figure supplement 1—source data 2. [file elife-78430-fig4-figsupp1-data2.zip › fig 4-figure supplement 1-source data 2/Figure 4-figure supplement 1-source data 2.tif]

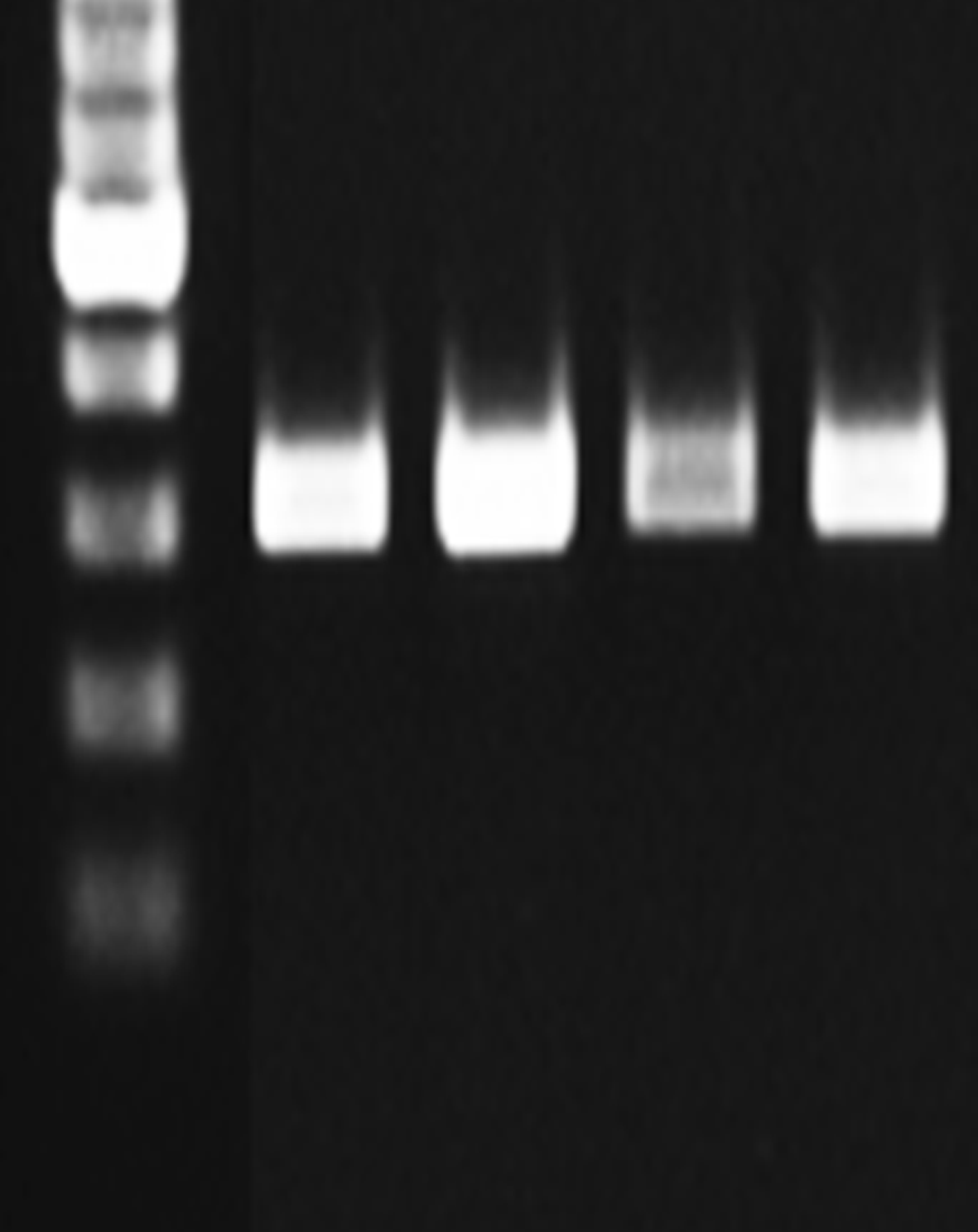

Supplement: Figure 4—figure supplement 1—source data 2. [file elife-78430-fig4-figsupp1-data2.zip › fig 4-figure supplement 1-source data 2/unlabeled fig 4-fig suppl 1-source data 2-exp 1.tif]

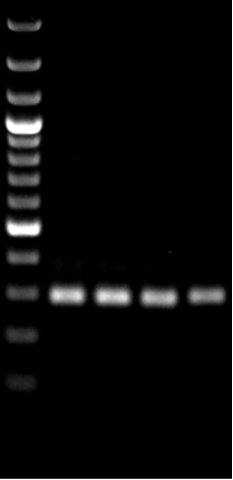

Supplement: Figure 4—figure supplement 1—source data 2. [file elife-78430-fig4-figsupp1-data2.zip › fig 4-figure supplement 1-source data 2/unlabeled fig 4-fig suppl 1-source data 2-exp 2.tif]

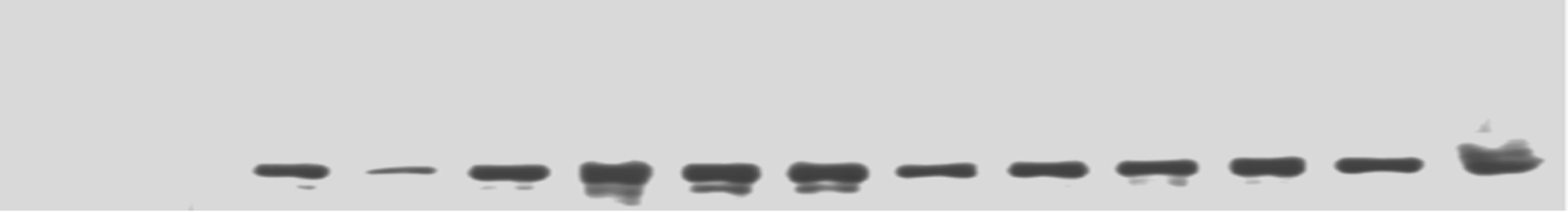

Supplement: Figure 6—source data 1. [file elife-78430-fig6-data1.zip › fig 6-source data 1/unlabeled figure 6-source data 1-AKAP12.tif]

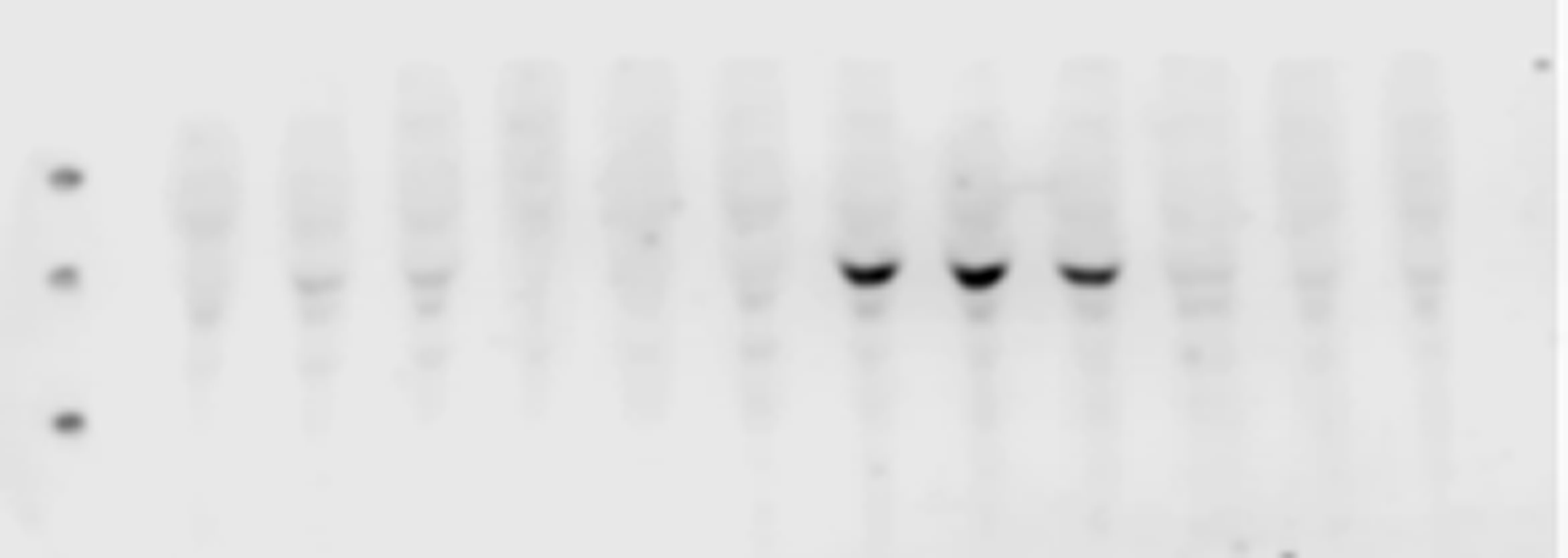

Supplement: Figure 6—source data 1. [file elife-78430-fig6-data1.zip › fig 6-source data 1/unlabeled figure 6-source data 1-alpha-SMA.tif]

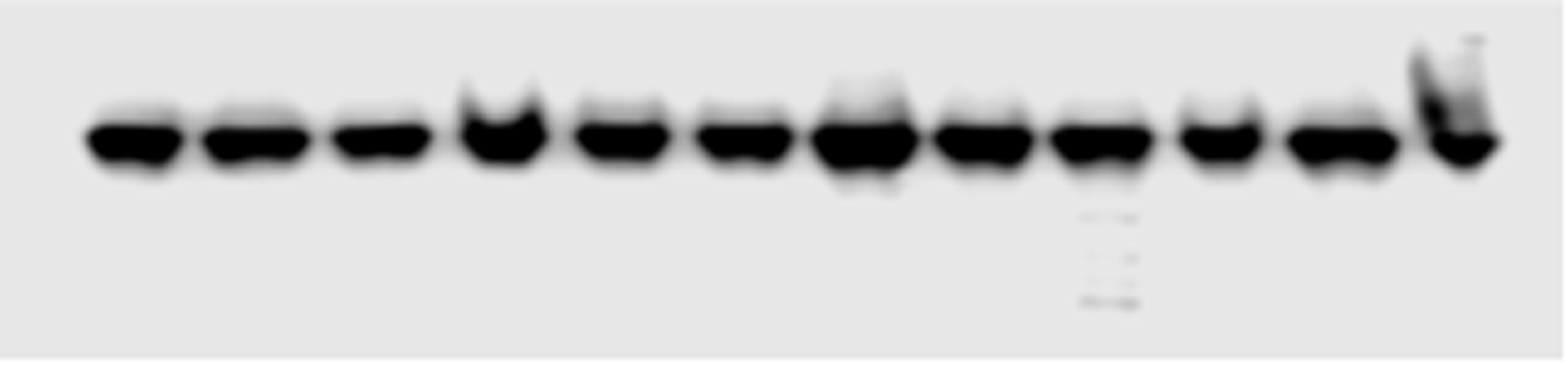

Supplement: Figure 6—source data 1. [file elife-78430-fig6-data1.zip › fig 6-source data 1/unlabeled figure 6-source data 1-GAPDH.tif]

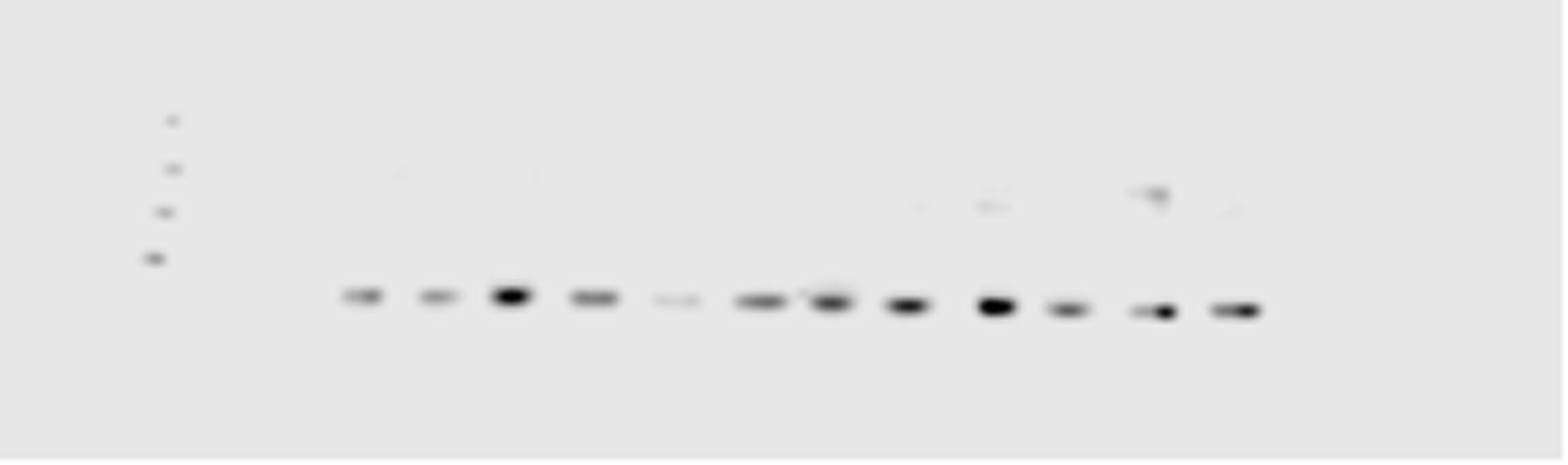

Supplement: Figure 6—source data 1. [file elife-78430-fig6-data1.zip › fig 6-source data 1/unlabeled figure 6-source data 1-hsp47.tif]

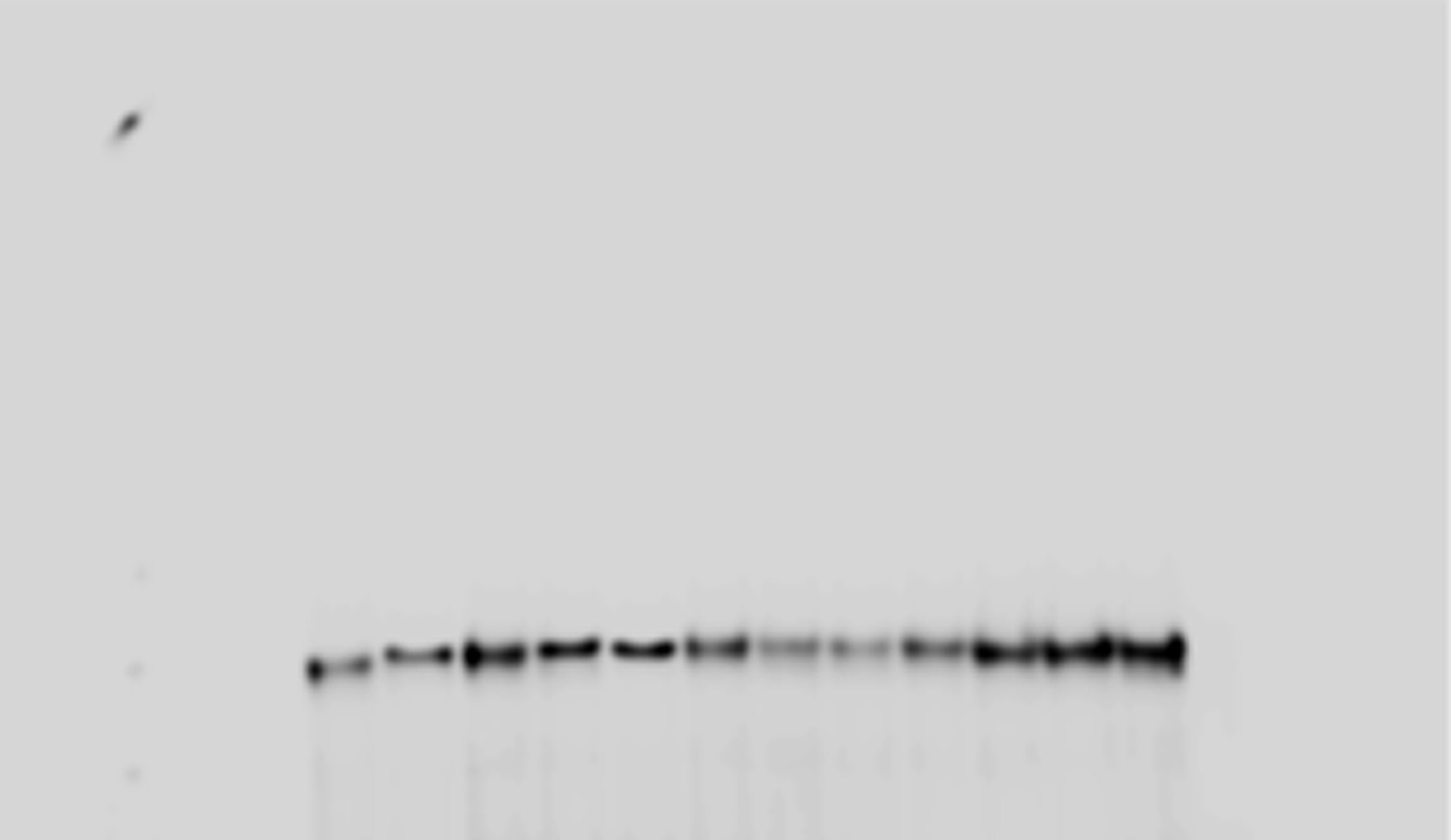

Supplement: Figure 6—source data 1. [file elife-78430-fig6-data1.zip › fig 6-source data 1/unlabeled figure 6-source data 1-IP AKAP12 wb hsp47.tif]

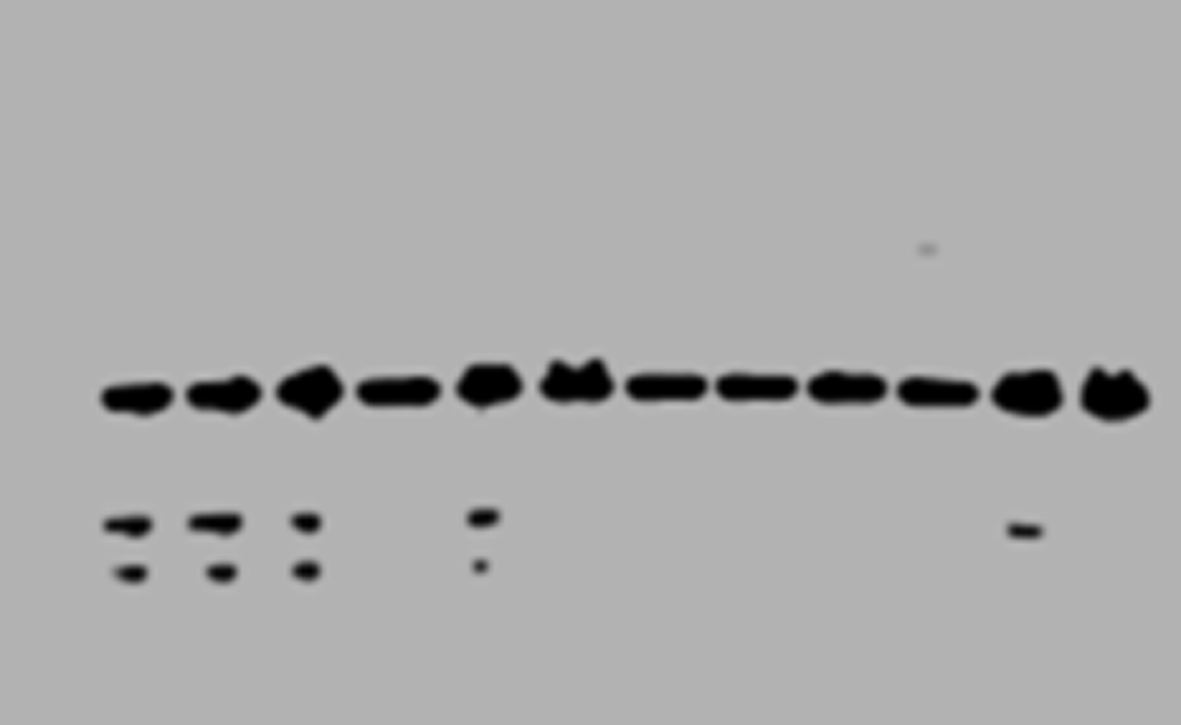

Supplement: Figure 6—source data 2. [file elife-78430-fig6-data2.zip › fig 6-source data 2/unlabeled fig 6-source data 2-AKAP12 additional.tif]

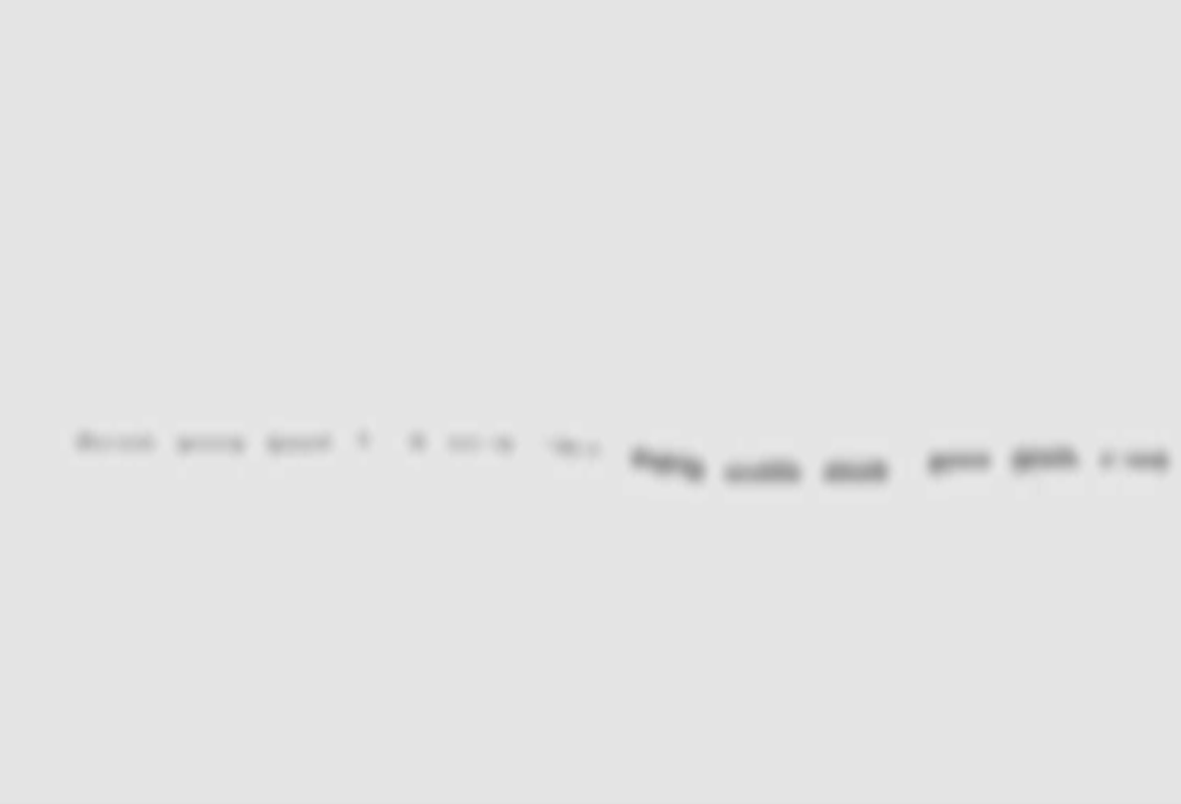

Supplement: Figure 6—source data 2. [file elife-78430-fig6-data2.zip › fig 6-source data 2/unlabeled fig 6-source data 2-alpha-SMA additional.tif]

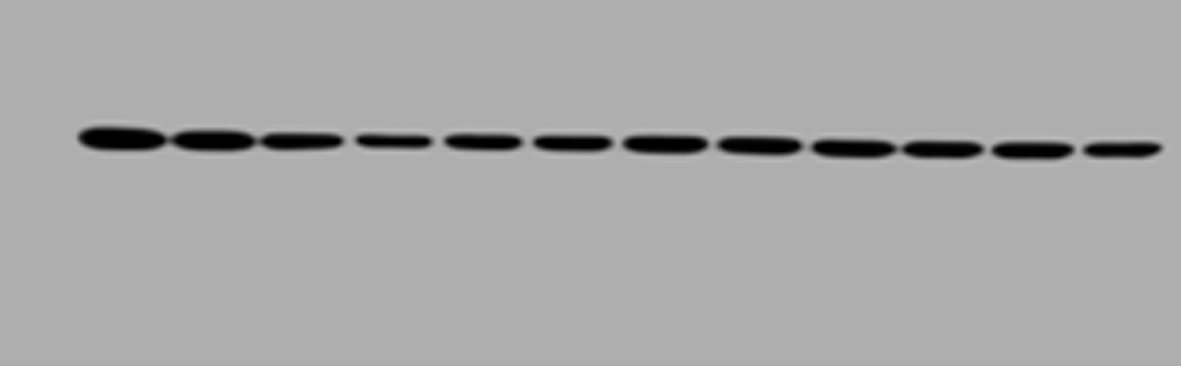

Supplement: Figure 6—source data 2. [file elife-78430-fig6-data2.zip › fig 6-source data 2/unlabeled fig 6-source data 2-GAPDH additional.tif]

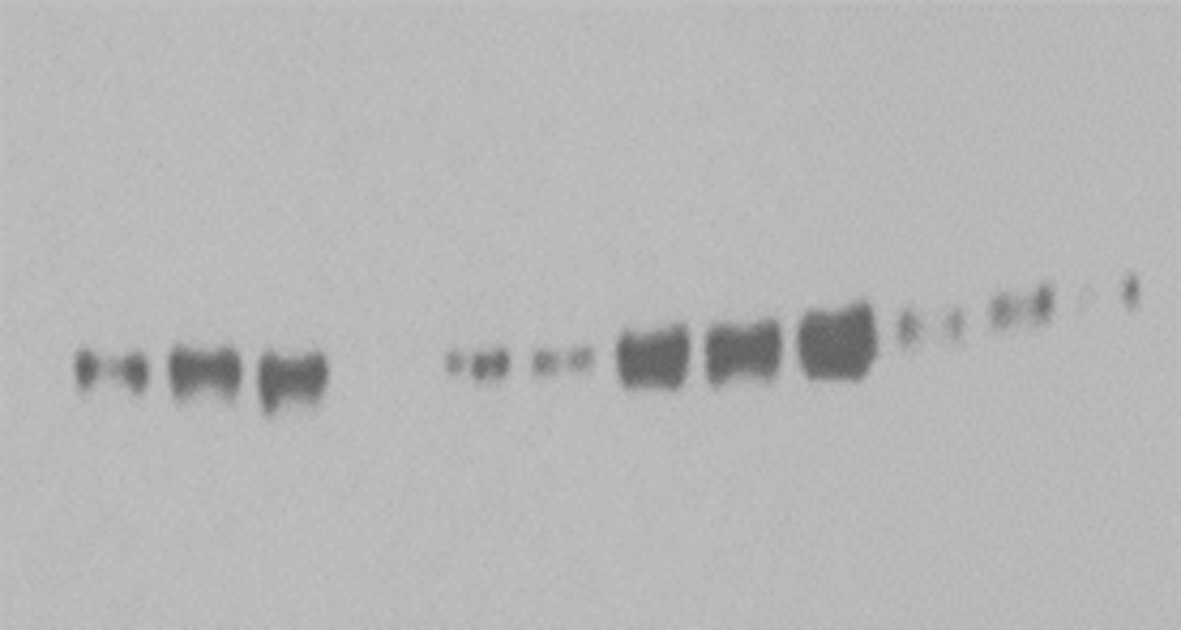

Supplement: Figure 6—source data 2. [file elife-78430-fig6-data2.zip › fig 6-source data 2/unlabeled fig 6-source data 2-hsp47 additional.tif]

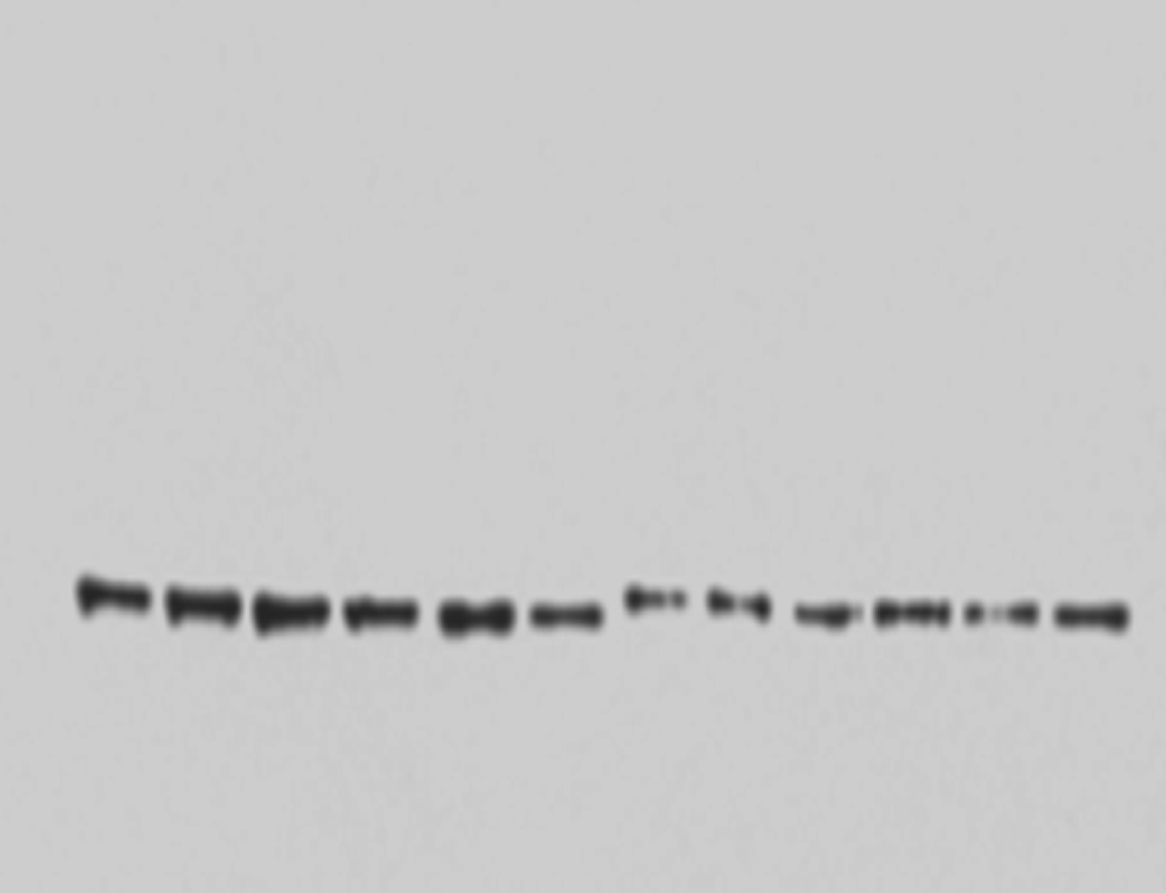

Supplement: Figure 6—source data 2. [file elife-78430-fig6-data2.zip › fig 6-source data 2/unlabeled fig 6-source data 2-IP AKAP12 wb hsp47-additional.tif]

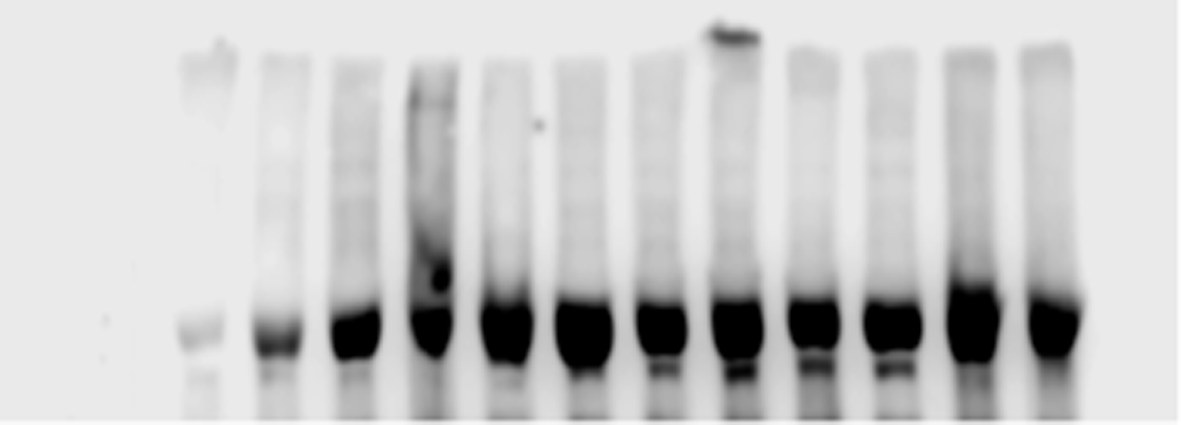

Supplement: Figure 6—source data 2. [file elife-78430-fig6-data2.zip › fig 6-source data 2/unlabeled figure 6-source data 2-AKAP12.tif]

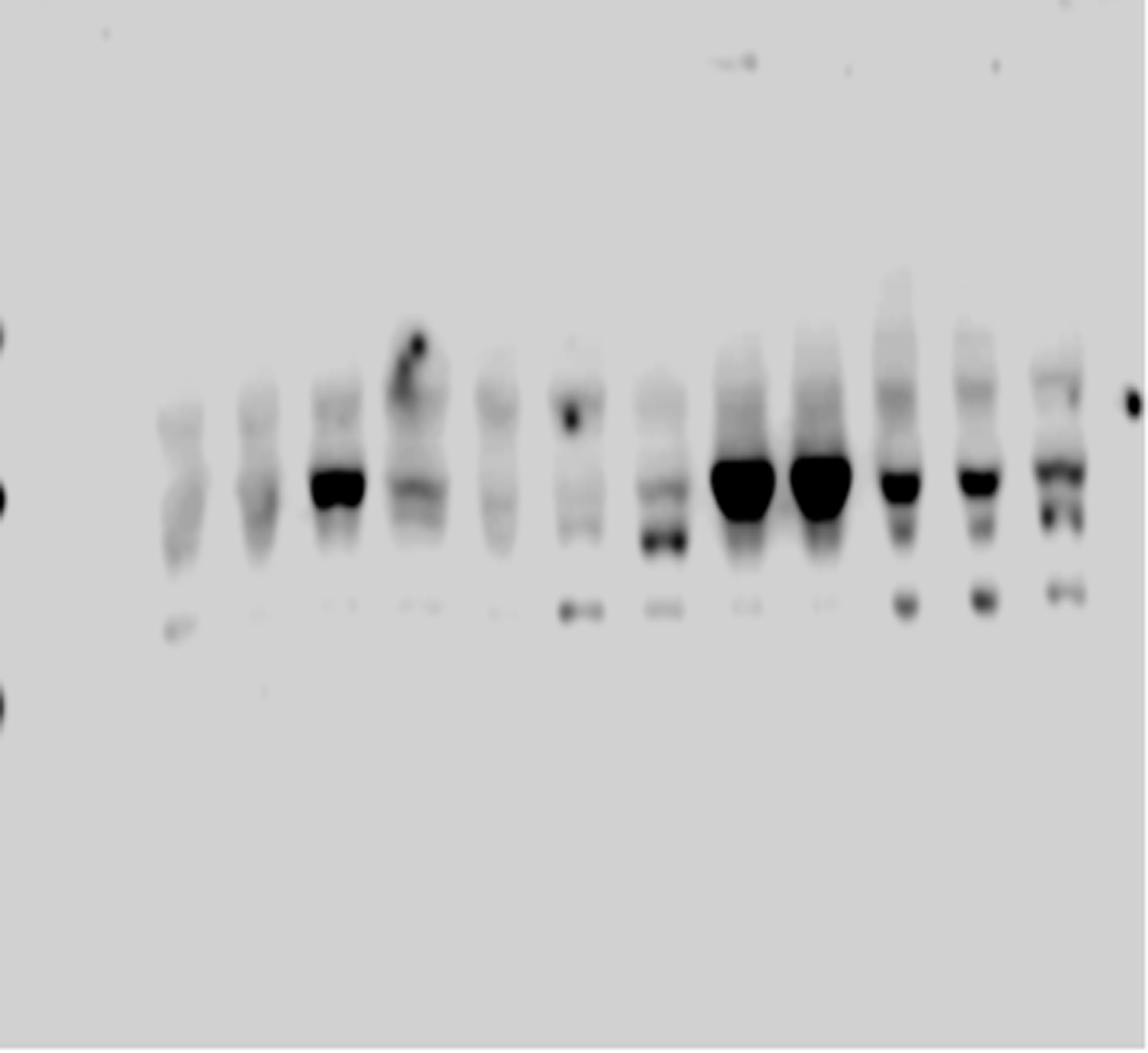

Supplement: Figure 6—source data 2. [file elife-78430-fig6-data2.zip › fig 6-source data 2/unlabeled figure 6-source data 2-alpha-SMA.tif]

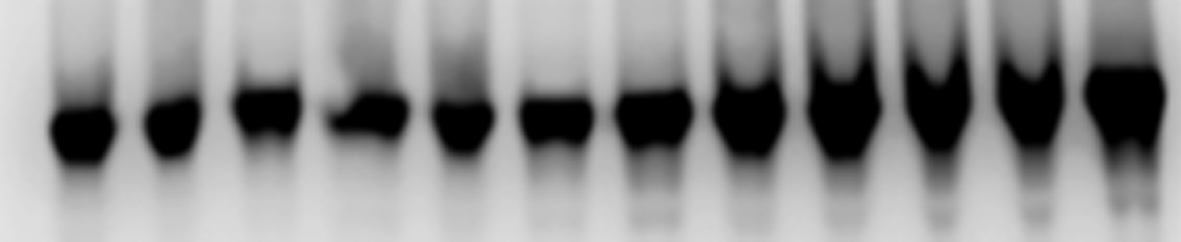

Supplement: Figure 6—source data 2. [file elife-78430-fig6-data2.zip › fig 6-source data 2/unlabeled figure 6-source data 2-GAPDH.tif]

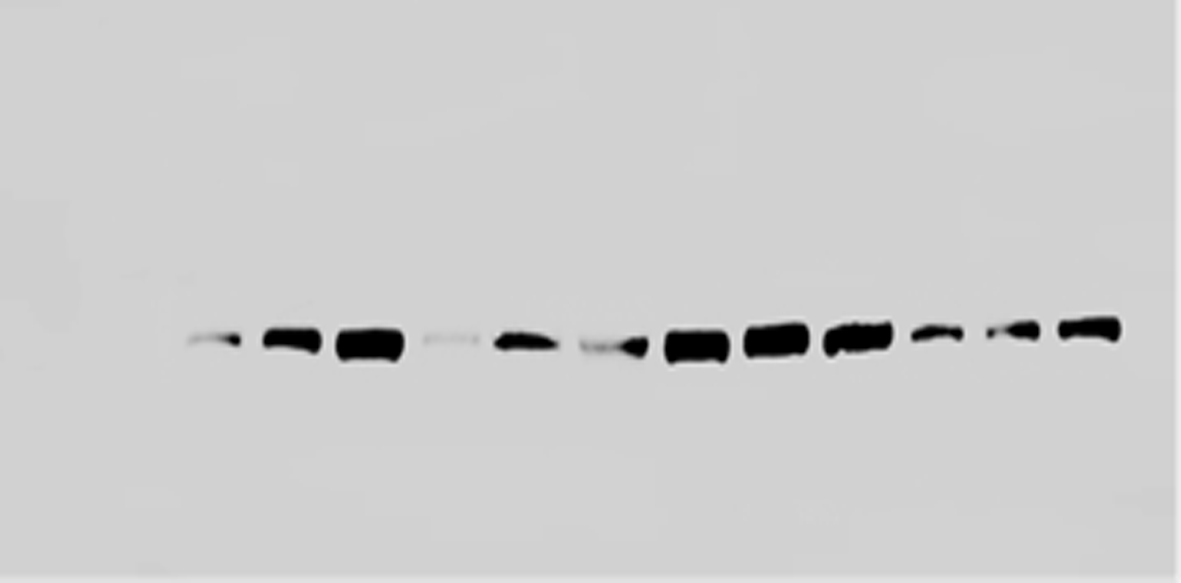

Supplement: Figure 6—source data 2. [file elife-78430-fig6-data2.zip › fig 6-source data 2/unlabeled figure 6-source data 2-hsp47.tif]

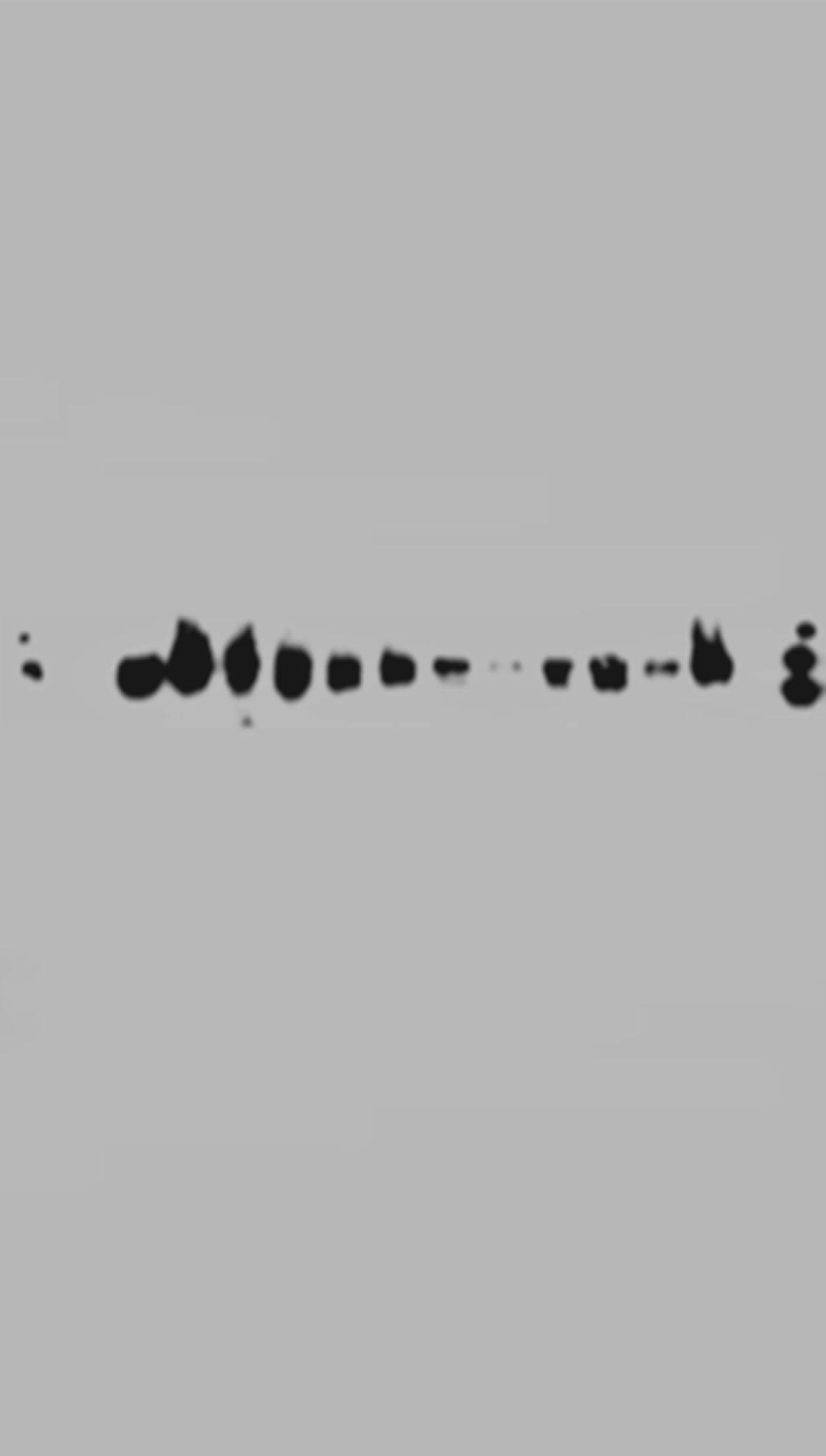

Supplement: Figure 6—source data 2. [file elife-78430-fig6-data2.zip › fig 6-source data 2/unlabeled figure 6-source data 2-IP AKAP12 wb hsp47.tif]

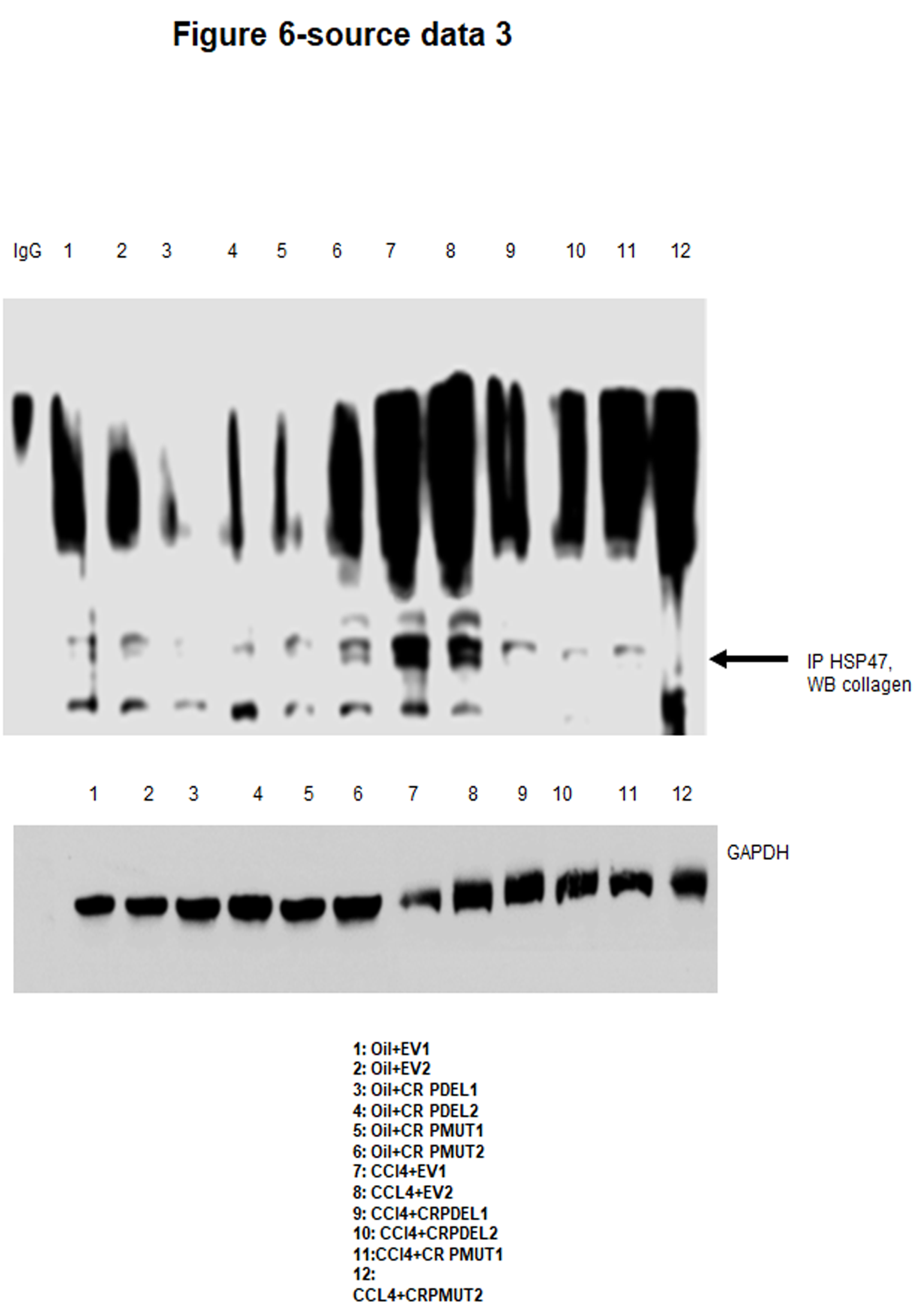

Supplement: Figure 6—source data 3. [file elife-78430-fig6-data3.zip › fig 6-source data 3/Figure 6-source data 3.tif]

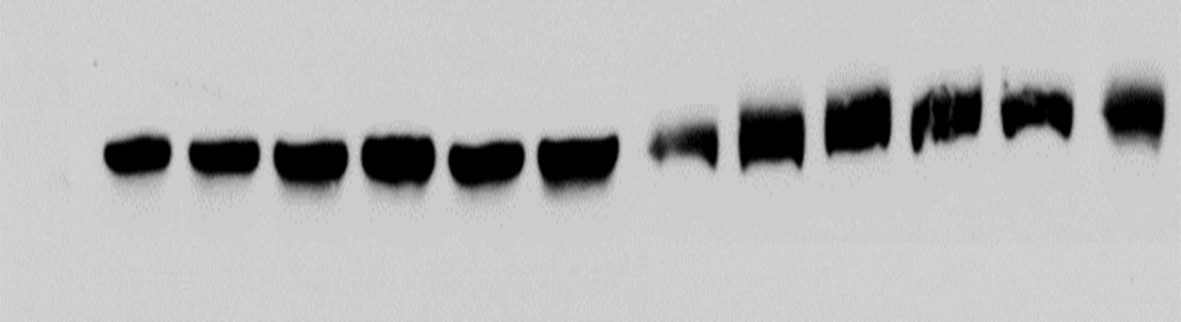

Supplement: Figure 6—source data 3. [file elife-78430-fig6-data3.zip › fig 6-source data 3/unlabeled figure 6-source data 3-GAPDH.tif]

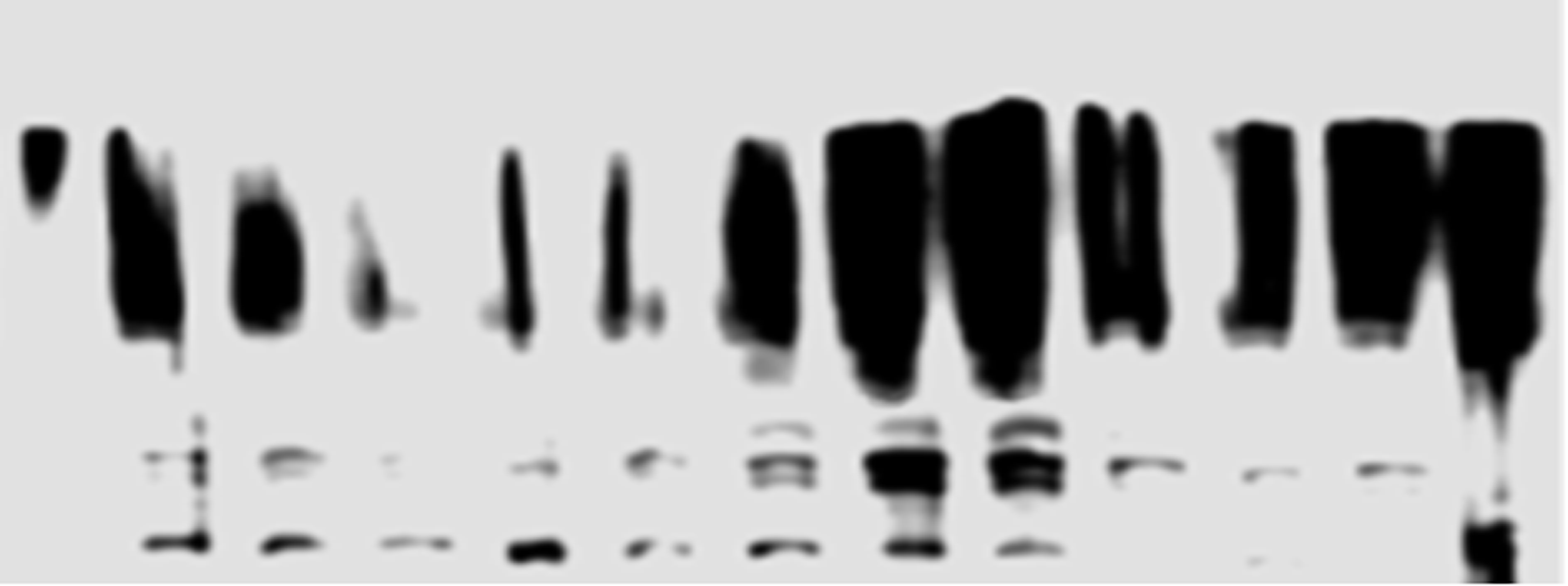

Supplement: Figure 6—source data 3. [file elife-78430-fig6-data3.zip › fig 6-source data 3/unlabeled figure 6-source data 3-IP HSP47 wb collagen.tif]

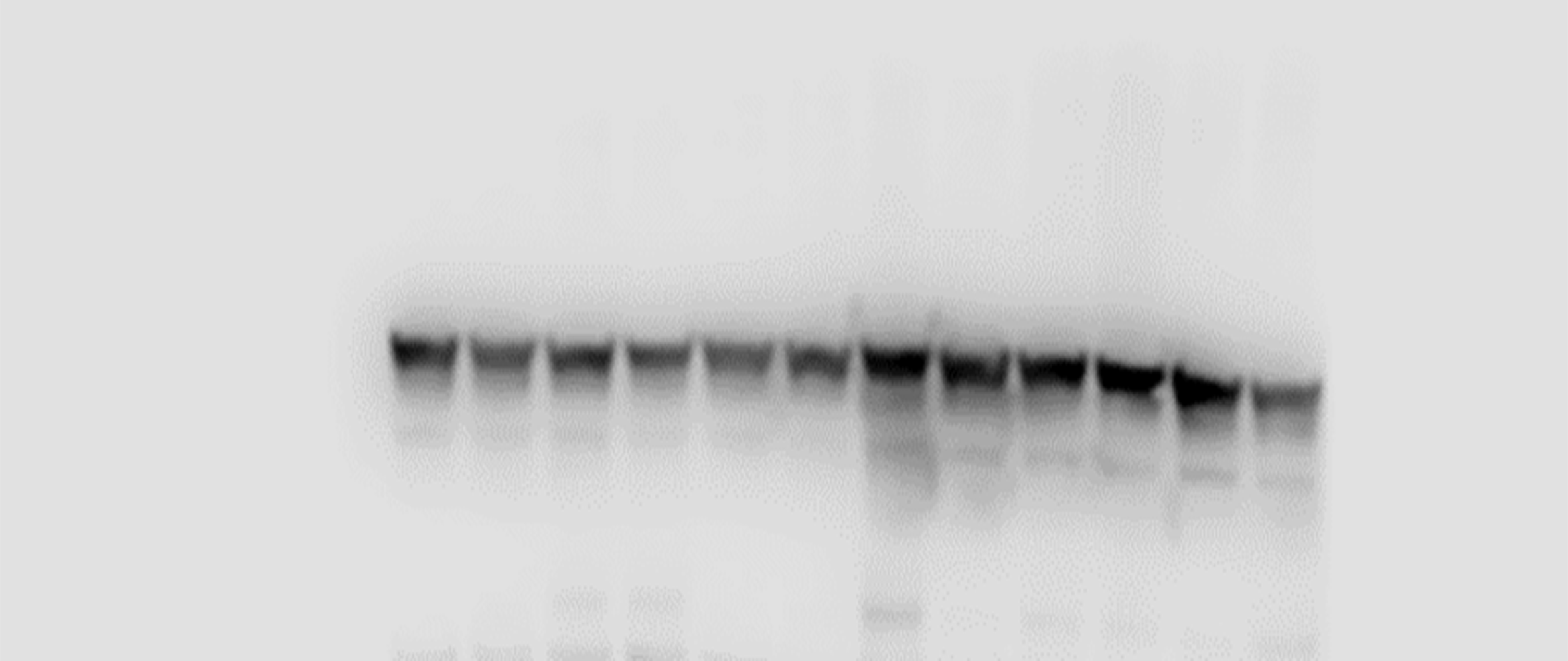

Supplement: Figure 7—source data 1. [file elife-78430-fig7-data1.zip › fig 7-source data 1/unlabeled figure 7-source data 1-BIP.tif]

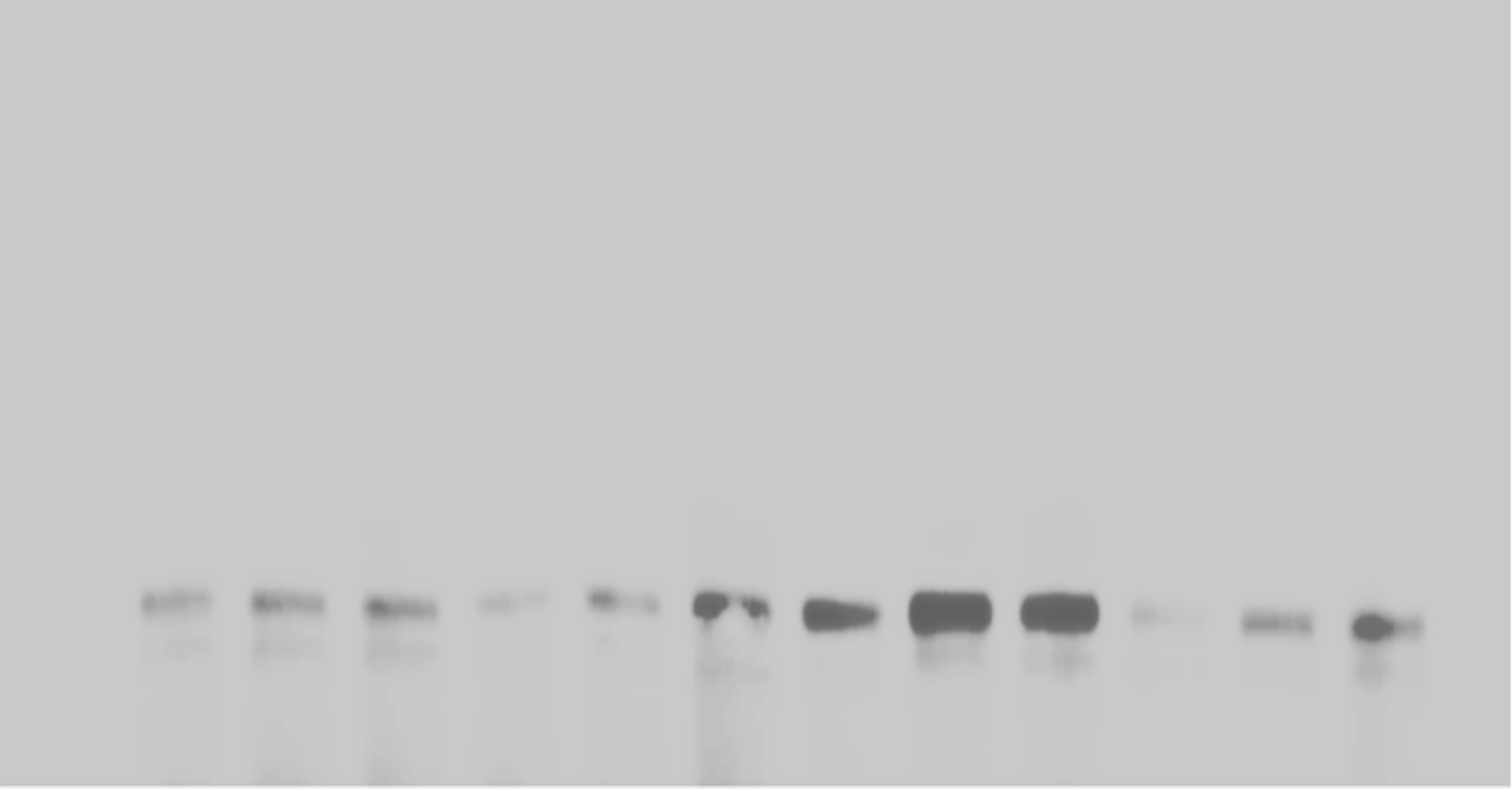

Supplement: Figure 7—source data 1. [file elife-78430-fig7-data1.zip › fig 7-source data 1/unlabeled figure 7-source data 1-IP collagen wb BIP.tif]

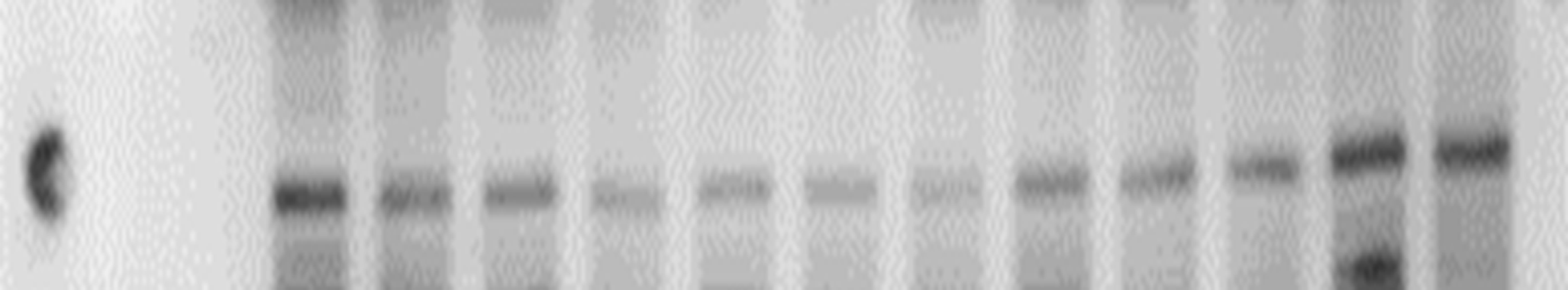

Supplement: Figure 7—source data 1. [file elife-78430-fig7-data1.zip › fig 7-source data 1/unlabeled figure 7-source data 1-IRE1 alpha.tif]

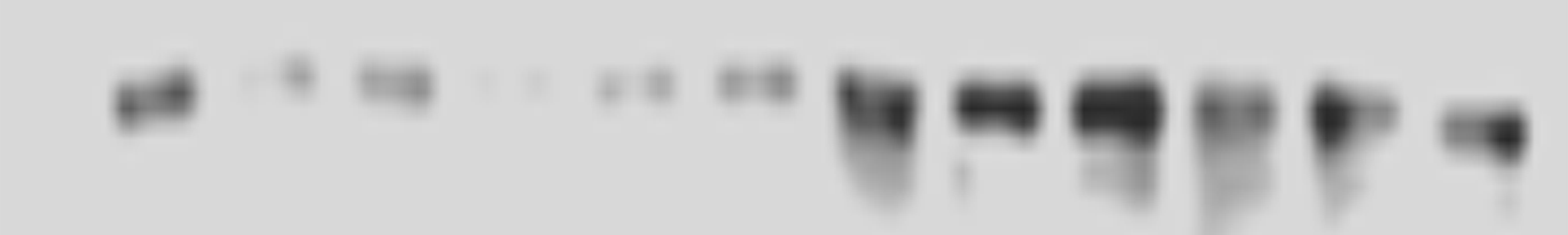

Supplement: Figure 7—source data 1. [file elife-78430-fig7-data1.zip › fig 7-source data 1/unlabeled figure 7-source data 1-IRE1 alpha-HSP47 IP.tif]

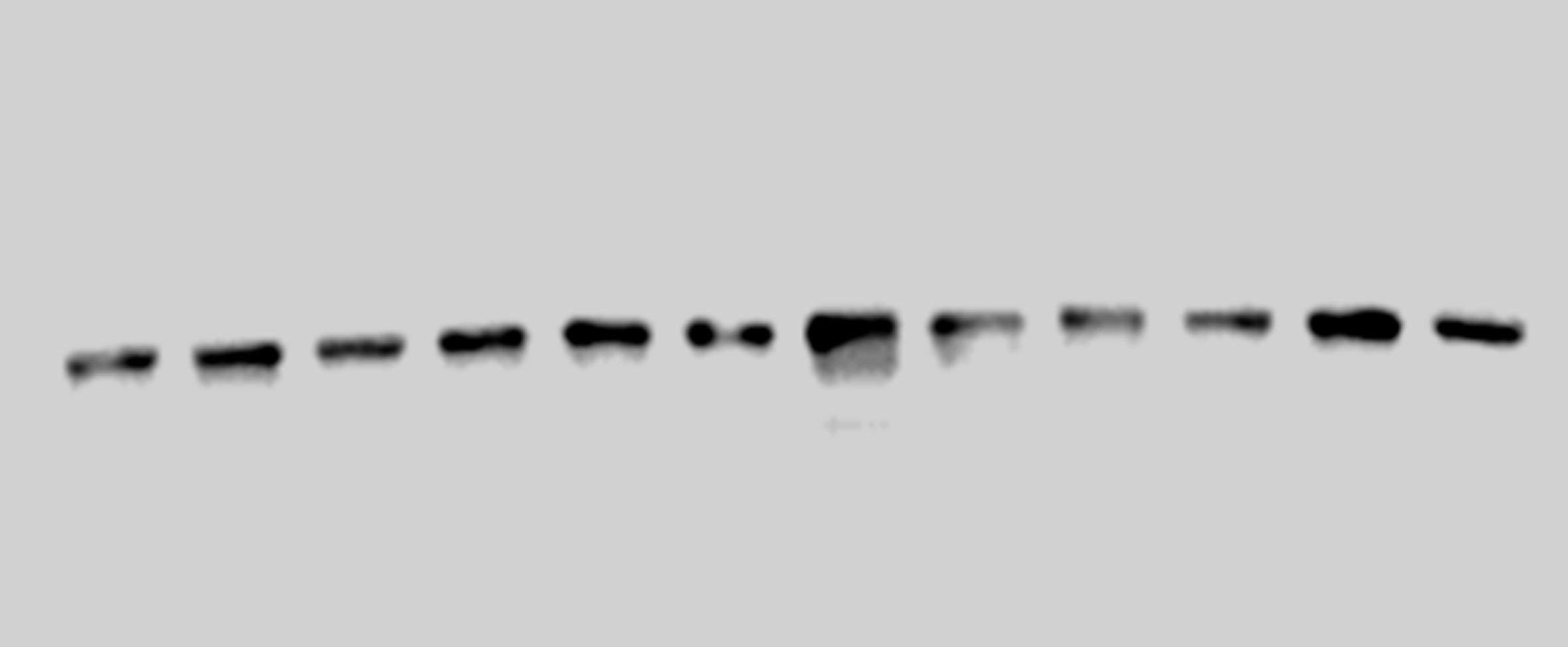

Supplement: Figure 7—source data 1. [file elife-78430-fig7-data1.zip › fig 7-source data 1/unlabeled figure 7-source data 1-P38 MAPK.tif]

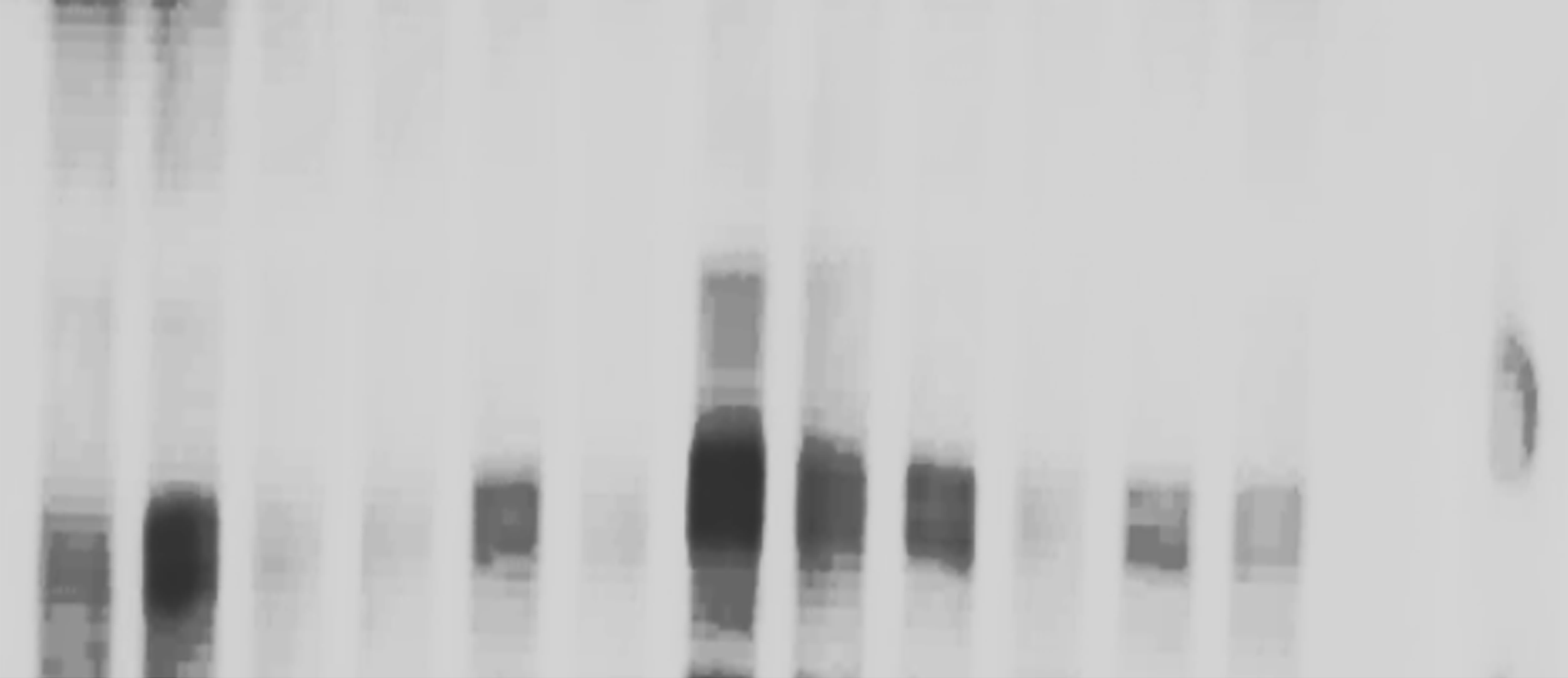

Supplement: Figure 7—source data 1. [file elife-78430-fig7-data1.zip › fig 7-source data 1/unlabeled figure 7-source data 1-P-IRE1 alpha (S724).tif]

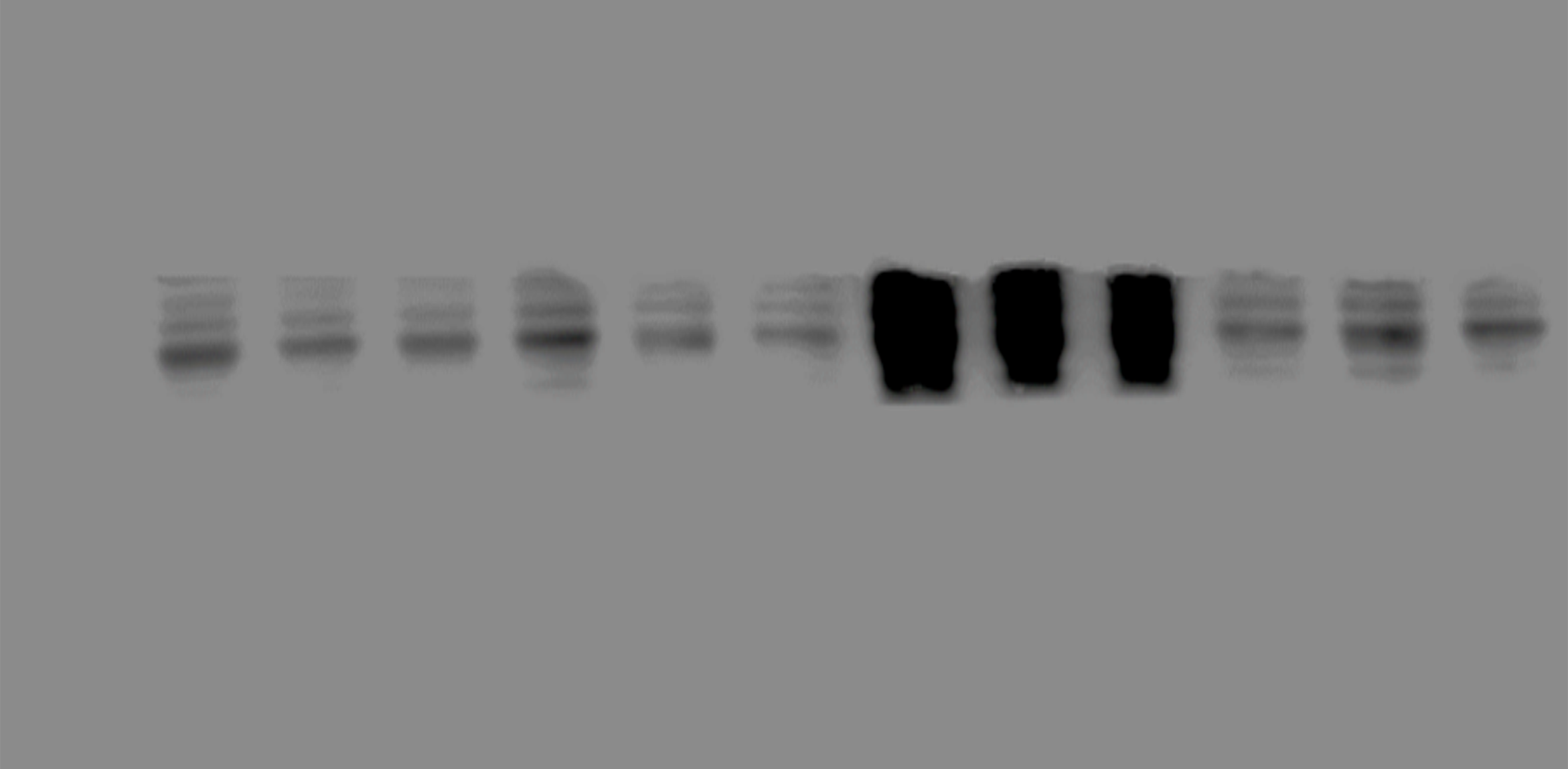

Supplement: Figure 7—source data 1. [file elife-78430-fig7-data1.zip › fig 7-source data 1/unlabeled figure 7-source data 1-P-P38 MAPK.tif]

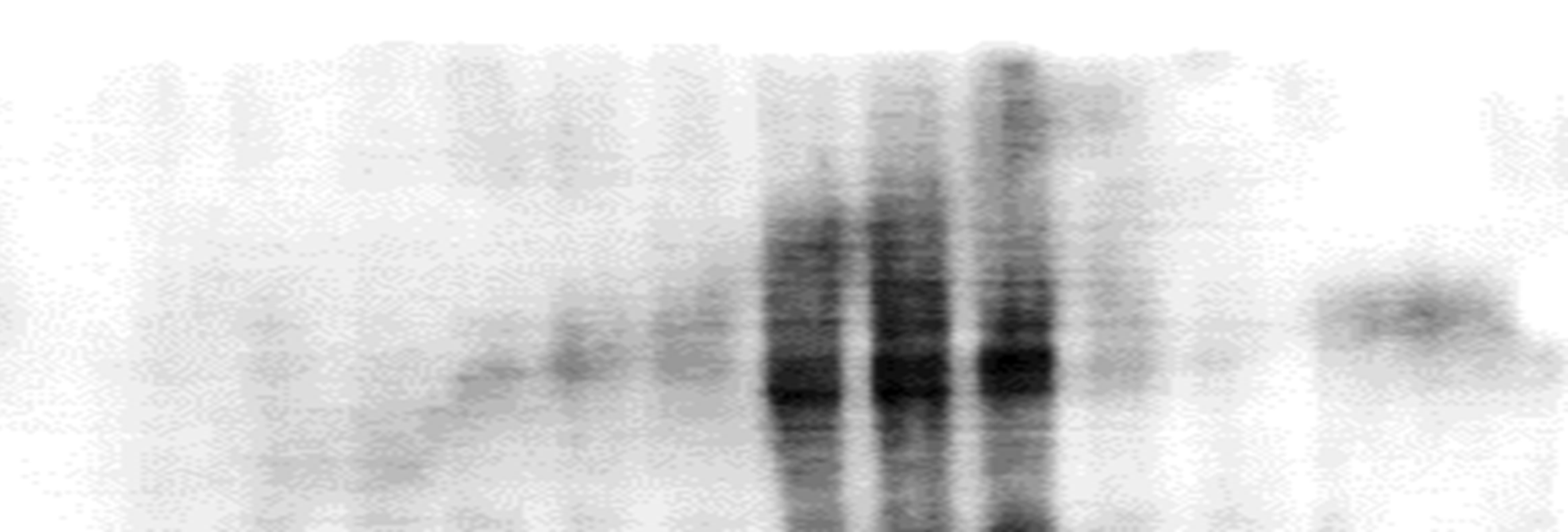

Supplement: Figure 7—source data 1. [file elife-78430-fig7-data1.zip › fig 7-source data 1/unlabeled figure 7-source data 1-P-SMAD2,3.tif]

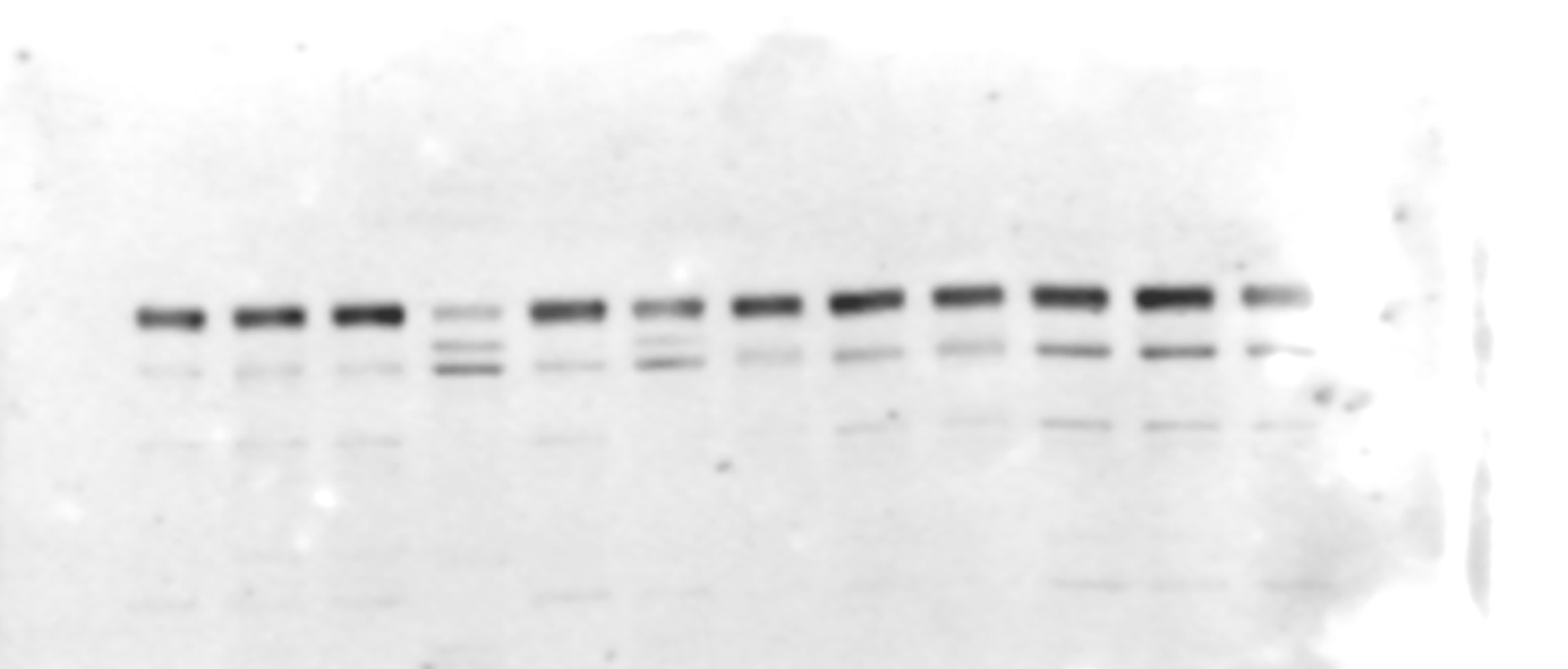

Supplement: Figure 7—source data 1. [file elife-78430-fig7-data1.zip › fig 7-source data 1/unlabeled figure 7-source data 1-SMAD2,3.tif]

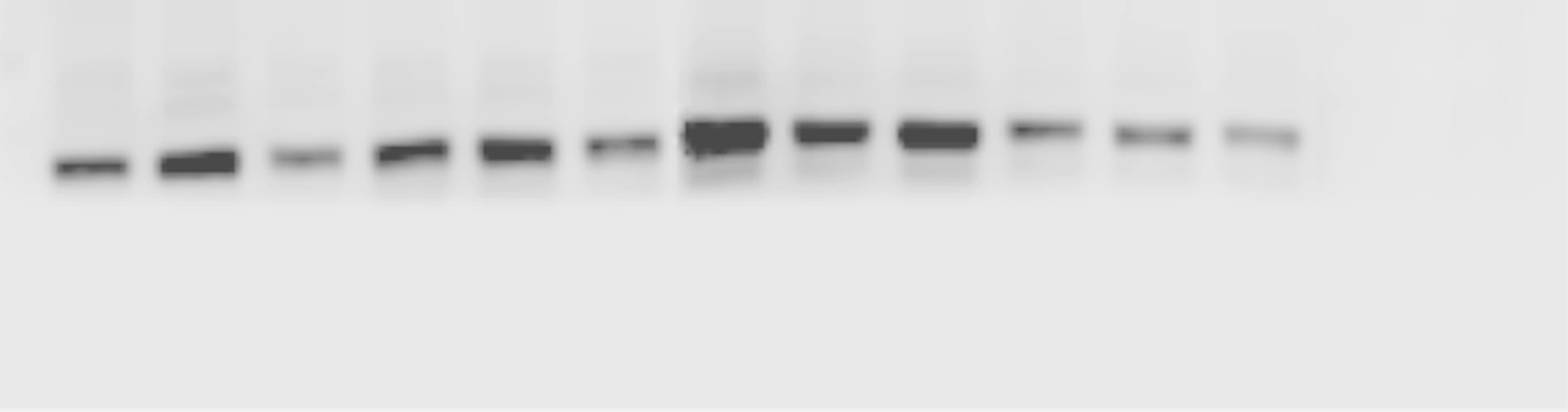

Supplement: Figure 7—source data 2. [file elife-78430-fig7-data2.zip › fig 7-source data 2/unlabeled figure 7-source data 2-BIP.tif]

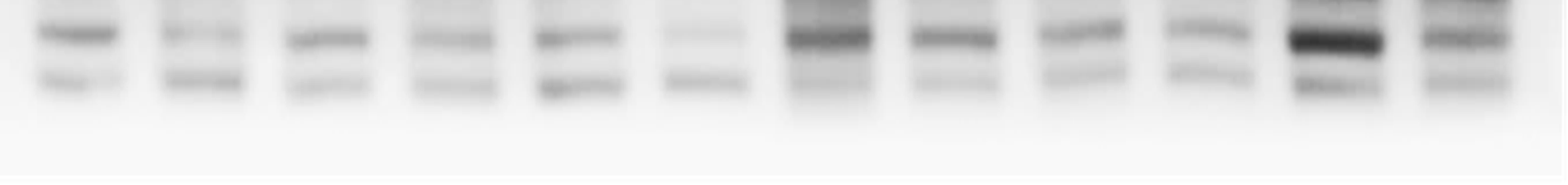

Supplement: Figure 7—source data 2. [file elife-78430-fig7-data2.zip › fig 7-source data 2/unlabeled figure 7-source data 2-IRE1 alpha.tif]

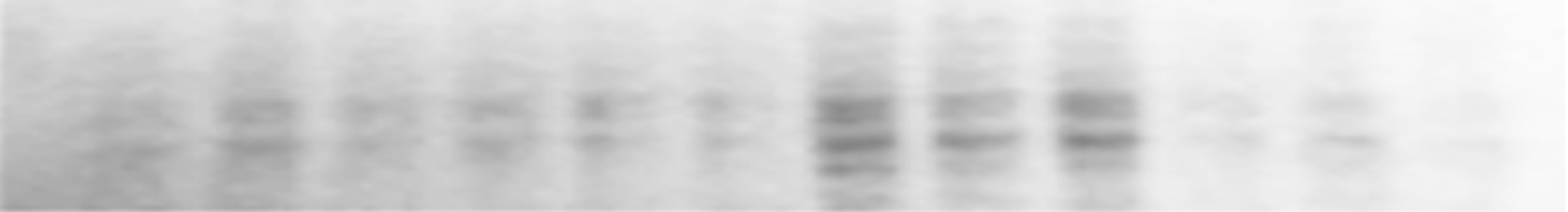

Supplement: Figure 7—source data 2. [file elife-78430-fig7-data2.zip › fig 7-source data 2/unlabeled figure 7-source data 2-P-IRE1 alpha (S724).tif]

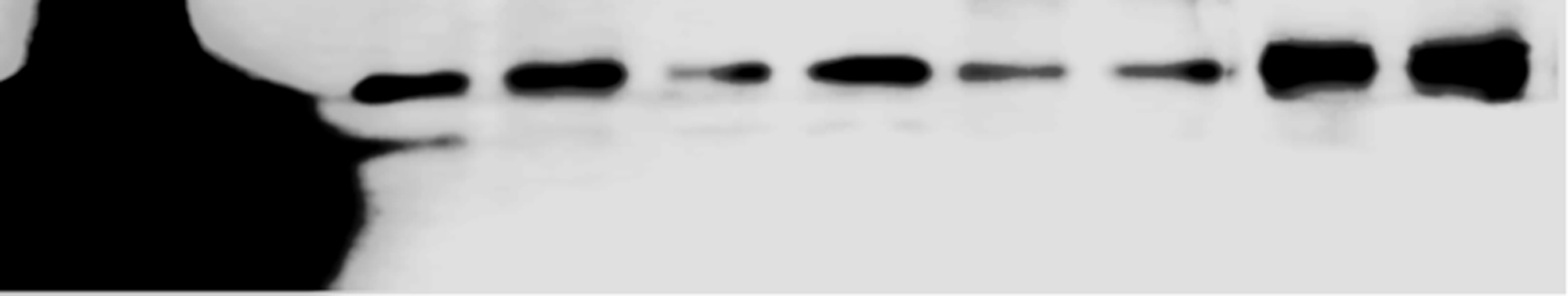

Supplement: Figure 7—source data 3. [file elife-78430-fig7-data3.zip › fig 7-source data 3/unlabeled Figure 7-source data 3-IL-10.tif]

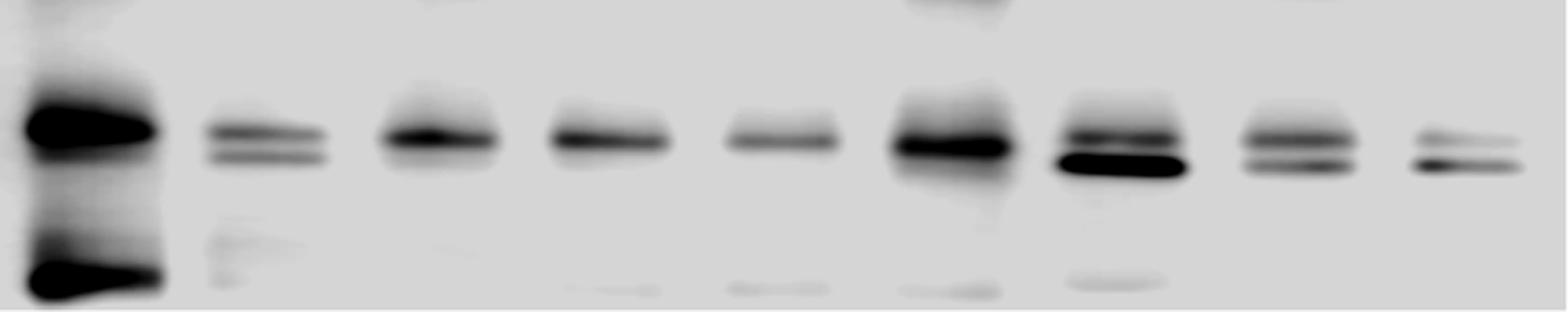

Supplement: Figure 7—source data 3. [file elife-78430-fig7-data3.zip › fig 7-source data 3/unlabeled Figure 7-source data 3-IL-17.tif]

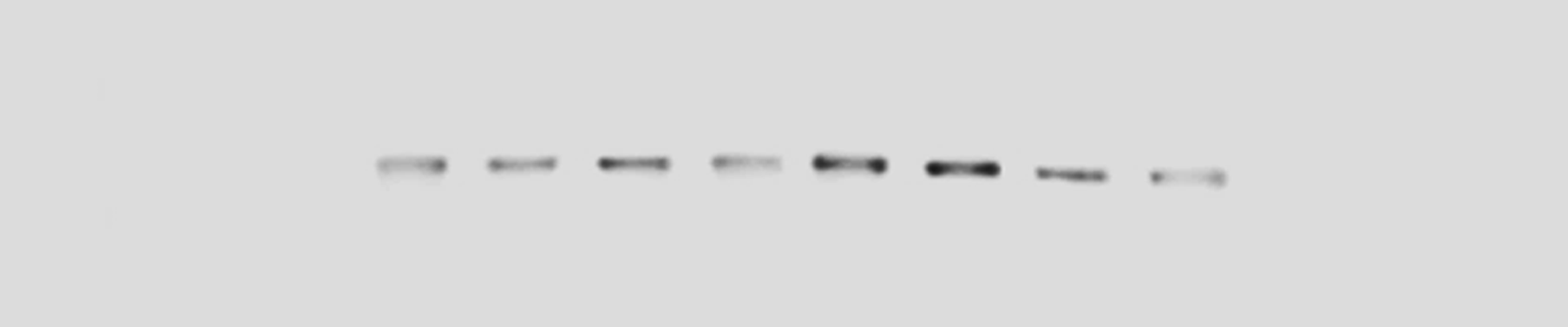

Supplement: Figure 7—source data 3. [file elife-78430-fig7-data3.zip › fig 7-source data 3/unlabeled Figure 7-source data 3-IL-1beta.tif]

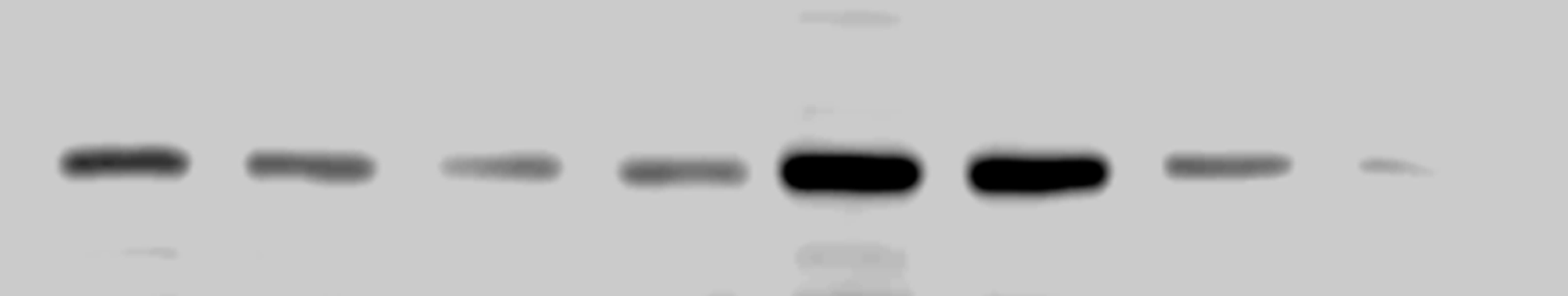

Supplement: Figure 7—source data 3. [file elife-78430-fig7-data3.zip › fig 7-source data 3/unlabeled Figure 7-source data 3-IL-6.tif]

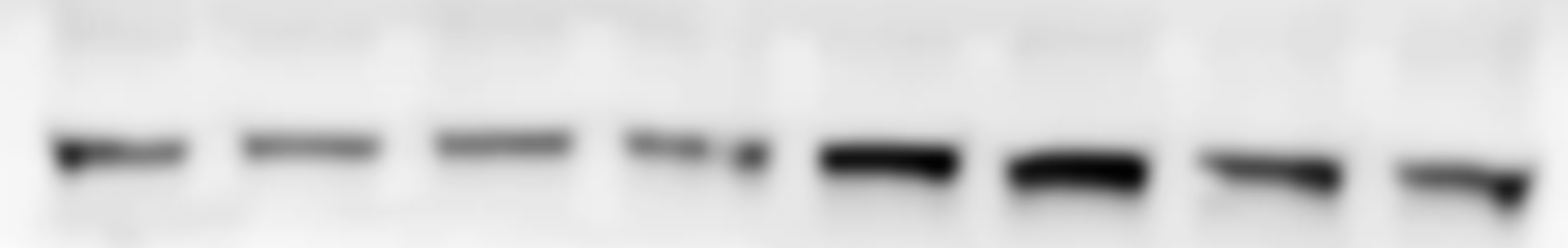

Supplement: Figure 7—source data 4. [file elife-78430-fig7-data4.zip › fig7-source data 4/unlabeled Figure 7-source data 4-BIP.tif]

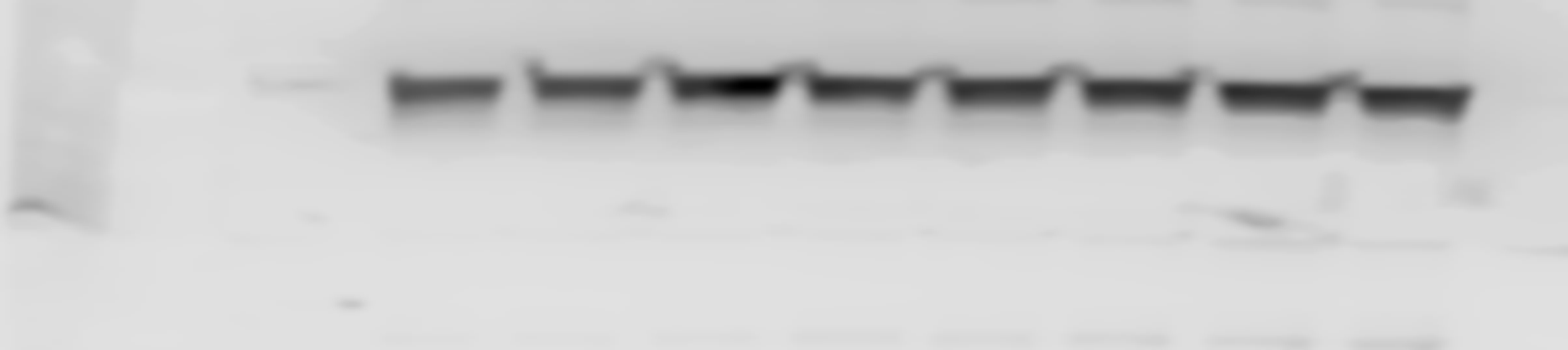

Supplement: Figure 7—source data 4. [file elife-78430-fig7-data4.zip › fig7-source data 4/unlabeled Figure 7-source data 4-IRE1 alpha.tif]

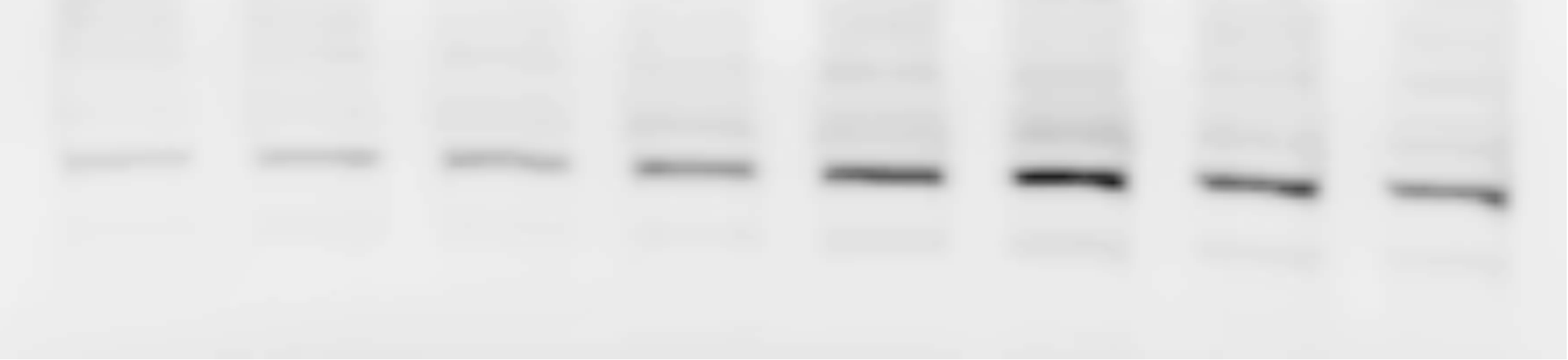

Supplement: Figure 7—source data 4. [file elife-78430-fig7-data4.zip › fig7-source data 4/unlabeled Figure 7-source data 4-P-IRE1 (S724).tif]

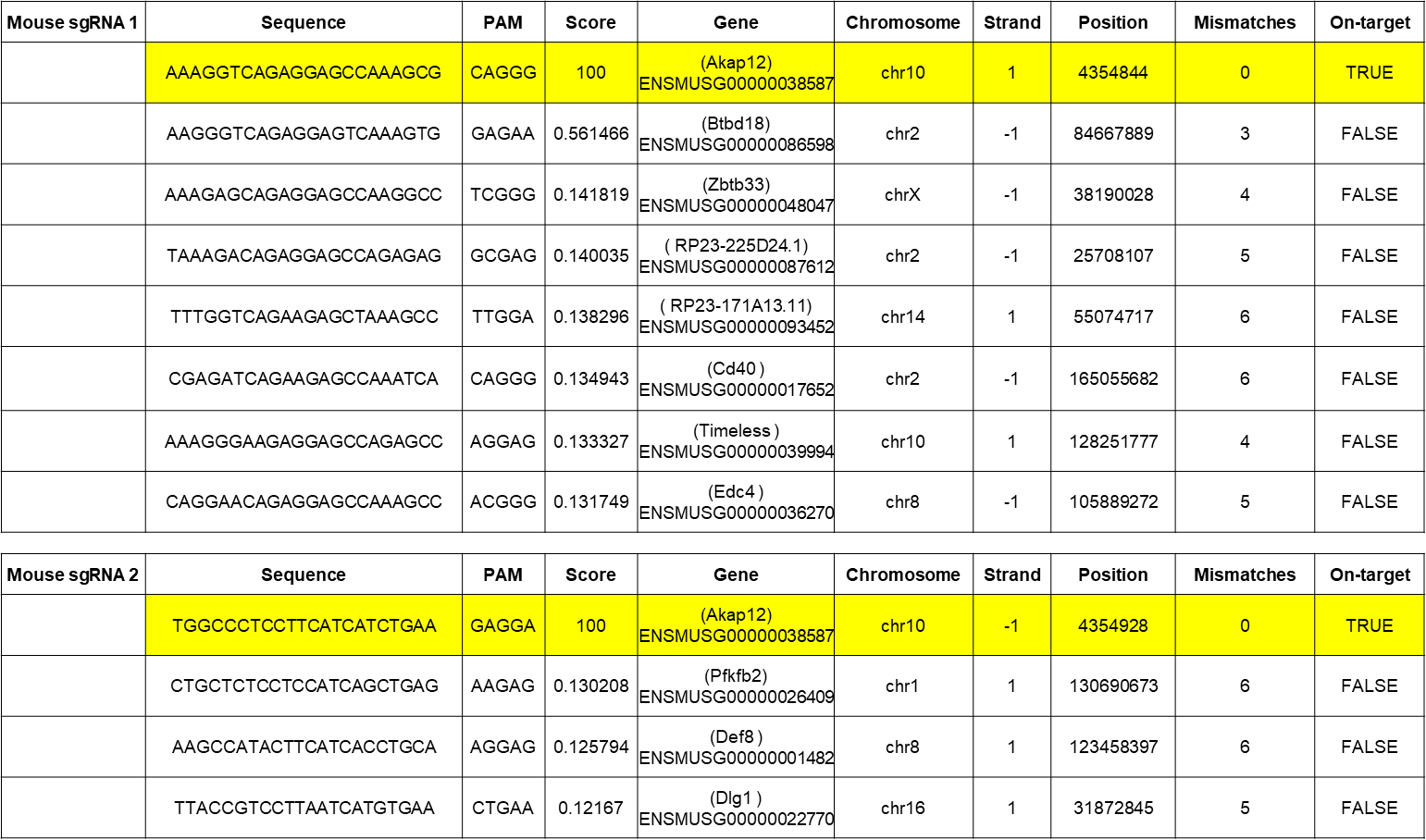


**Supplementary Table S5. Mouse sgRNA off-target analysis**

Supplement: Supplementary file 5. [file elife-78430-supp5.docx]
