# Supplementary material for: Targeting A-kinase anchoring protein 12 phosphorylation in hepatic stellate cells regulates liver injury and fibrosis in mouse models
Source: eLife. 2022 Oct 4;11:e78430. doi: 10.7554/eLife.78430 (PMC9531947; doi:10.7554/eLife.78430)
Supplement: Figure 1—source data 2. [file elife-78430-fig1-data2.pptx]

## Slide 1
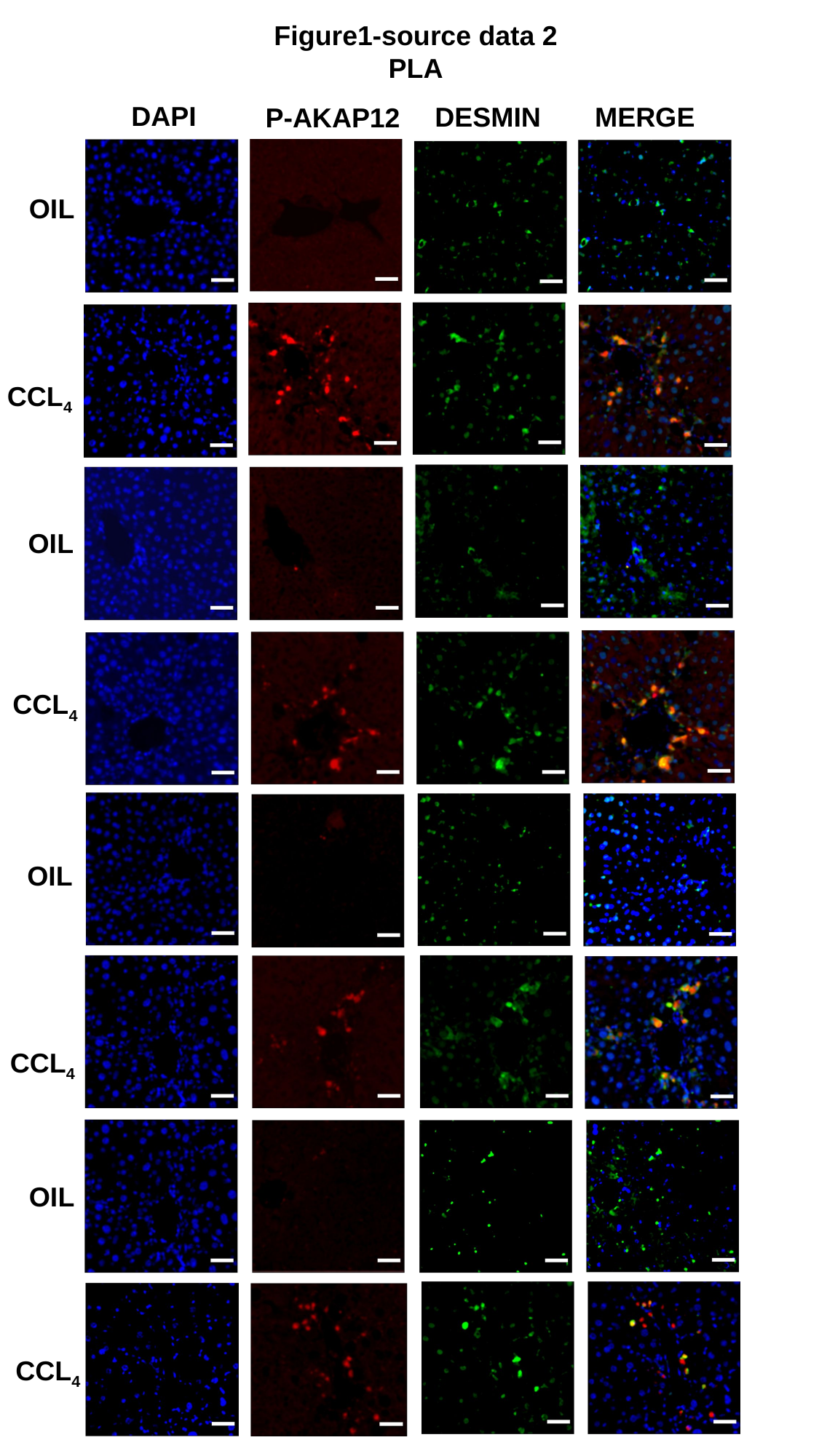

Figure1-source data 2
PLA
DAPI
DESMIN
MERGE
P-AKAP12
OIL
CCL4
OIL
CCL4
OIL
CCL4
OIL
CCL4

## Slide 2
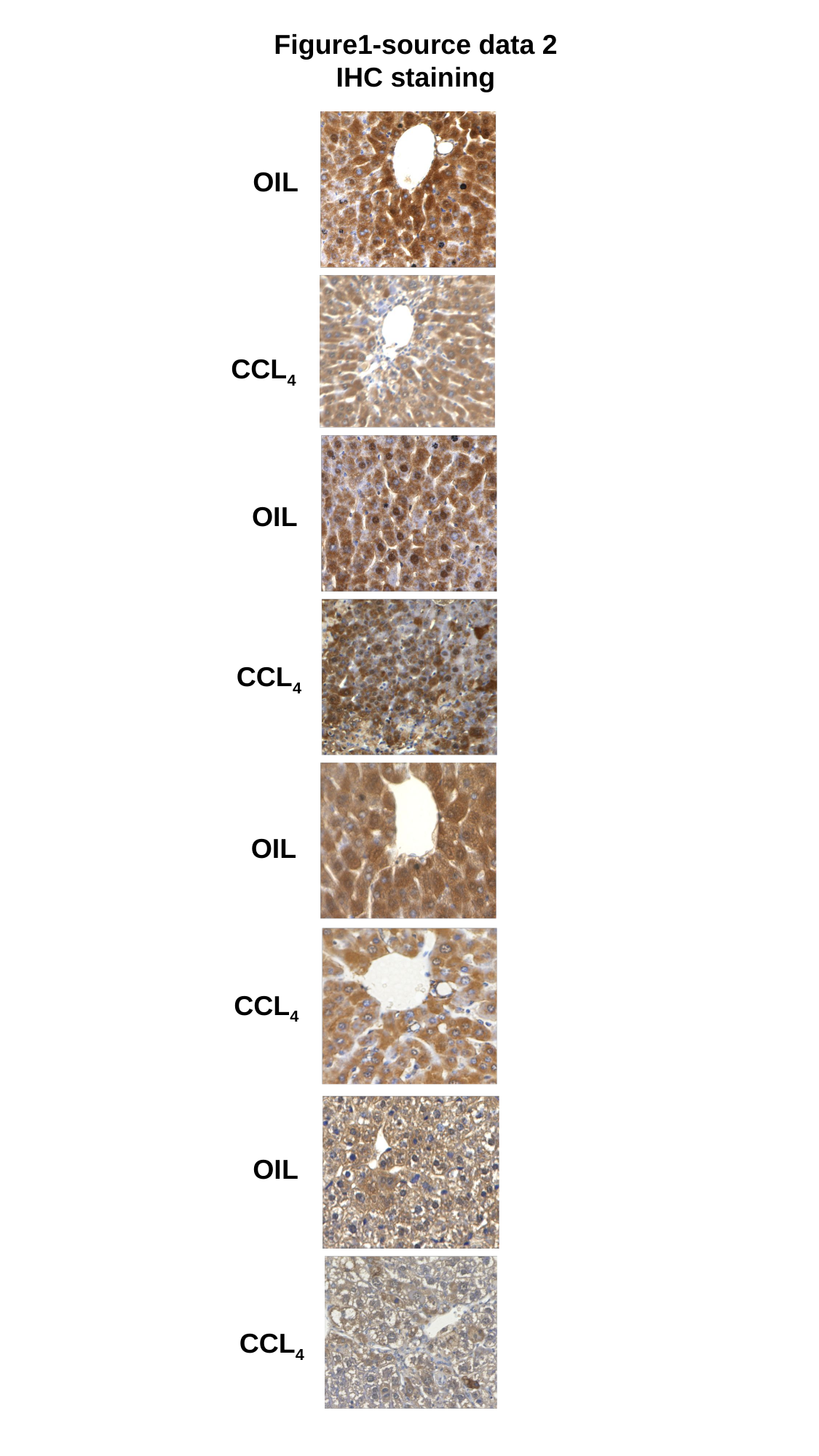

Figure1-source data 2
IHC staining
OIL
CCL4
OIL
CCL4
OIL
CCL4
OIL
CCL4
