# Supplementary material for: Targeting A-kinase anchoring protein 12 phosphorylation in hepatic stellate cells regulates liver injury and fibrosis in mouse models
Source: eLife. 2022 Oct 4;11:e78430. doi: 10.7554/eLife.78430 (PMC9531947; doi:10.7554/eLife.78430)
Supplement: Table 1—source data 1. [file elife-78430-table1-data1.pptx]

## Slide 1
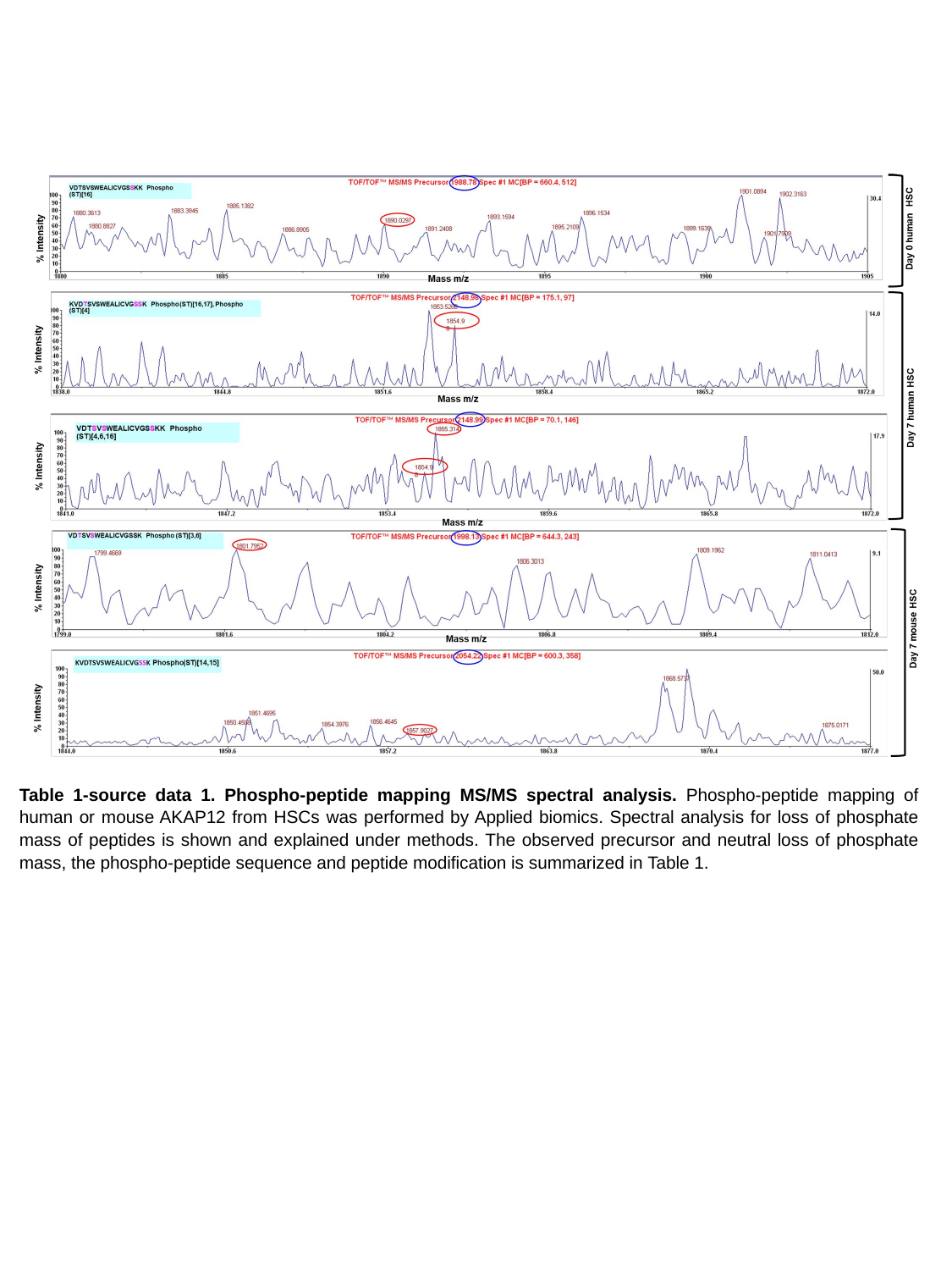

Table 1-source data 1. Phospho-peptide mapping MS/MS spectral analysis. Phospho-peptide mapping of human or mouse AKAP12 from HSCs was performed by Applied biomics. Spectral analysis for loss of phosphate mass of peptides is shown and explained under methods. The observed precursor and neutral loss of phosphate mass, the phospho-peptide sequence and peptide modification is summarized in Table 1.
