# Supplementary material for: Targeting A-kinase anchoring protein 12 phosphorylation in hepatic stellate cells regulates liver injury and fibrosis in mouse models
Source: eLife. 2022 Oct 4;11:e78430. doi: 10.7554/eLife.78430 (PMC9531947; doi:10.7554/eLife.78430)
Supplement: Figure 2—source data 3. [file elife-78430-fig2-data3.pptx]

## Slide 1
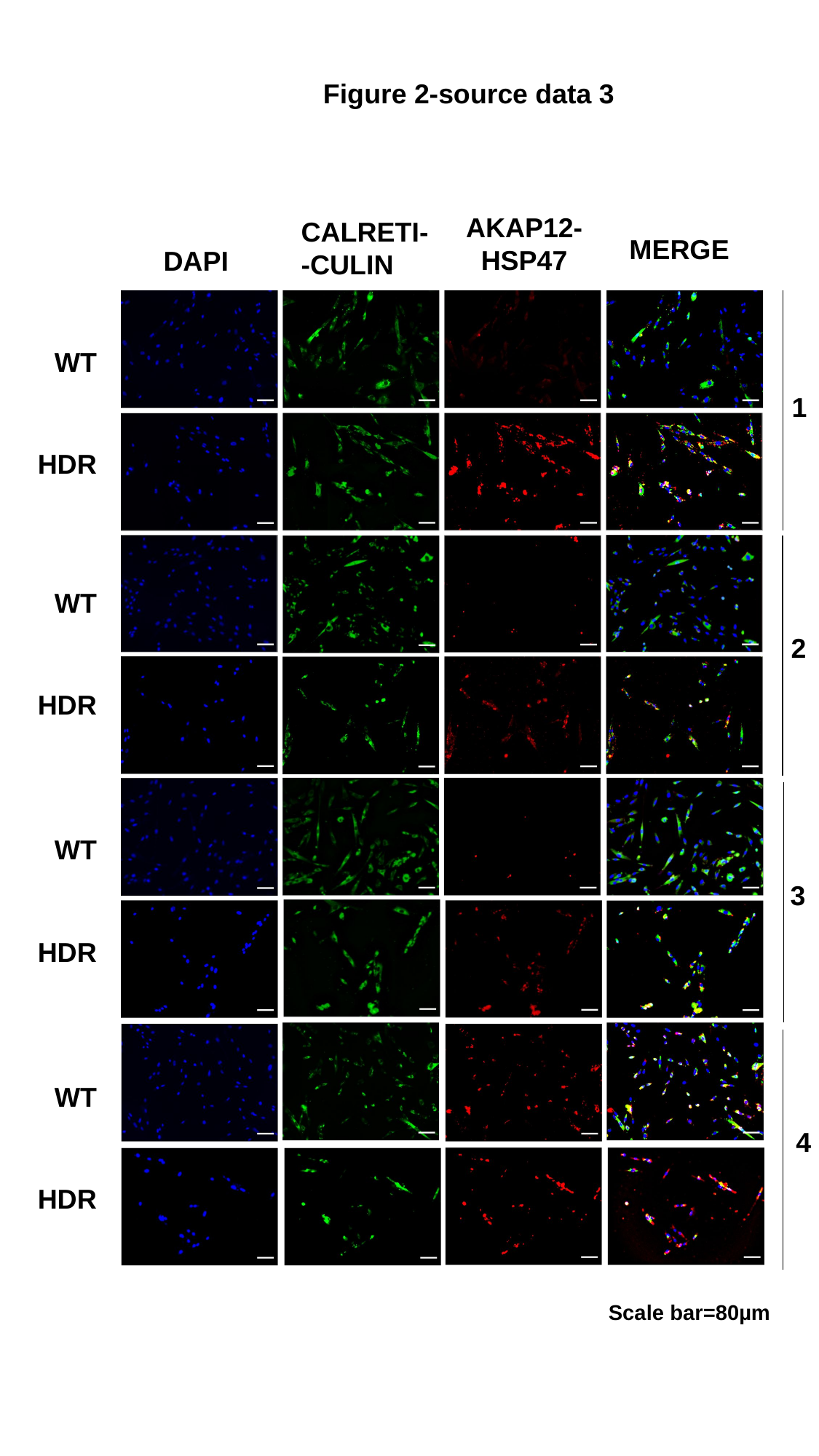

Figure 2-source data 3
AKAP12-
HSP47
CALRETI-
-CULIN
MERGE
DAPI
WT
1
HDR
WT
2
HDR
WT
3
HDR
WT
4
HDR
Scale bar=80µm

## Slide 2
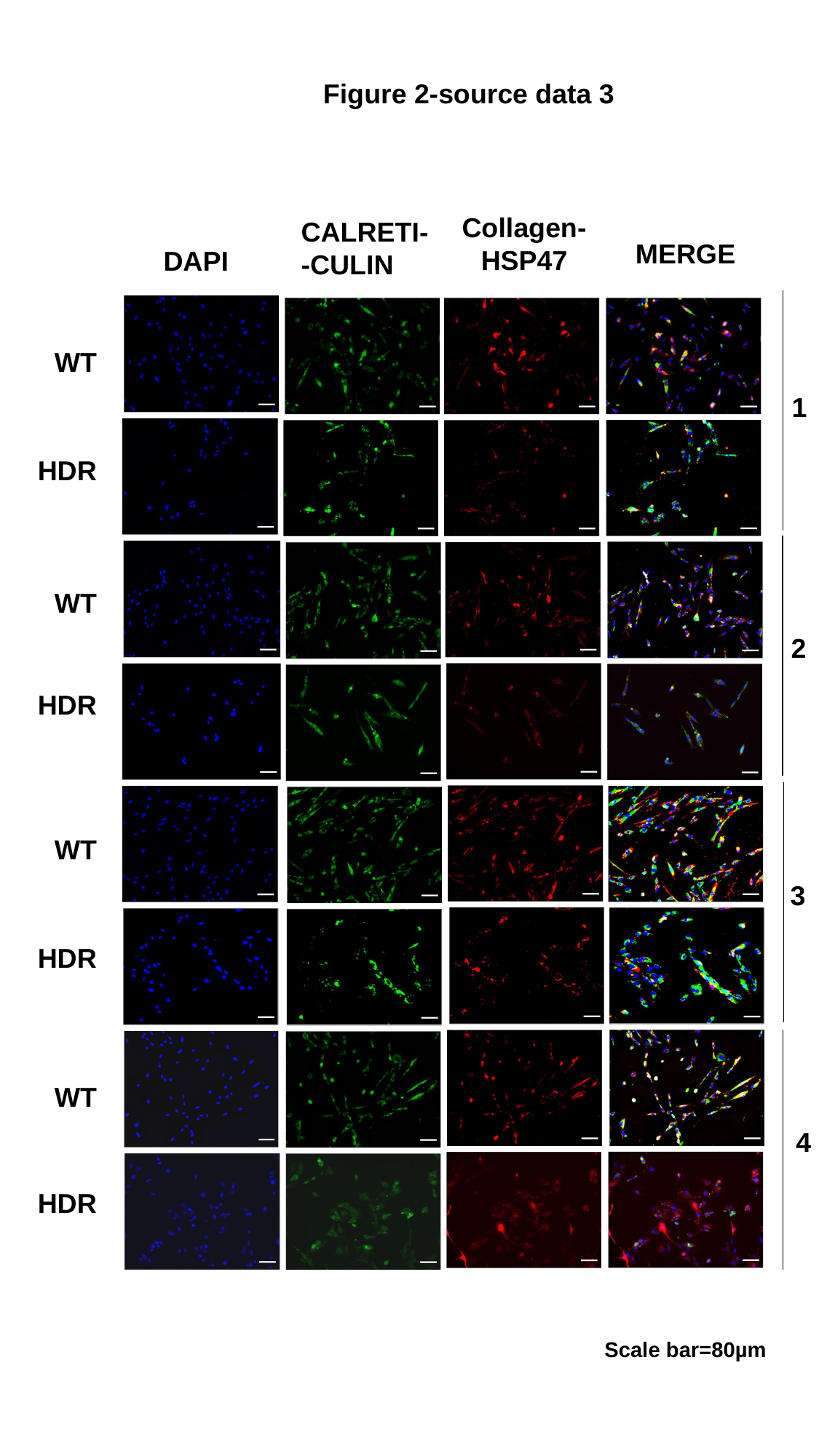

Figure 2-source data 3
Collagen-
HSP47
CALRETI-
-CULIN
MERGE
DAPI
WT
1
HDR
WT
2
HDR
WT
3
HDR
WT
4
HDR
Scale bar=80µm
