# Supplementary material for: Targeting A-kinase anchoring protein 12 phosphorylation in hepatic stellate cells regulates liver injury and fibrosis in mouse models
Source: eLife. 2022 Oct 4;11:e78430. doi: 10.7554/eLife.78430 (PMC9531947; doi:10.7554/eLife.78430)
Supplement: Figure 2—source data 5. [file elife-78430-fig2-data5.pptx]

## Slide 1
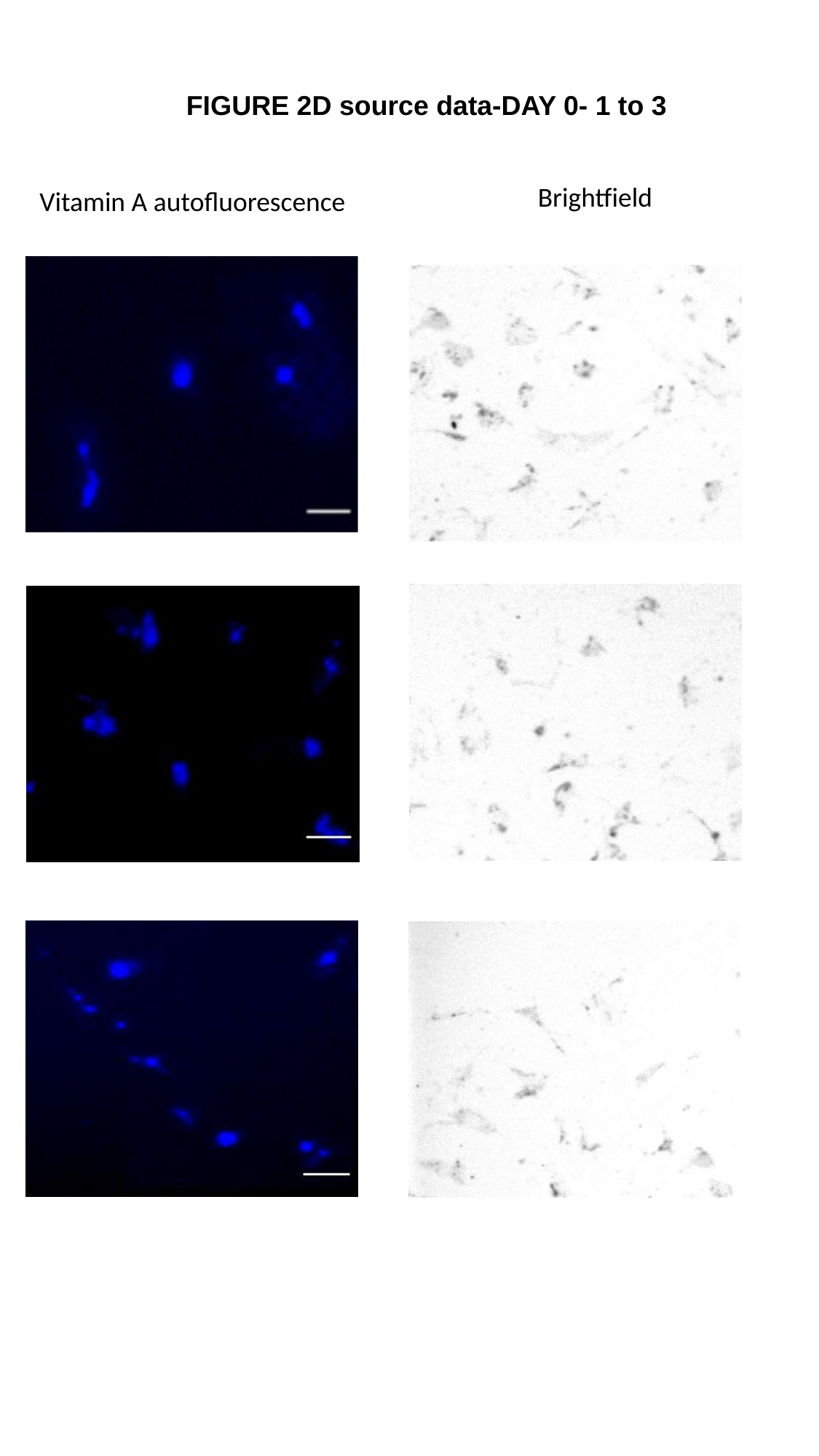

FIGURE 2D source data-DAY 0- 1 to 3
Brightfield
Vitamin A autofluorescence

## Slide 2
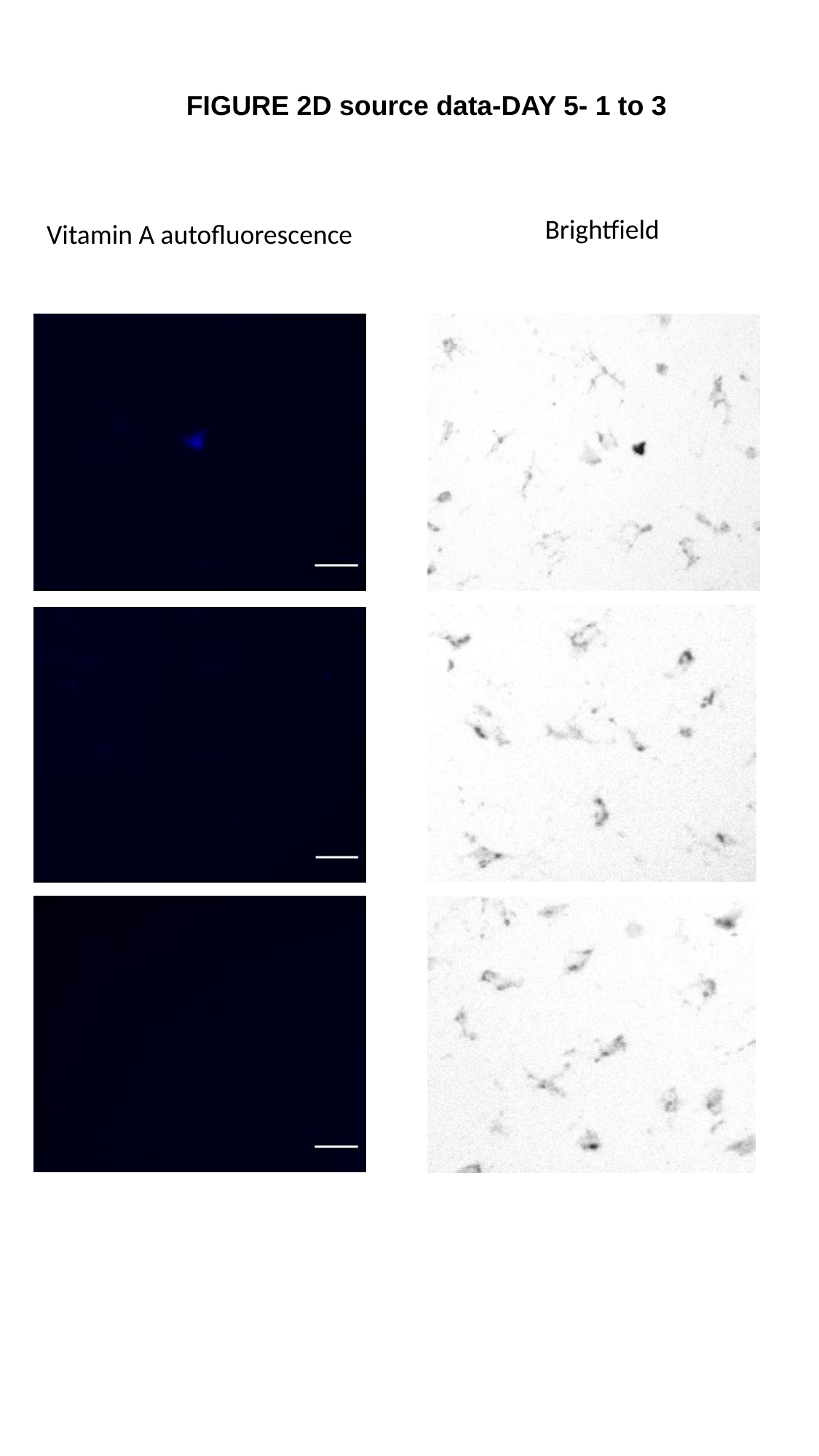

FIGURE 2D source data-DAY 5- 1 to 3
Brightfield
Vitamin A autofluorescence

## Slide 3
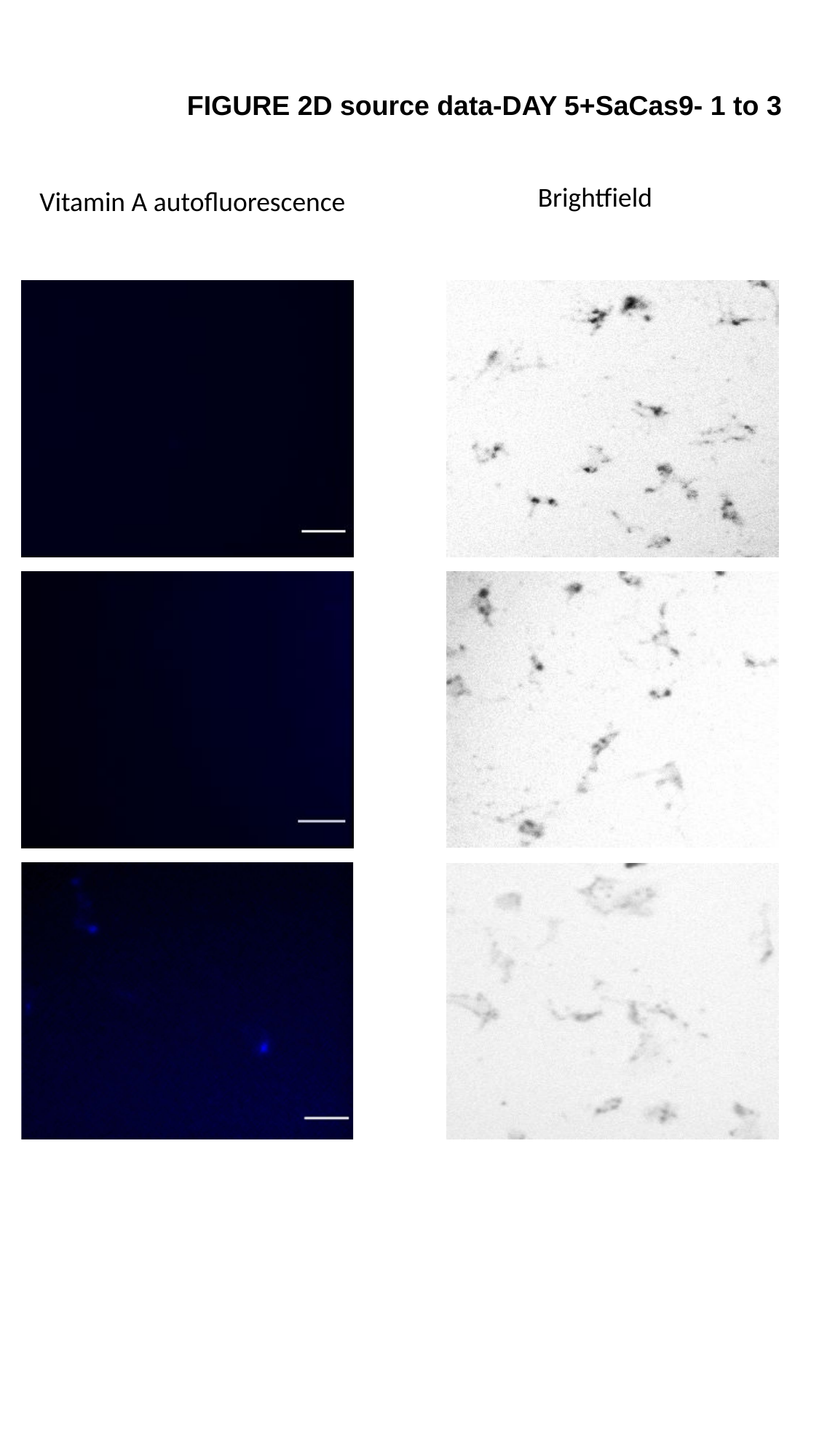

FIGURE 2D source data-DAY 5+SaCas9- 1 to 3
Brightfield
Vitamin A autofluorescence

## Slide 4
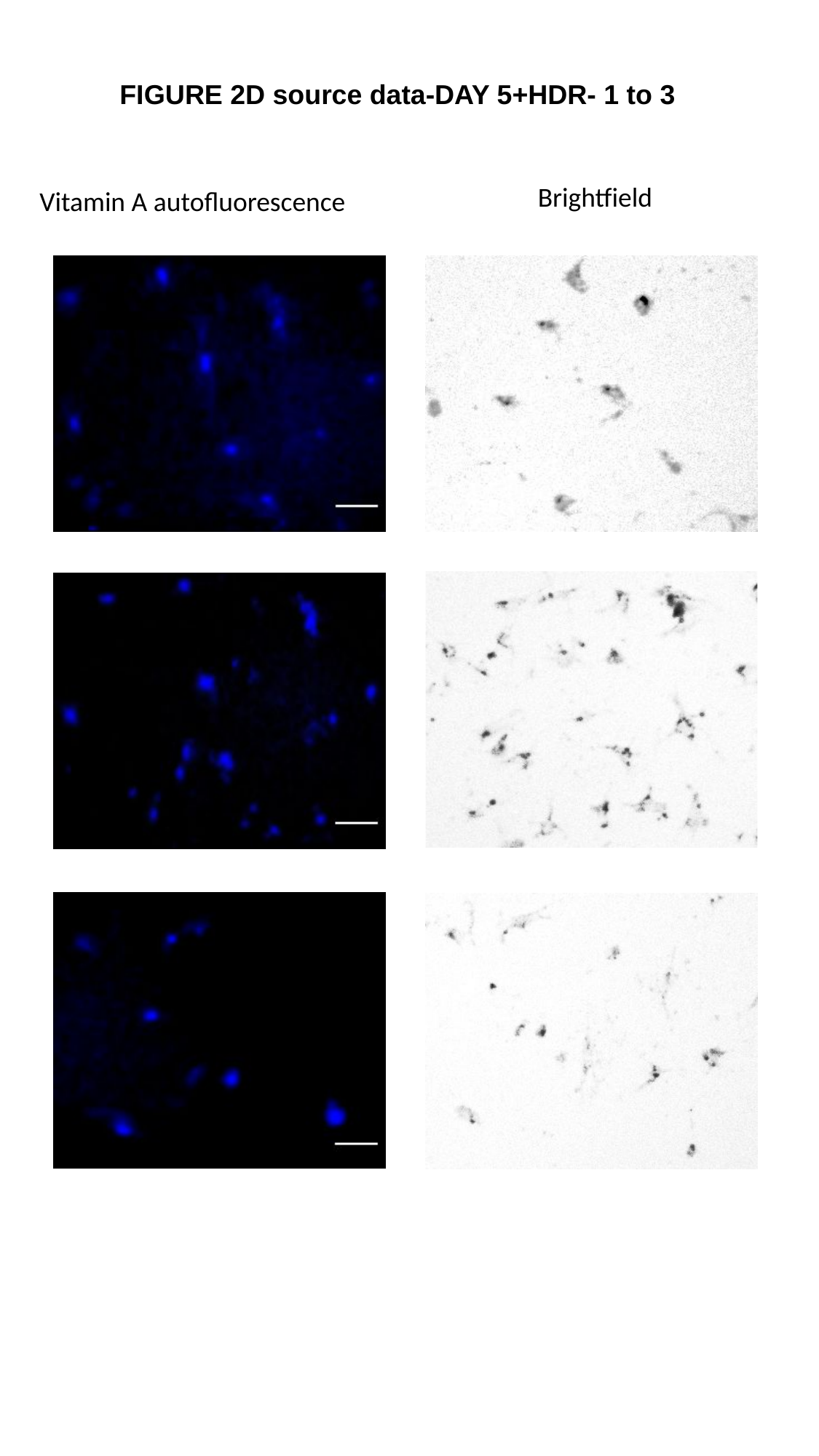

FIGURE 2D source data-DAY 5+HDR- 1 to 3
Brightfield
Vitamin A autofluorescence
