# Supplementary material for: Targeting A-kinase anchoring protein 12 phosphorylation in hepatic stellate cells regulates liver injury and fibrosis in mouse models
Source: eLife. 2022 Oct 4;11:e78430. doi: 10.7554/eLife.78430 (PMC9531947; doi:10.7554/eLife.78430)
Supplement: Figure 5—source data 1. [file elife-78430-fig5-data1.pptx]

## Slide 1
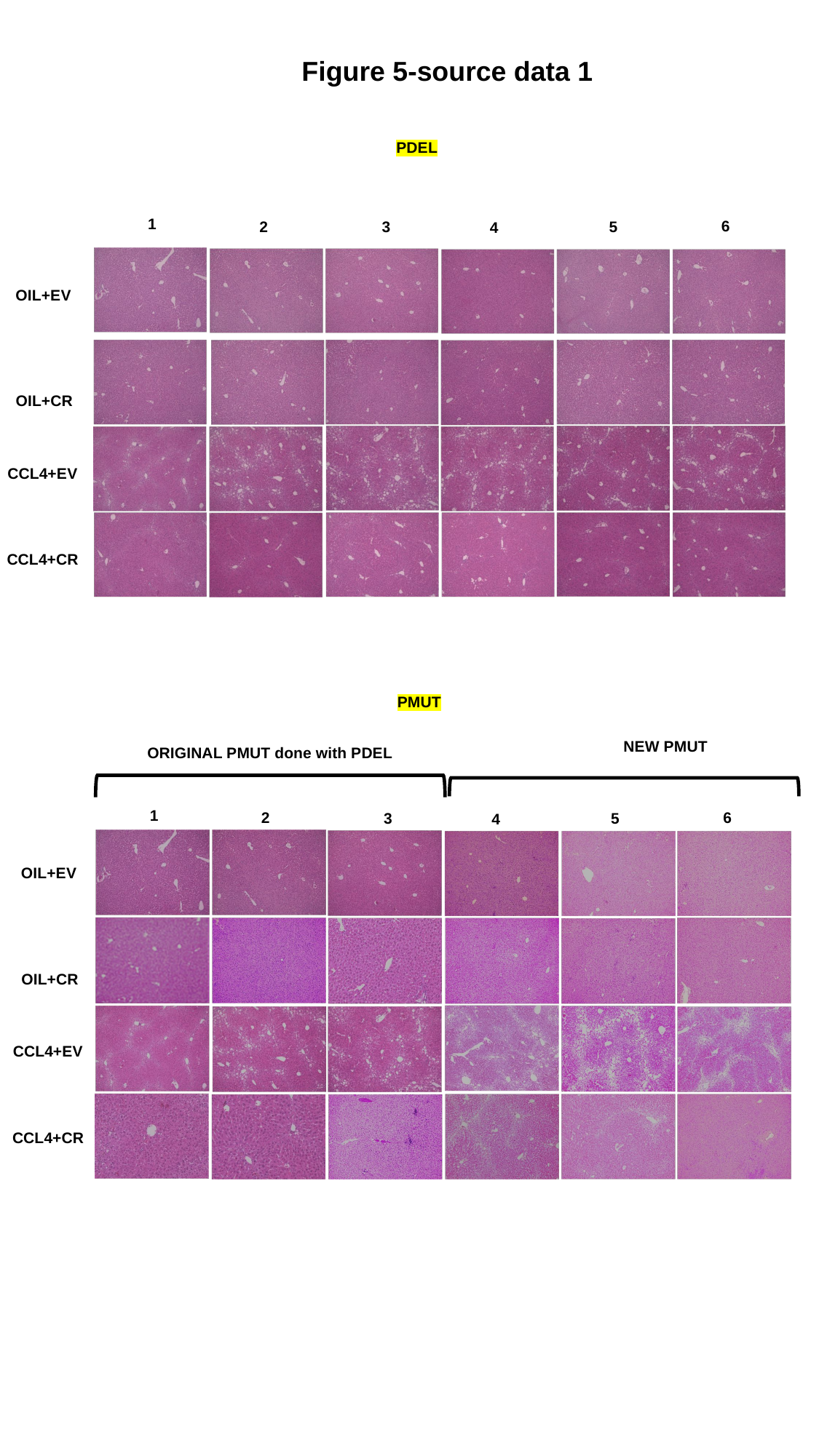

Figure 5-source data 1
PDEL
1
6
2
3
5
4
OIL+EV
OIL+CR
CCL4+EV
CCL4+CR
PMUT
NEW PMUT
ORIGINAL PMUT done with PDEL
1
6
2
3
5
4
OIL+EV
OIL+CR
CCL4+EV
CCL4+CR
