# Supplementary material for: Targeting A-kinase anchoring protein 12 phosphorylation in hepatic stellate cells regulates liver injury and fibrosis in mouse models
Source: eLife. 2022 Oct 4;11:e78430. doi: 10.7554/eLife.78430 (PMC9531947; doi:10.7554/eLife.78430)
Supplement: Supplementary file 1. [file elife-78430-supp1.docx]

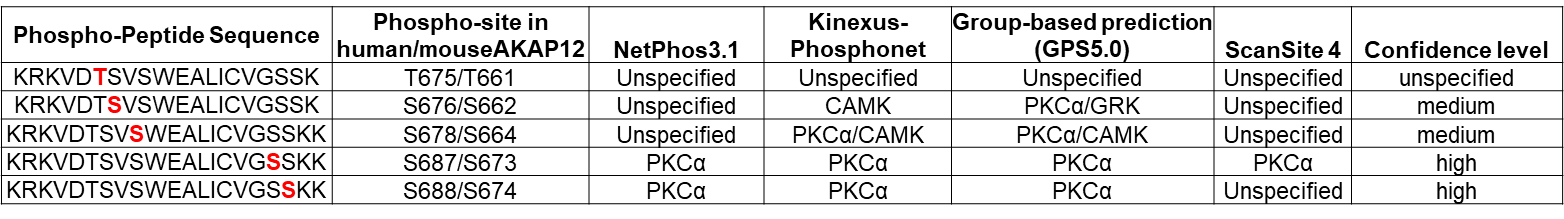


The human or mouse AKAP12 primary sequence was evaluated for predicted kinases from 4 different kinase prediction softwares, NetPhos3.1 (Center for Biological sequence analysis, Denmark), PhosphoNet-Kinexus (Bioinformatics corporation), GPS5.0 (group-based prediction system, version 5.0) and scansite 4.0. The confidence level ranged from low to medium to high based on similarity to the consensus for each kinase. PKCα consensus site with high confidence: [S/T]-X-[R/K]; PKCα consensus site with low/medium confidence: [R/K]-X-[S/T]; calmodulin kinase (CAMK) consensus site: [R]-X-X-[S/T]. S/T= serine/threonine, X=any amino acid, R/K= basic amino acids, Arginine and lysine.

**Supplementary Table S1. Kinase-prediction for AKAP12’s activation-responsive phospho-sites**
